# Comprehensive analysis of palmitoylation-related proteins for prognostic risk evaluation and tumor immune microenvironment assessment in glioma

**DOI:** 10.3389/fimmu.2025.1599769

**Published:** 2025-12-08

**Authors:** Jihao Xue, Li Lin, Chang Liu, Qijia Yin, Tao Wang, Rui Lai, Ligang Chen, Yiran Yin, Ming Wang, Jie Zhou

**Affiliations:** 1 Department of Neurosurgery, The Affiliated Hospital, Southwest Medical University, Luzhou, Sichuan, China; 2 Department of Emergency, The Affiliated Hospital, Southwest Medical University, Luzhou, Sichuan, China; 3 Department of Orthopedics, The Affiliated Hospital, Southwest Medical University, Luzhou, Sichuan, China; 4 Department of Center for Renal Diseases, Sichuan Provincial People's Hospital East Sichuan Hospital and Dazhou First People's Hospital, Dazhou, Sichuan, China

**Keywords:** glioma, palmitoylation, tumor immune microenvironment, risk score, immunotherapy, methylation

## Abstract

**Background:**

Glioma is one of the most common tumors, characterized by a high incidence rate and mortality, posing a formidable global health challenge. Palmitoylation represents a significant post-translational modification that holds a pivotal role in the progression of glioma. However, the biological mechanisms underlying palmitoylation-related genes (PRGs) in glioma remain elusive to date.

**Methods:**

This study utilized an unsupervised clustering algorithm based on the TCGA-GBMLGG cohort to identify palmitoylation-related molecular subtypes and comparatively analyzed the differences between the two subtypes in terms of clinicopathological characteristics, tumor microenvironment (TME), response to immunotherapy, and somatic mutations. Subsequently, through LASSO Cox regression analysis, a palmitoylation-related risk score (PRRS) model for predicting the prognosis of glioma patients was developed and validated. Additionally, the differences in chemotherapeutic drug sensitivity and response to immunotherapy among different PRRS groups were evaluated. Ultimately, potential drugs targeting palmitoylation-related proteins for the treatment of glioma were explored through molecular docking studies, molecular dynamics simulations, and in vitro drug experiments.

**Results:**

This study found that compared with glioma patients in Cluster 2, those in Cluster 1 had a higher World Health Organization (WHO) grade and a worse prognosis. Additionally, the infiltration levels of M2-type macrophages and regulatory T cells were higher in Cluster 1 than in Cluster 2. Immune checkpoint genes, major histocompatibility complex (MHC), and T-cell stimulators were also upregulated in Cluster 1. The PRRS model shows promising prospects in predicting the prognosis of glioma patients, and patients with lower PRRS values are more likely to benefit from immunotherapy. Molecular docking, molecular dynamics simulations, and in vitro drug experiments have confirmed that AT-7519, BIX02189, and THZ-2-101-1 can inhibit glioma cell migration while promoting cell apoptosis.

**Conclusions:**

A significant correlation exists between palmitoylation and tumor microenvironment in glioma. The PRRS emerges as a dependable prognostic biomarker, offering therapeutic advantages in the context of chemotherapy and immunotherapy, and potentially aiding in clinical decision-making for glioma patients. The identified compounds, AT-7519, BIX02189, and THZ-2-101-1, may potentially exert inhibitory effects on the malignant progression of glioma by targeting palmitoylation-related proteins.

## Introduction

Glioma, as the most prevalent primary malignant neoplasm within the adult central nervous system, poses a persistent challenge in clinical research and therapeutic interventions, characterized by its high incidence and unfavorable prognosis (1, 2). The remarkable heterogeneity and infiltrative nature of these tumors render traditional cancer treatment modalities, encompassing surgical resection, radiotherapy, and chemotherapy, ineffective in achieving definitive therapeutic outcomes. In light of these challenges, the World Health Organization (WHO) has meticulously classified glioma into five distinct categories based on their histological attributes: adult-type diffuse glioma, pediatric-type diffuse low-grade glioma (LGG), pediatric-type diffuse high-grade glioma (HGG), circumscribed astrocytic glioma, and ependymal tumors (3). The most common and highly malignant form of glioma is glioblastoma multiforme (GBM). Despite a multidisciplinary treatment approach encompassing maximal surgical resection, adjuvant radiotherapy, and chemotherapy, the extension of survival remains limited (4). Early diagnosis and effective therapeutic intervention are paramount for enhancing the outcomes of glioma patients. Consequently, there is an urgent need to gain a comprehensive understanding of the mechanisms driving glioma progression, identify novel prognostic biomarkers, and develop more efficacious treatment modalities.

S-palmitoylation constitutes a reversible post-translational lipid modification and stands as one of the most common such modifications, serving to modulate the subcellular localization, stability, and functional properties of diverse proteins (5). Protein palmitoylation entails the covalent linkage of fatty acyl chains to internal cysteine residues within proteins via thioester bonds, a process that is regulated by palmitoyl S-acyltransferases from the zinc finger Asp-His-His-Cys-type (ZDHHC) family (6, 7). The tumor microenvironment (TME) constitutes a sophisticated and dynamic ecosystem, exerting profound impacts on the initiation and progression of tumor cells and exhibiting a tight correlation with the therapeutic efficacy of immunotherapy as well as the prognosis of patients (8, 9). Currently, a growing body of evidence indicates that ZDHHC-mediated protein palmitoylation occupies a pivotal position in modulating the immune microenvironment and tumor progression, particularly in the context of glioma development and malignant progression. Specifically, ZDHHC23 and ZDHHC18 can regulate the cell plasticity of various GBM subtypes in particular conditions, which aids in the survival of tumor cells in a stressful TME and the transition of glioma stem cells in GBM (10). Moreover, the palmitoylation of EZH2 mediated by ZDHHC5 facilitates glioma progression, while it also orchestrates the palmitoylation of FAK to augment epithelial-to-mesenchymal transition (EMT) in GBM cells, thereby potentiating their invasive and tumorigenic characteristics (11, 12). Zhao et al. discovered that ZDHHC4 can palmitoylate GSK3β, altering its phosphorylation level and triggering the EZH2/STAT3 axis to increase GBM TMZ-resistance and glioblastoma stem cells (GSCs) tumorigenicity (13). Additionally, the phosphorylation of ZDHHC8 mediated by AMPKα1 promotes the palmitoylation of SLC7A11, thereby enhancing resistance to ferroptosis in GBM (14). Elucidating the genes and characteristics related to palmitoylation of proteins, as well as identifying biomarkers pertinent to this modification, is of paramount importance for the management and prognostic assessment of glioma. Palmitoylation can affect immune cell infiltration and tumor immune landscape, both of which help determine the effectiveness of immunotherapy in glioma patients and further understand how palmitoylation-related genes (PRGs) lead to immune escape and treatment resistance. Consequently, we utilized bioinformatics approaches and predictive modeling algorithms to assess the connection between palmitoylation and TME, concurrently prioritizing the investigation of the prognostic significance of PRGs.

In this study, palmitoylation-related molecular subtypes were identified based on the TCGA-GBMLGG cohort using unsupervised clustering algorithms. Differences between the two palmitoylation subtypes in terms of clinicopathological features, TME, immunotherapy response, and somatic mutations were compared. Furthermore, we have developed a prognostic model for glioma patients based on palmitoylation-related risk score (PRRS) and validated its prognostic utility in forecasting patient outcomes as well as their responsiveness to chemotherapy and immunotherapy. An extensive drug sensitivity assessment was conducted, complemented by molecular docking studies and molecular dynamics simulations, to predict potential small-molecule compounds targeting proteins associated with palmitoylation. Experimental validation was also performed for these candidate drugs. The results of this study will offer deeper insights into the impact of palmitoylation on glioma and contribute to enhancing the efficacy of personalized therapies for glioma patients.

## Materials and methods

### Data acquisition

**Figure 1:**
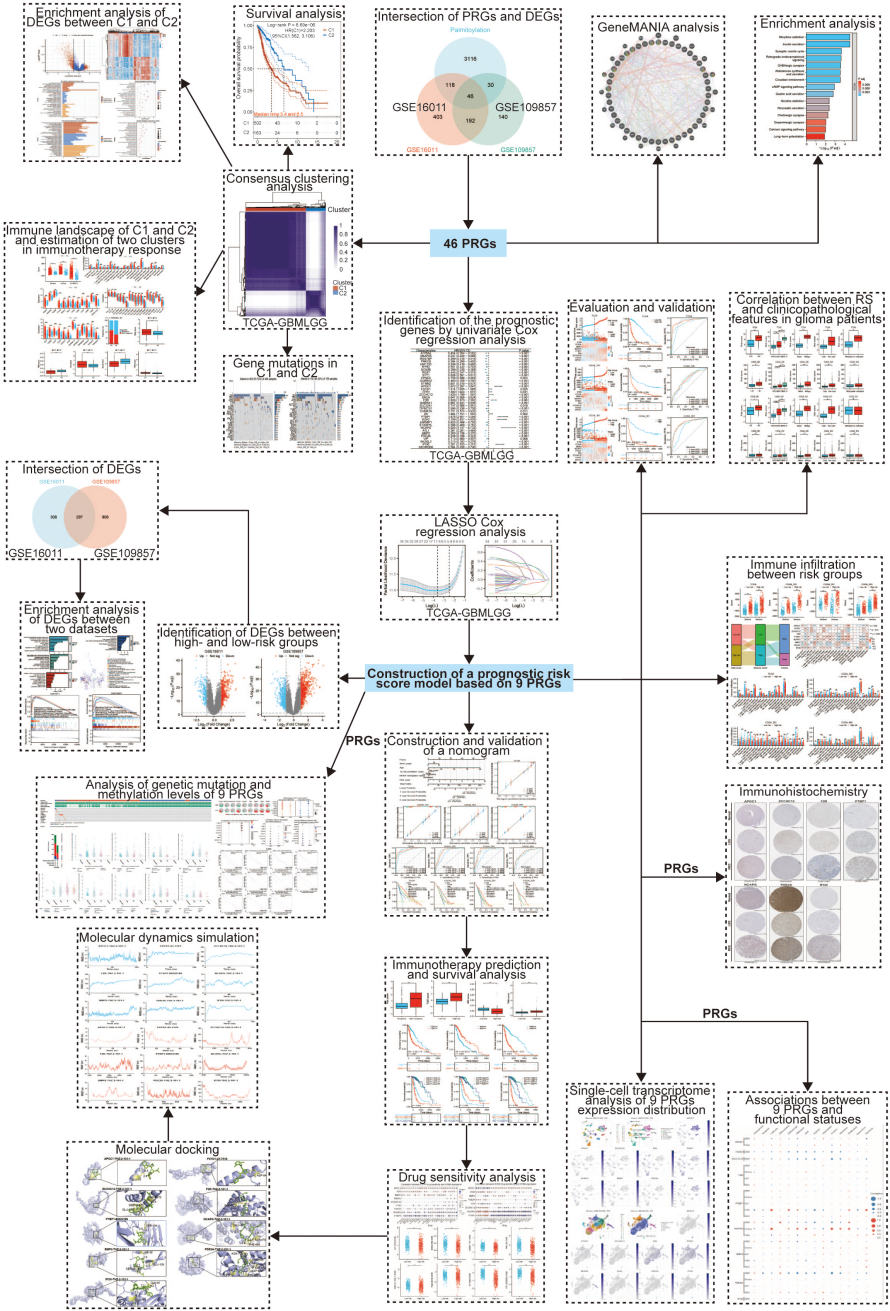
Workflow of the study.

The Cancer Genome Atlas (TCGA) database (https://portal.gdc.cancer.gov/) provided us with RNA sequencing, corresponding clinicopathology, and genomic mutation data for glioma patients. 1152 normal brain tissue sample information was collected from the genotype-tissue expression (GTEx) database (https://www.gtexportal.org/home/index.html). Similarly, the RNA-sequencing expression data and matched clinical information were downloaded from the GSE16011 and GSE109857 datasets of the Gene Expression Omnibus (GEO) repository (https://www.ncbi.nlm.nih.gov/gds/) and from the CGGA_301, CGGA_325, and CGGA_693 datasets of the Chinese Glioma Genome Atlas (CGGA) database (http://www.cgga.org.cn/). A total of 3310 genes associated with palmitoylation were retrieved from GeneCards database (www.genecards.org). The raw count data were normalized using the “limma” package in the R software (Version 4.2.0) (15). The flowchart of this study design is shown in **Figure 1**.

### Differential analysis and functional enrichment analysis and biological networks

The “limma” R package was utilized to identify differentially expressed genes (DEGs), applying the threshold of “|log2FC | > 2 and FDR < 0.05” (16). We identified the overlapping DEGs associated with palmitoylation in datasets GSE16011 and GSE109857, presenting them through a Venn diagram. GeneMANIA (http://genemania.org/) (17) was used to build the gene-gene interaction network. Functional enrichment analysis, including Kyoto encyclopedia of genes and genomes (KEGG) and Gene Ontology (GO), were performed using the “ClusterProfiler” package (18). Gene set enrichment analysis (GSEA) was also used to evaluate the biological activity and pathways in high-risk and low-risk glioma patients. The gene sets corresponding to various pathways were obtained from the Molecular Signatures Database (MSigDB). To quantify the enrichment level and statistical reliability, the normalized enrichment score (NES) and false discovery rate (FDR) were utilized, respectively (19). Simultaneously, Metascape database (https://metascape.org/) integrates functional enrichment, interactome analysis, gene annotation, and membership search to offer experimental biologists a comprehensive means of analyzing and interpreting gene expression profiles. Additionally, NetworkAnalyst 3.0 (https://www.networkanalyst.ca/) was utilized to construct the coregulatory networks of transcription factor (TF)-microRNA (miRNA) for PRGs.

### Consensus clustering analysis

Utilizing the “ConsensusClusterPlus” package in R (20), unsupervised hierarchical clustering was performed on glioma patients, classifying them into the most appropriate number of clusters based on the palmitoylation-associated intersection genes. The “PCA” package was utilized to conduct principal component analysis (PCA) across various subgroups.

### Immunogenomic landscape analysis

The stromal score, immune score, and estimate score were calculated by the “ESTIMATE” R package for samples in the TCGA-GBMLGG, CGGA_301, CGGA_325, and CGGA_693 cohorts. Tumor-infiltrating immune cells (TIICs) were assessed in the TCGA-GBMLGG, CGGA_301, CGGA_325, and CGGA_693 cohorts using the CIBERSORT online tool (21). The two-sample Wilcoxon test was employed to compare immune infiltration and functions among various groups. We further compared the gene expression differences of common immune checkpoint inhibitors (ICIs), major histocompatibility complex (MHC), and T-cell stimulators between different clusters. A method for assessing the effectiveness of immune checkpoint blockade (ICB) is the Tumor Immune Dysfunction and Exclusion (TIDE) score, which is available on the TIDE website (http://tide.dfci.harvard.edu/) (22). A range of predictive indicators for the ICIs response, encompassing the TIDE score, microsatellite instability (MSI) score, T-cell exclusion score, and T-cell dysfunction score, were utilized to assess the correlation between various clusters and the efficacy of immunotherapy, as well as the relationship between different PRRS groups and the outcomes of immunotherapy.

### Tumor mutation analysis

The waterfall plots were utilized, produced by the “maftools” R package (23), to evaluate the somatic point mutation counts in each sample within the TCGA-GBMLGG cohort, allowing for comparisons of genetic variations among distinct clusters. Kaplan-Meier survival analysis was performed incorporating both TMB and subgroup classification, categorizing patients into four prognostic groups to assess the survival disparities between those with high versus low TMB values within distinct subgroups.

### Construction of the palmitoylation-related risk score

Based on the intersection of PRGs and DEGs, univariate Cox regression analysis was used to evaluate the prognostic significance of these genes, and genes with *P* values less than 0.05 were selected for further analysis. To further refine the selection of candidate prognostic-related genes, we employed the LASSO Cox regression algorithm via the R package “glmnet” to discern variations in the regression coefficients of prognostic genes (24). 9 PRGs with nonzero coefficients were selected through 10-fold cross-validation. We designated the TCGA-GBMLGG cohort as the training set, with CGGA_301, CGGA_325, and CGGA_693 serving as independent test sets. The PRRS formula is as follows: *Risk score* = Σ (Coefi × Exp) Coefi indicates the risk coefficient, and Exp indicates the expression level. We calculated the risk score (RS) for each patient and divided them into high-risk and low-risk groups based on the median RS. A risk curve was produced to explore the differences in survival status between patients in different risk groups. Moreover, based on both training and test datasets, we performed Kaplan-Meier survival analysis on risk scores and generated time-dependent receiver-operating characteristic curves to appraise the predictive ability of the risk groups identified above. The R package “pheatmap” was used to create heatmaps that showed the variation in PRGs expression as well as the distribution pattern across clinicopathological traits and risk score groups.

### Development and evaluation of the clinical prognostic model

To assess whether the risk score serves as an independent prognostic factor influencing the survival of glioma patients, we performed univariate and multivariate analysis on the TCGA-GBMLGG dataset. Nomograms are extensively utilized in cancer prognosis research, serving as statistical predictive models that integrate multiple risk factors to estimate the individual survival probabilities (25). Utilizing the TCGA-GBMLGG dataset, we constructed a prognostic nomogram model to predict the 1-, 3-, and 5-year overall survival (OS) probabilities employing the “rms” package in R. Based on the training and test datasets, calibration curves were plotted for graphical assessment. The concordance index (C-index) and the receiver operating characteristic (ROC) analysis were employed to assess the predictive accuracy of the nomogram, while the decision curve analysis (DCA) was utilized to evaluate the clinical utility of the nomogram.

### Differential expression analysis of PRGs

The protein expression levels corresponding to PRGs screened by the LASSO Cox regression algorithm in glioma were explored through the UALCAN data analysis portal (https://ualcan.path.uab.edu/). In the Human Protein Atlas (HPA) database (https://www.proteinatlas.org/), the protein expression differences of PRGs in glioma of different malignant degrees and normal tissues were compared based on immunohistochemical images.

### Immune characteristics analysis of PRGs

The TIMER database (http://timer.cistrome.org) (26) was utilized to evaluate the correlations between the expression levels of PRGs and the infiltration rates of B cells, CD8+ T cells, CD4+ T cells, macrophages, neutrophils, and dendritic cells. The infiltration enrichment of 24 common immune cells was presented using the ssGSEA method from the R package “GSVA” (27). In addition, the relationships between PRGs and immunomodulator expression levels in GBM and LGG were examined using the TISIDB database (http://cis.hku.hk/TISIDB/index.php). Ultimately, the expression status of PRGs across various cell types within the tumor microenvironment (TME) was investigated based on RNA sequencing datasets from 10x Genomics (GSE131928 and GSE163108) and Smart-seq2 (GSE89567 and GSE84465), retrieved through the Tumor Immune Single-cell Hub 2 (TISCH2) database (http://tisch.compbio.cn/) (28).

### Functional analysis of PRGs

The cancerSEA database was employed to ascertain the mean correlation between PRGs and various functional states in glioma, encompassing angiogenesis, apoptosis, cell cycle, differentiation, DNA damage, DNA repair, epithelial-mesenchymal transition (EMT), hypoxia, inflammation, invasion, metastasis, proliferation, quiescence, and stemness.

### Genetic alteration and methylation analysis

The mutational landscape of PRGs in glioma was investigated utilizing the cBioPortal database (http://cbioportal.org) (29). We conducted an in-depth analysis of the heterozygous and homozygous copy number variations (CNV) of PRGs, as well as the correlation between the CNV and mRNA expression levels of the respective genes via the GSCALite platform (http://bioinfo.life.hust.edu.cn/web/GSCALite/). Furthermore, the platform was employed to investigate the correlation between PRGs methylation and their expression profiles in glioma, as well as to assess the influence of varying methylation states on survival outcomes. MethSurv (https://biit.cs.ut.ee/methsurv/) is an online program that performs variable survival analysis using DNA methylation data. Hypomethylation at certain CpG sites in glioma was explored for its prognostic significance.

### Chemotherapy drug sensitivity analysis among different PRRS groups

Based on the GSCALite website, we studied the correlations between PRGs expression levels and drug response. Considering the absence of biomarkers capable of precisely forecasting chemotherapeutic drug sensitivity in glioma patients, we utilized the “pRRophetic” and “ggplot2” packages to perform drug susceptibility analysis, comparing the half-maximal inhibitory concentration (IC50) values of various chemotherapeutic agents against glioma between high- and low-risk groups via the Wilcoxon signed-rank test.

### Molecular docking and molecular dynamics simulations

Utilizing Autodock (https://ccsb.scripps.edu/mgltools/downloads/) for molecular docking analysis allowed for the investigation of the interactions between the identified PRGs and potential therapeutic compounds. The small molecular drugs and protein structures corresponding to the PRGs were sourced from the PubChem website (https://pubchem.ncbi.nlm.nih.gov/) and PDB database (https://www.rcsb.org/), respectively. Ultimately, biological macromolecules and small molecular drugs were automatically docked, adhering to the standardized docking procedure. The results were visualized using the PyMol. A 100 ns molecular dynamics (MD) simulation was conducted using Gromacs software to further substantiate the plausibility and dependability of the docking outcomes (30). In order to evaluate the binding stability of the receptor-ligand complex, MD simulation was carried out after achieving equilibrium in a human-like environment using the TIP3P water model, which represents a 0.145 mol/L neutral sodium chloride solution. The physical conditions were set to constant pressure (101 kPa), constant temperature (310 K), and periodic boundary conditions (31, 32). Conformations were recorded and computed every 10 ps. The root mean square deviation (RMSD) and root mean square fluctuation (RMSF) of the MD simulation results analysis and visualization were performed using a GROMACS inbuilt program with the (Equation 1) RMSD and (Equation 2) RMSF formula as follows.

(1) $$RMSD=\sqrt{\frac{1}{N}\sum_{i=1}^{i=N}{\left(R_{t}-R_{ref}\right)}^{2}}$$

(2) $$RMSF=\sqrt{\frac{1}{T}\sum \;{\left(R_{t}-R_{ref}\right)}^{2}}$$

In the above equations, $R_{t}-R_{ref}$ denotes the displacement of the t-th atom at a specific frame from its position in the reference conformation (ref) (i.e., positional offset), N stands for the total number of atoms, and T indicates each sampling time point.

### Cell culture and drug treatment

The LN229 and U251 cell lines were obtained from BeNa Culture Collection (BNCC, Henan, China). Cells were cultured in high-glucose DMEM (HyClone, USA) supplemented with 10% fetal bovine serum (FBS, Gibco, USA) and 1% penicillin-streptomycin (PS, Gibco, USA), and the cultures were maintained at 37 °C in a humidified 5% CO_2_ atmosphere. The small-molecule compound was dissolved in dimethyl sulfoxide (DMSO) to prepare a 10 mM stock solution. This stock solution was subsequently subjected to serial twofold dilutions using cell culture medium, thereby establishing a series of concentration gradients. In none of the groups did the final concentration of DMSO surpass 0.1%.

### Cell viability assay

Cell viability was evaluated using the Cell Counting Kit-8 (CCK-8) assay (EpiZyme, Cat. No. CX001M). Cells were seeded into 96-well microplates at a density of 5 × 10³ cells per well and incubated overnight to allow for complete cell adherence. The cells were then cultured in culture medium with varying drug concentrations for 48 hours. Following treatment, 10 µL of CCK-8 reagent was added to each well, followed by incubation at 37 °C for 2 hours. Using a microplate reader (Bio-Rad), the absorbance was measured at 450 nm, cell viability was calculated relative to the control group, and the IC50 value was determined with GraphPad Prism 9.0.

### Wound healing assay

Cells were inoculated into 6-well culture plates and incubated until they attained 95% confluence. Subsequently, a consistent linear scratch was generated using a sterile 200 µL pipette tip. After washing with phosphate-buffered saline (PBS), the cells were incubated in a serum-free culture medium containing a drug concentration equivalent to 0.1 times the IC50 value. Wound area images were captured at 0, 24, and 48 h under a microscope. The scratch area was measured using ImageJ software.

### Flow cytometry

Cell apoptosis was assessed using the Annexin V-FITC/PI Apoptosis Detection Kit (Beyotime, Cat. No. C1062S-2), strictly adhering to the guidelines provided by the manufacturer. After being cultured for 24 hours in a medium with a drug concentration equivalent to 0.1 times the IC50 value, the cells were harvested and then washed with PBS. The cells were resuspended in binding buffer, followed by incubation with 5 µL of Annexin V-FITC and 10 µL of propidium iodide (PI) staining solution at room temperature for 15 minutes in the dark. The CytoFLEX flow cytometer was used to analyze apoptosis as soon as staining was completed.

### Statistical analysis

R software (version 4.1.0, https://www.r-project.org/) was used to perform all statistical analyses in this study. The independent Student’s *t*-test was used to compare continuous variables between two groups, the Wilcoxon rank-sum test was used to compare non-normally distributed variables, and the chi-squared test was used to compare data on categorical variables between two groups. Survival curves were generated employing the Kaplan–Meier method and assessed for comparison through the log-rank test. *P* value< 0.05 was considered statistically significant.

## Results

### Identification of DEGs related to palmitoylation in glioma

**Figure 2:**
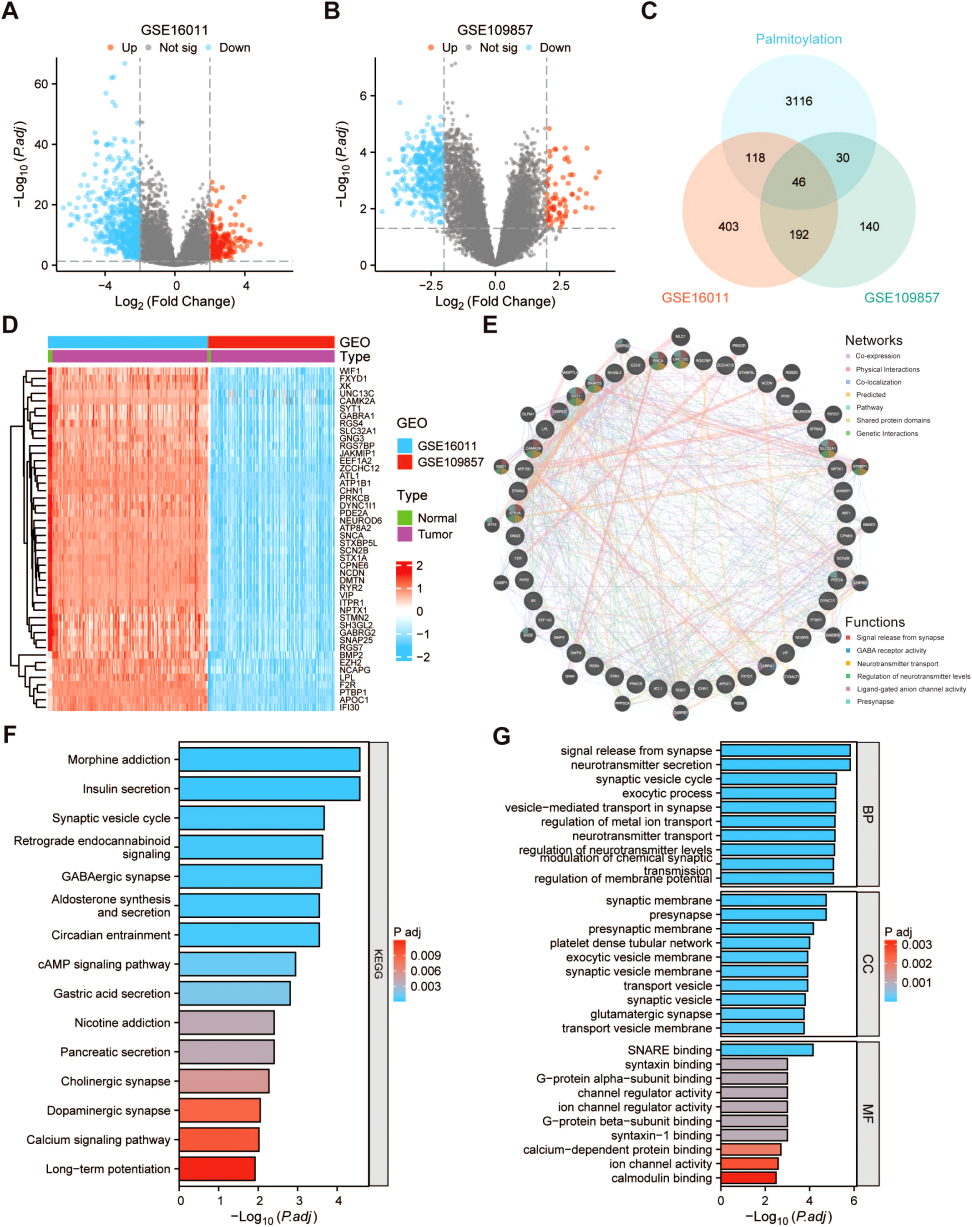
Identification of palmitoylation-related differentially expressed genes (DEGs) in glioma and functional enrichment analysis. Exhibition of DEGs in **(A)** GSE16011 and **(B)** GSE109857 datasets between glioma and normal brain tissue using volcano plots. **(C)** Venn diagram of intersection of DEGs in the GSE16011 and GSE109857 datasets and PRGs. **(D)** Heatmap of 46 intersecting genes in the GSE16011 and GSE109857 datasets. **(E)** Creation of a gene-gene network of 46 DEGs by Genemania. The bar plot of the **(F)** KEGG and **(G)** GO pathways enriched for the DEGs.

The datasets GSE16011 and GSE109857 have been collected and utilized in order to screen for DEGs between glioma and normal brain tissue. 225 genes and 534 genes are significantly upregulated and downregulated in the glioma tissues, respectively, according to the analytical results of the GSE16011 dataset (**Figure 2A**). 76 genes and 332 genes are significantly upregulated and downregulated in the glioma tissues, respectively, according to the analytical results of the GSE109857 dataset (**Figure 2B**). Then, the intersection of DEGs from the two datasets and a set of 3310 PRGs was determined, ultimately identifying 46 common DEGs associated with palmitoylation (**Figure 2C**). **Figure 2D** displays the heatmap of 46 intersecting genes. Furthermore, the 46 DEGs were uploaded to the GeneMANIA database to establish a gene co-expression network, which demonstrated their associated functionalities. As depicted in **Figure 2E**, these genes exhibit close correlations across diverse biological functions, encompassing signal release from synapse, GABA receptor activity, neurotransmitter transport, regulation of neurotransmitter levels, ligand-gated anion channel activity, and presynapse. Subsequently, GO and KEGG enrichment analyses were conducted to investigate the underlying biological roles of the 46 DEGs. Based on KEGG pathway enrichment analysis, the results showed that these genes were primarily involved in morphine addiction, insulin secretion, synaptic vesicle cycle, retrograde endocannabinoid signaling, and GABAergic synapse (**Figure 2F**). Furthermore, GO analysis demonstrated that these genes were mainly connected with signal release from synapse, neurotransmitter secretion, and synaptic vesicle cycle (**Figure 2G**).

### The biological features of each cluster associated with palmitoylation

**Figure 3:**
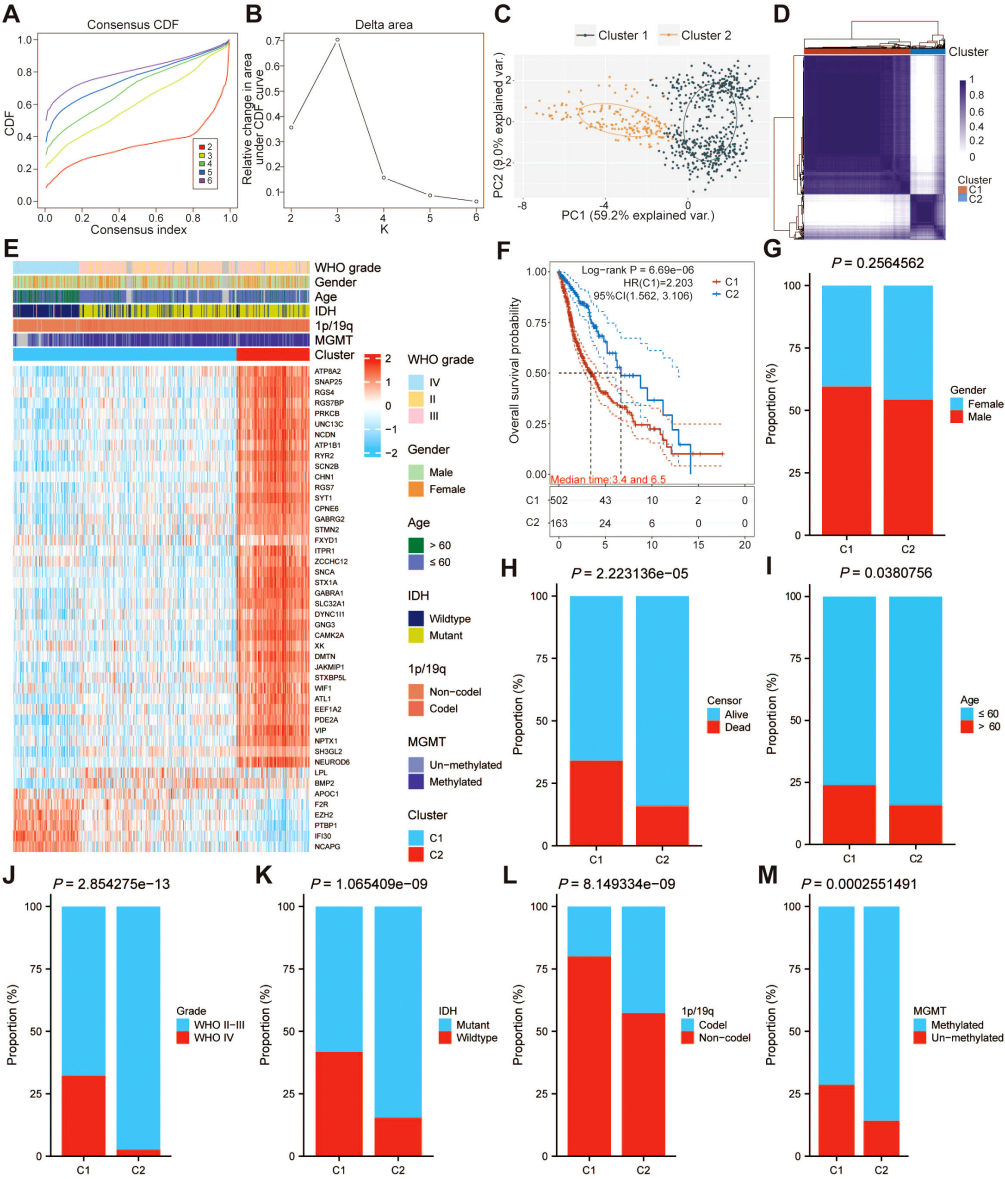
The subgroup analysis in the TCGA-GBMLGG dataset based on 46 palmitoylation-related genes (PRGs). **(A)** Consensus clustering of cumulative distribution function **(CDF)** for k = 2 to 6. **(B)** Relative change in the area under the CDF curve for k = 2 to 6. **(C)** The two subgroups involved in clusters 1 and 2 were subjected to principal component analysis (PCA). **(D)** The TCGA-GBMLGG dataset was divided into two distinct clusters when k = 2. **(E)** Heatmap of distribution of clinicopathological variables between different cluster groups. **(F)** The Kaplan–Meier curve survival analysis between different cluster groups. The differences in **(G)** gender, **(H)** survival rates, **(I)** age, **(J)** WHO grade, **(K)** isocitrate dehydrogenase (IDH) mutation status, **(L)** 1p/19q codeletion status, and **(M)** O6-methylguanine-DNA methyltransferase (MGMT) promoter methylation status between patients in the two clusters were compared.

Given the heterogeneity inherent within tumors, TCGA-GBMLGG patients were divided into two subgroups with different molecular and clinical characteristics by unsupervised consensus analysis, based on the expression levels of 46 palmitoylation-related DEGs, including 502 samples in cluster 1 and 164 samples in cluster 2 (**Figures 3A, B**). PCA revealed that the 46 genes linked to palmitoylation could clearly discriminate between the two clusters (**Figure 3C**). Following that, a heatmap using a consensus clustering solution (k = 2) displays the two clusters (**Figure 3D**). **Figure 3E** shows the distribution of clinical pathological characteristics in clusters 1 and 2, as well as the transcriptomic characteristics of PRGs differentially expressed in the two subtypes. Except for gender, there were statistically significant differences in survival rates, age, WHO grade, isocitrate dehydrogenase (IDH) mutation status, 1p/19q codeletion status, and O6-methylguanine-DNA methyltransferase (MGMT) promoter methylation status between the two clusters (**Figures 3G-M**). In addition, we found that the OS of patients in cluster 2 was significantly better than that of patients in cluster 1 (**Figure 3F**). These suggest that PRGs may exert an influence on the progression of glioma via certain underlying mechanisms. 1223 genes were screened out by DEGs analysis between the two clusters, of which 301 genes were upregulated and 922 genes were downregulated (**Supplementary Figure 1A**). The heatmap was used to display the expression of DEGs based on hierarchical clustering analysis (**Supplementary Figure 1B**). Then, GO enrichment and KEGG pathway analysis were performed on the up- and down-regulated DEGs, respectively (**Supplementary Figures 1C–F**).

**Figure 4:**
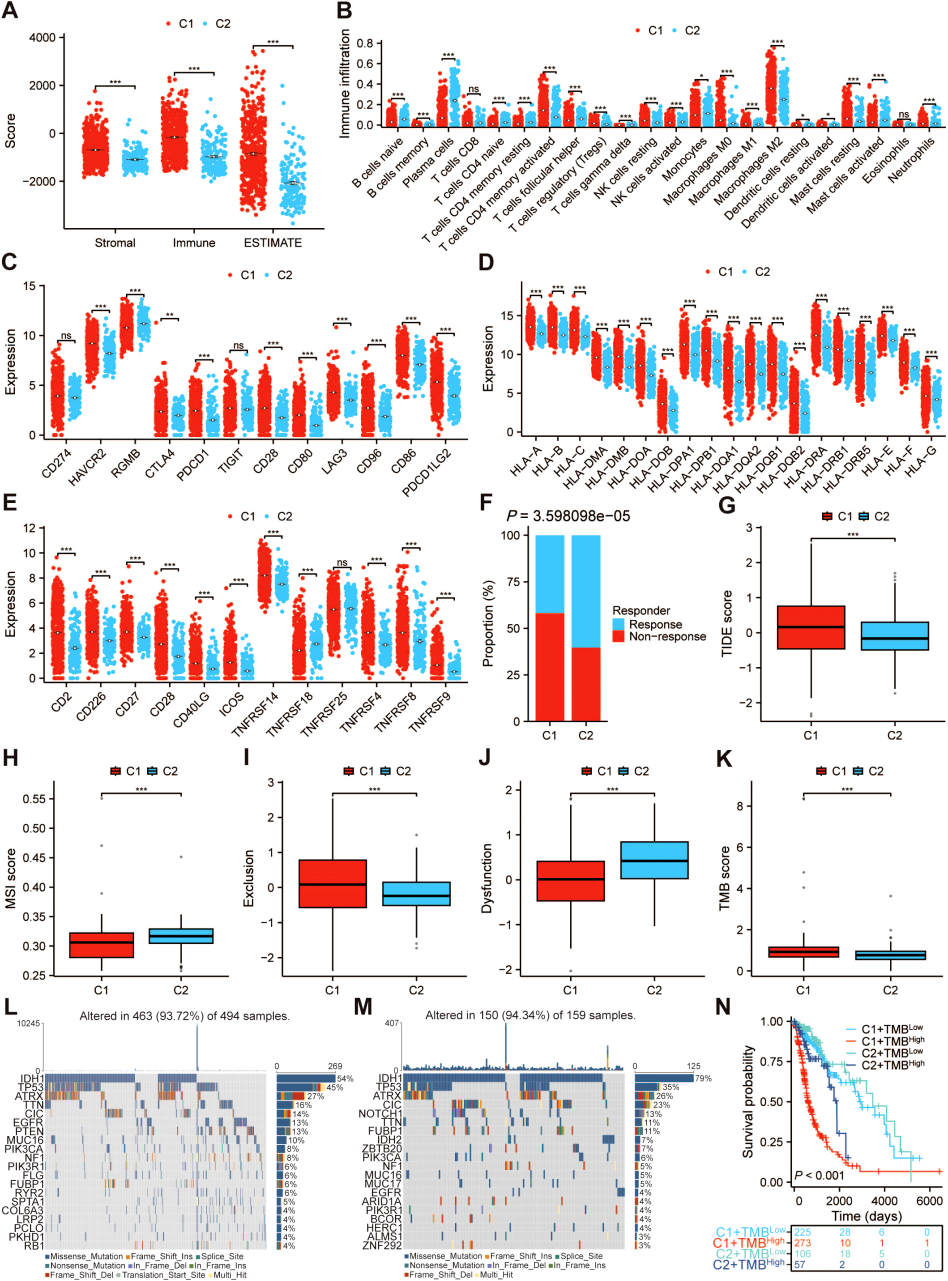
Immune infiltration and mutation analysis in different clusters. **(A)** The comparisons of stromal score, immune score, and estimate score among various clusters. Difference of **(B)** immune cell infiltration, **(C)** immune checkpoint genes, **(D)** MHC, and **(E)** T-cell stimulators between different clusters based on the TCGA-GBMLGG dataset. **(F)** Comparison of the response of different clusters to immunotherapy. Box plot of **(G)** TIDE, **(H)** MSI, **(I)** T-cell exclusion, **(J)** dysfunction, and **(K)** TMB scores between two clusters. Waterfall plot displaying gene mutations in **(L)** cluster 1 and **(M)** cluster 2. **(N)** The Kaplan–Meier curve survival analysis for glioma patients stratified by both TMB score and different clusters. ***p < 0.001. *p < 0.05, **p < 0.01, NS, not significant.

Considering the close relationship between palmitoylation and the tumor microenvironment, the differences in immune cells and immune infiltration between the two clusters were assessed utilizing the ESTIMATE and CIBERSORT algorithms (33). We found that the stromal score, immune score, and estimate score in cluster 1 were significantly higher than those in cluster 2 (**Figure 4A**). Cluster 1 was distinguished by a prominent infiltration of memory B cells, activated CD4+ memory T cells, regulatory T cells (Tregs), resting natural killer (NK) cells, M0 macrophages, M1 macrophages, M2 macrophages, resting dendritic cells (DCs), activated dendritic cells, and resting mast cells. In contrast, Cluster 2 was characterized by a significant infiltration of naive B cells, plasma cells, naive CD4+ T cells, resting CD4+ memory T cells, follicular helper T cells (Tfh), gamma delta T cells (γδ T cells), activated NK cells, monocytes, activated mast cells, and neutrophils (**Figure 4B**). These results indicated that the aforementioned two clusters exhibited distinctly disparate infiltration characteristics within the TME. Then, we studied the relationship between the two clusters and ICIs, MHC, as well as T-cell stimulators. Immune checkpoint genes (*HAVCR2*, *CTLA4*, *PDCD1*, *CD28*, *CD80*, *LAG3*, *CD96*, *CD86*, and *PDCD1LG2*), MHC, and T-cell stimulators (*CD2*, *CD226*, *CD27*, *CD28*, *CD40LG*, *ICOS*, *TNFRSF14*, *TNFRSF4*, *TNFRSF8*, and *TNFRSF9*) were highly expressed in cluster 1 (**Figures 4C–E**). The TIDE algorithm was employed to assess the risk of tumor immune escape. The findings revealed that, when compared with cluster 2, cluster 1 demonstrated a suboptimal response to immunotherapy and a higher TIDE score, suggesting an increased probability of immune escape (**Figures 4F, G**). In addition, we found that the T-cell exclusion score was higher in cluster 1, while the MSI and T-cell dysfunction scores were higher in cluster 2 (**Figures 4H–J**).

TMB exhibits a close correlation with the immune cell infiltration and therapeutic efficacy of immunotherapy (34, 35). The somatic mutation data for patients with TCGA-GBMLGG was acquired, and the TMB scores were subsequently calculated. The results showed that the TMB values in cluster 1 were slightly higher than those in cluster 2 (**Figure 4K**). The mutation differences of the top 20 genes across various glioma clusters were displayed using waterfall plots (**Figures 4L, M**). In glioma patients of clusters 1 and 2, missense mutations were the most prevalent type of mutation (**Supplementary Figures 2Aa, Ba**), single nucleotide polymorphism (SNP) held an absolute position in contrast to insertion (INS) or deletion (DEL) (**Supplementary Figures 2Ab, Bb**), and the most common mutation type identified was C>T (**Supplementary Figures 2Ac, Bc**). **Supplementary Figures 2Ad, Bd** displayed the number of mutations per sample. A type of mutation was represented by the box diagram for each color in **Supplementary Figures 2Ae, Be**. The horizontal histograms illustrated genes with a higher mutation frequency, such as in cluster 1 (*IDH1* (54%), *TP53* (45%), *ATRX* (27%), *TTN* (16%), *CIC* (14%)) and cluster 2 (*IDH1* (79%), *TP53* (35%), *ATRX* (26%), *CIC* (23%), *NOTCH1* (13%)) (**Supplementary Figures 2Ae, Be**). Given the potential correlation between palmitoylation-related clusters and TMB, we conducted a stratified analysis and found that the integration of clusters with TMB scores offers a more accurate prediction of the prognosis for glioma patients (**Figure 4N**). Furthermore, given the pivotal roles that N6-methyladenosine (m^6^A) methylation and ferroptosis play within the immune microenvironment, we conducted an investigation into the expression profiles of m^6^A-associated genes and ferroptosis-related genes across distinct clusters. The results showed that most m^6^A-related genes and ferroptosis-related genes were highly expressed in cluster 1 (**Supplementary Figures 2C, D**).

### Construction and validation of the prognostic PRRS model

**Figure 5:**
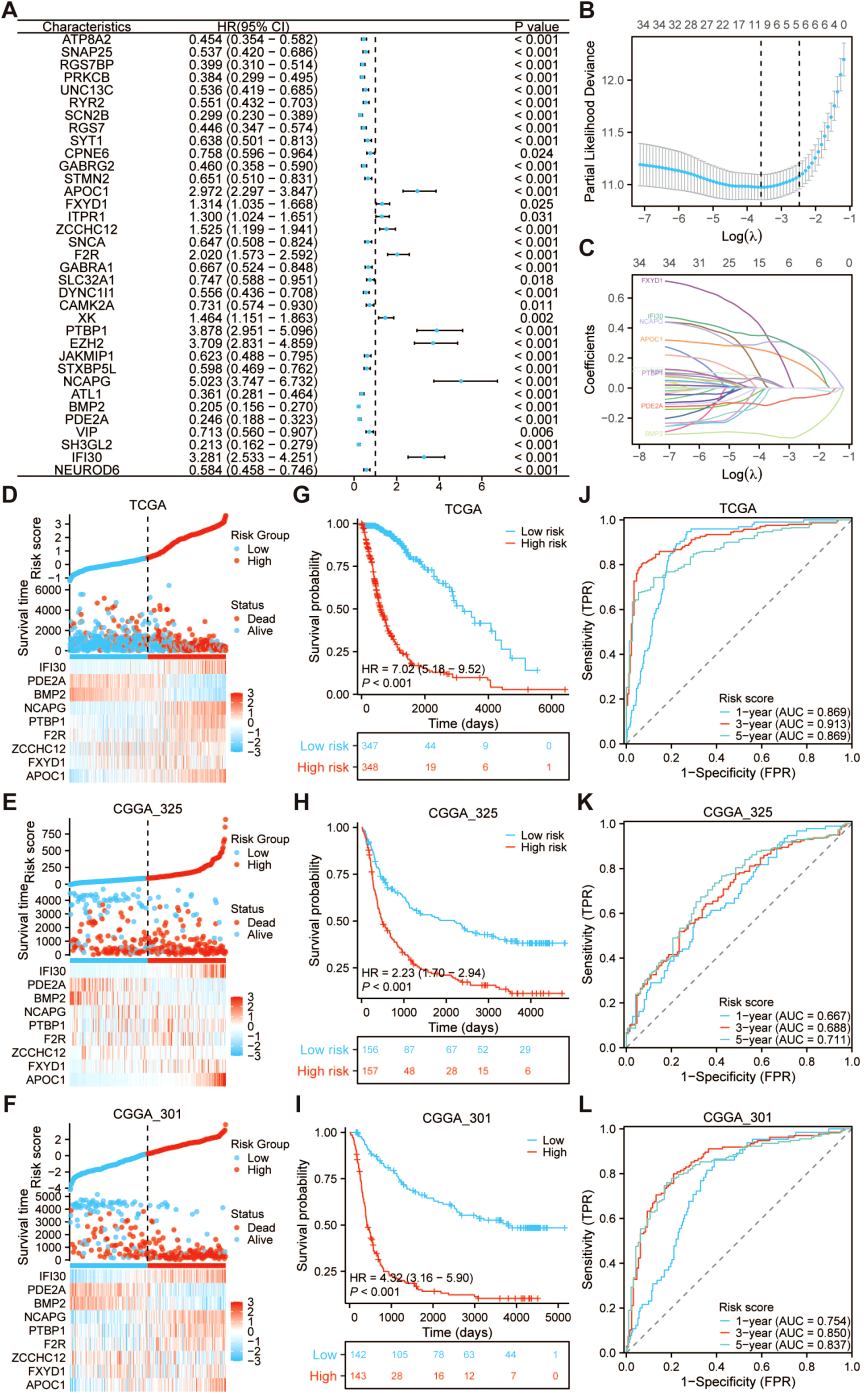
Construction and validation of the palmitoylation-related risk score (PRRS) model. **(A)** Univariate Cox regression analysis of PRGs in the TCGA-GBMLGG dataset, identifying significant prognostic markers. **(B-C)** LASSO Cox regression analysis for dimensionality reduction and optimal gene panel selection in prognostic modeling. Correlation between PRRS and patient survival risk in the **(D)** TCGA-GBMLGG, **(E)** CGGA_325, and **(F)** CGGA_301 datasets, suggesting that higher risk scores are linked to a higher chance of death. Kaplan–Meier survival curves for low- and high-risk groups in the **(G)** TCGA-GBMLGG, **(H)** CGGA_325, and **(I)** CGGA_301 datasets. Time-dependent ROC curve analysis for predicting 1-, 3-, and 5-year overall survival in the **(J)** TCGA-GBMLGG, **(K)** CGGA_325, and **(L)** CGGA_301 datasets.

Based on the TCGA-GBMLGG cohort, a univariate Cox regression analysis was conducted on 46 DEGs, resulting in the identification of 35 PRGs that were significantly associated with prognosis (**Figure 5A**). The LASSO Cox regression analysis was employed to filter the best prognostic indicators from 35 PRGs. After incorporating variables into the LASSO Cox regression model corresponding to the minimum lambda value, 9 PRGs were selected for the construction of the prognostic PRRS model (**Figures 5B, C**). The PRRS was computed using the following formula: *risk score* = (0.237684096 * *APOC1*) + (0.296416314 * *FXYD1*) + (0.033378972 * *ZCCHC12*) + (0.031624620 * *F2R*) + (0.043403341 * *PTBP1*) + (0.308436072 * NCAPG) + (-0.317672694 * *BMP2*) + (-0.108996577 * *PDE2A*) + (0.342006851 * *IFI30*). The risk curve analysis conducted within the TCGA-GBMLGG, CGGA_325, and CGGA_301 cohorts demonstrated a correlation between elevated risk scores and an increased risk of mortality (**Figures 5D–F**). The outcomes of the Kaplan-Meier survival analysis have confirmed that the survival prospects for patients in the high-risk group are markedly inferior to those in the low-risk group. The *p*-values obtained from both the training cohort (TCGA-GBMLGG) and the validation cohorts (CGGA_325 and CGGA_301) were all statistically significant, being less than 0.001 (**Figures 5G–I**). A more in-depth time-dependent ROC curve analysis was subsequently performed to ascertain the robustness of the model. The results demonstrated its robust predictive capability for 1-year, 3-year, and 5-year survival rates, which was further corroborated in the validation cohort (**Figures 5J–L**). These findings underscore the potential efficacy of the model in clinical risk assessment scenarios.

### Correlation between PRRS and clinicopathological characteristics

**Figure 6:**
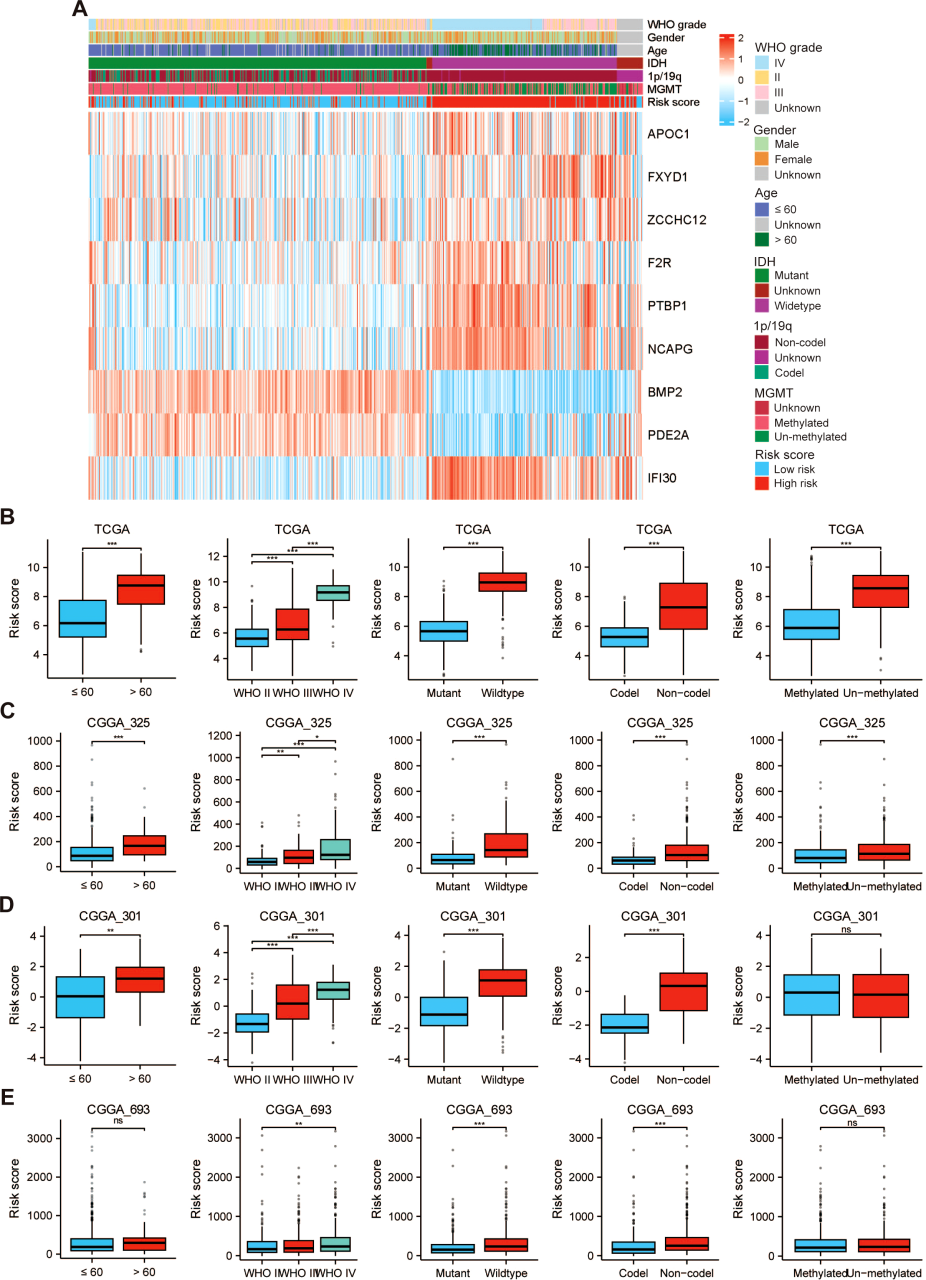
The PRRS was associated with the clinicopathological characteristics of patients with glioma in the TCGA-GBMLGG dataset. **(A)** Heatmap for the PRRS and clinicopathological manifestation. Boxplot of PRRS in glioma patients with different ages, WHO grades, IDH mutation status, 1p/19q codeletion status, and MGMT promoter methylation status among **(B)** TCGA-GBMLGG, **(C)** CGGA_325, **(D)** CGGA_301, and **(E)** CGGA_693 datasets. *p < 0.05, **p < 0.01, ***p < 0.001, NS, not significant.

We observed a significant correlation between the risk score and factors including age, WHO grade, IDH mutation status, 1p/19q codeletion status, and MGMT promoter methylation status within the TCGA-GBMLGG cohort (**Figure 6A**). Specifically, glioma patients older than 60 years have a higher risk score. A higher WHO grade is associated with an increased RS among patients. Individuals with wild-type IDH demonstrate higher risk scores. Patients presenting with 1p/19q non-coding deletions show elevated risk scores. Furthermore, patients with non-methylated MGMT promoters exhibit higher risk scores (**Figure 6B**). Similarly, these observations were corroborated in the external datasets, namely CGGA_325, CGGA_301, and CGGA_693 (**Figures 6C–E**).

### Development and assessment of the nomogram model

**Figure 7:**
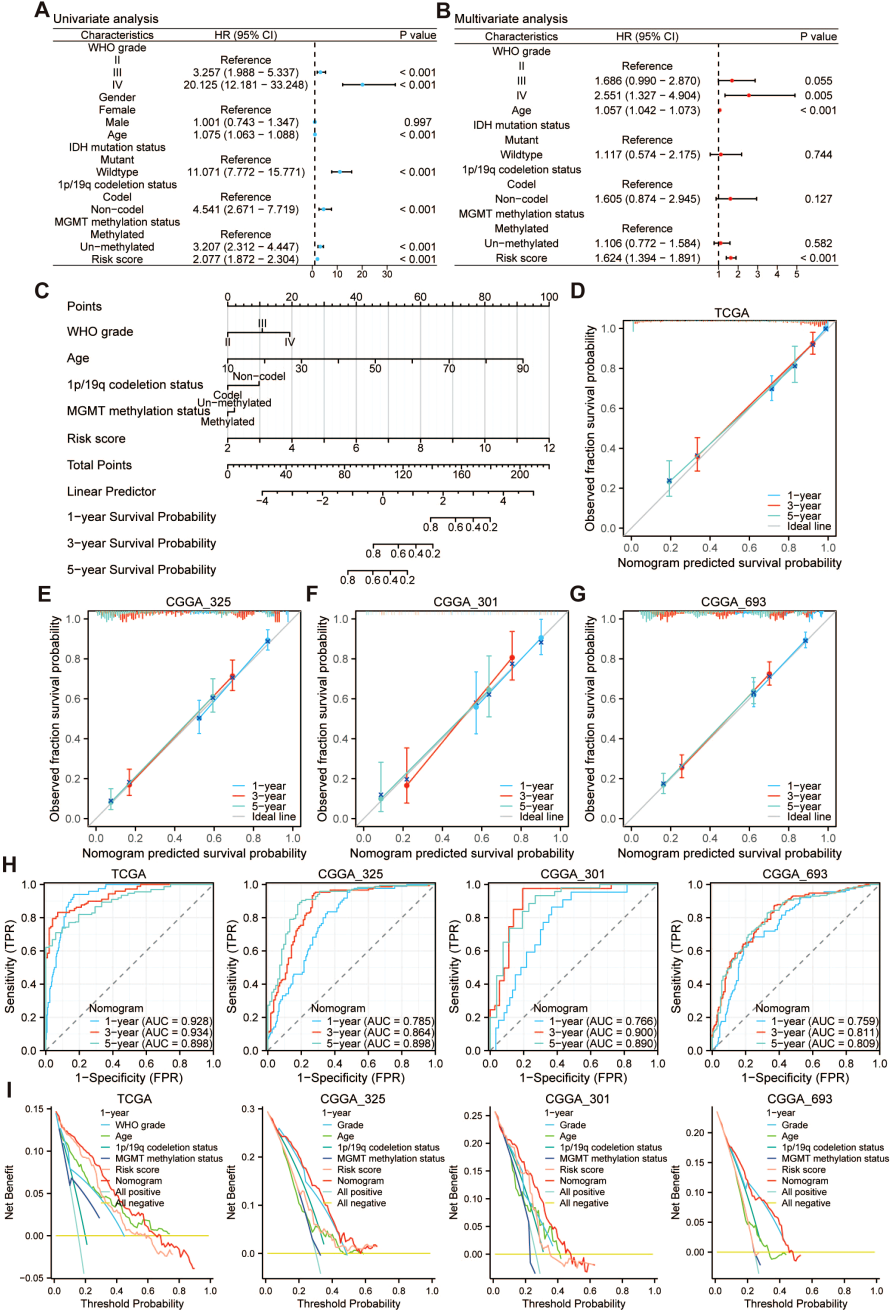
Development and assessment of the nomogram for overall survival prediction. In the TCGA-GBMLGG dataset, **(A)** univariate and **(B)** multivariate Cox regression analysis demonstrated that PRRS is an independent prognostic factor influencing the prognosis of glioma patients. **(C)** The nomogram was developed to predict overall survival at 1, 3, and 5 years by integrating PRRS with additional clinicopathological characteristics based on the TCGA-GBMLGG dataset. Calibration curves of the nomogram model in the **(D)** TCGA-GBMLGG, **(E)** CGGA_325, **(F)** CGGA_301, and **(G)** CGGA_693 datasets. **(H)** ROC curves analysis of the nomogram at 1, 3, and 5 years based on multiple datasets. **(I)** DCA curves derived from multiple datasets to evaluate the accuracy and clinical applicability of the nomogram model for 1-year overall survival of glioma patients.

Due to the significant correlation between PRRS and high malignancy in glioma, it has been demonstrated that PRRS was an independent predictor of survival outcomes through univariate and multivariate Cox regression analysis based on the TCGA-GBMLGG cohort (**Figures 7A, B**). To evaluate the prognostic utility of combining PRRS with clinicopathological parameters, we constructed a nomogram based on WHO grade, age, 1p/19q codeletion status, MGMT promoter methylation status, and PRRS to predict the 1-, 3-, and 5-year survival rates of glioma patients (**Figure 7C**). The results of the proportional hazards (PH) (36) assumption testing for the multivariate Cox regression model showed that both the nomogram and the included variables conformed to the PH assumption (**Supplementary Table 1**). The C-index of the nomogram was 0.884 in the TCGA-GBMLGG cohort, 0.749 in the CGGA_325 cohort, 0.761 in the CGGA_301 cohort, and 0.731 in the CGGA_693 cohort, respectively. The calibration curves derived from multiple cohorts demonstrated excellent concordance between the survival probabilities predicted by the nomogram and those observed in reality (**Figures 7D–G**). The area under the ROC curve (AUC) values were greater than 0.75 at multiple time points, indicating that the model had satisfactory discriminative capacity (**Figure 7H**). DCA further demonstrated that the overall net benefit derived from the nomogram exceeded that of any individual clinical characteristic (**Figure 7I**). The above results suggest that the nomogram we established has good prognostic value for glioma patients and may have practical significance for clinical decision-making.

### Kaplan–Meier survival and differential expression analysis of 9 PRGs

Kaplan-Meier survival analysis revealed a statistically significant correlation between the 9 genes incorporated in the PRRS model and overall survival in the TCGA-GBMLGG cohort, highlighting their crucial prognostic significance (**Supplementary Figure 3A**). The differential expression analysis results showed that the mRNA expression levels of *FXYD1*, *ZCCHC12*, and *PDE2A* were highly expressed in normal brain tissues, while the other six genes were significantly upregulated in glioma samples (**Supplementary Figure 3B**). We further assessed the protein expression levels corresponding to nine PRGs utilizing the UALCAN and HPA databases. Regrettably, data pertaining to protein expression and immunohistochemical outcomes for certain PRGs were deficient in these two databases. The results of the UALCAN database showed that in GBM, the protein expression of APOC1, F2R, PTBP1, NCAPG, and IFI30 significantly increased, while the protein expression of PDE2A significantly decreased (**Supplementary Figure 3C**). The immunohistochemical findings within the HPA database further elucidated the differences in protein expression levels of PRGs between normal brain tissue and LGG and HGG samples (**Supplementary Figure 4**). These findings lend credence to the possible involvement of these PRGs in the development of glioma as well as their value in forecasting patient outcomes.

### PRRS model-based functional analysis

**Figure 8:**
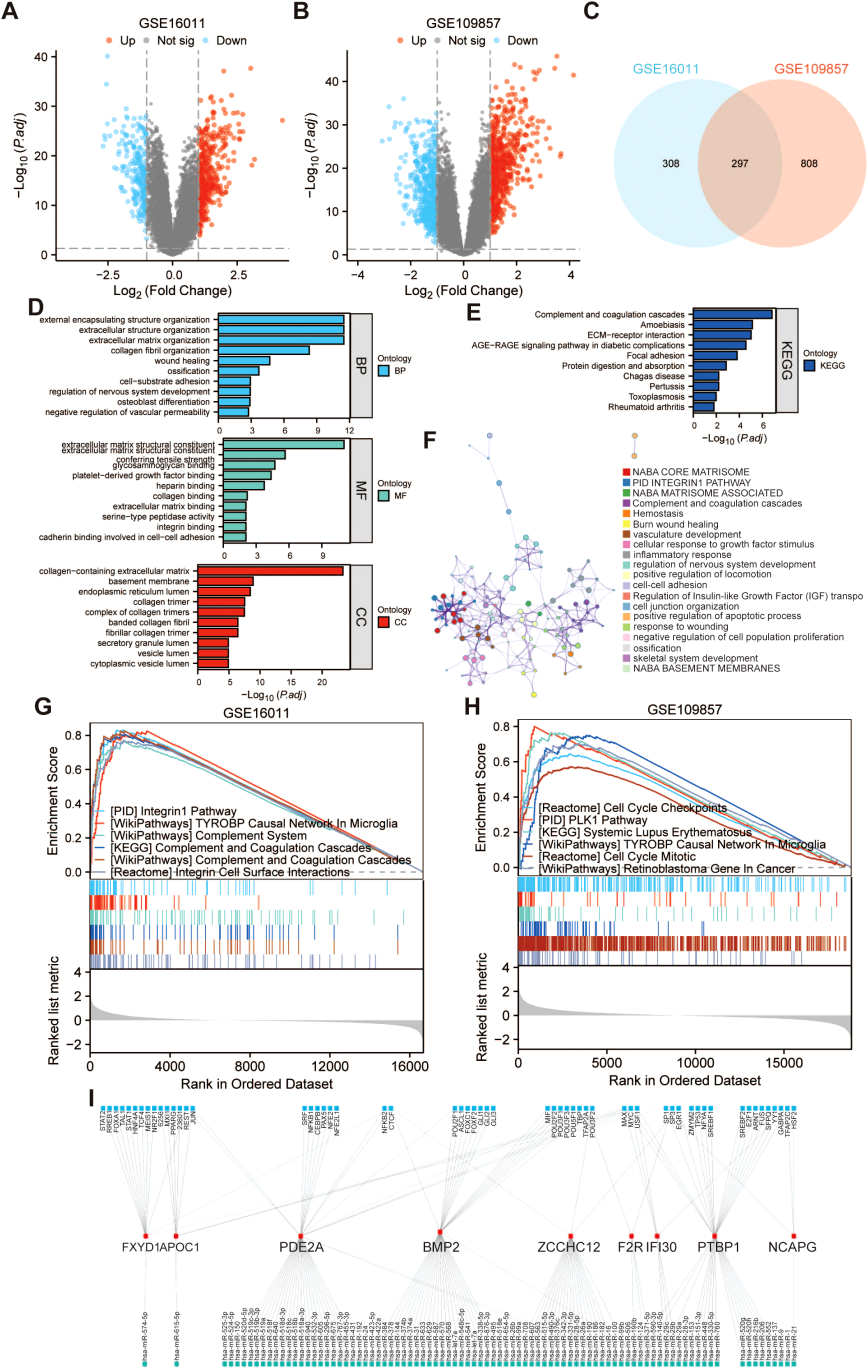
Functional analysis based on the PRRS model. Exhibition of DEGs in **(A)** GSE16011 and **(B)** GSE109857 datasets between the low-risk and high-risk groups using volcano plots. **(C)** Venn diagram displaying intersection DEGs from GSE16011 and GSE109857 datasets. The bar plot of the **(D)** GO and **(E)** KEGG pathways enriched for the DEGs between the high-risk and low-risk groups. **(F)** A visual analysis of the pathways enriched by these DEGs utilizing Metascape. On the basis of these DEGs, additional GSEA analysis was conducted inside the **(G)** GSE16011 and **(H)** GSE109857 datasets. **(I)** Transcription factor-miRNA coregulatory network of 9 PRGs.

For a more in-depth analysis of the differences in gene functions and pathways involved among the subgroups classified by PRRS, 605 and 1105 DEGs were determined between the low-risk and high-risk groups in the GSE16011 dataset and GSE109857 dataset, respectively, with 297 genes being common to both datasets (**Figures 8A–C**). Following that, KEGG pathway analysis and GO enrichment analysis were carried out on these DEGs in the TCGA-GBMLGG dataset. The results of enrichment analysis indicated that these DEGs were predominantly enriched in pathways such as external encapsulating structure organization, extracellular structure organization, and extracellular matrix organization within the biological process category, as well as in pathways associated with complement and coagulation cascades and extracellular matrix (ECM)-receptor interaction within the KEGG category (**Figures 8D, E**). The Metascape enrichment results were colored by cluster ID, where nodes sharing the same cluster ID were usually close to each other. The enrichment results encompassed NABA CORE MATRISOME, PID INTEGRIN1 PATHWAY, complement and coagulation cascades, cellular response to growth factor stimulus, and inflammatory response (**Figure 8F**). Additional GSEA analysis was undertaken within the GSE16011 and GSE109857 datasets to supplement and verify the functional annotations derived from KEGG and GO pathways. The most enriched pathways in the GSE16011 dataset were the integrin1 pathway, TYROBP causal network in microglia, complement system, complement and coagulation cascades. The most enriched pathways in the GSE109857 dataset were the cell cycle checkpoints, PLK1 pathway, systemic lupus erythematosus, and TYROBP causal network in microglia (**Figures 8G, H**). Additionally, the coregulatory network of TF-miRNA for the 9 PRGs was established utilizing NetworkAnalyst (**Figure 8I**).

### Differences in immune infiltration among risk groups

**Figure 9:**
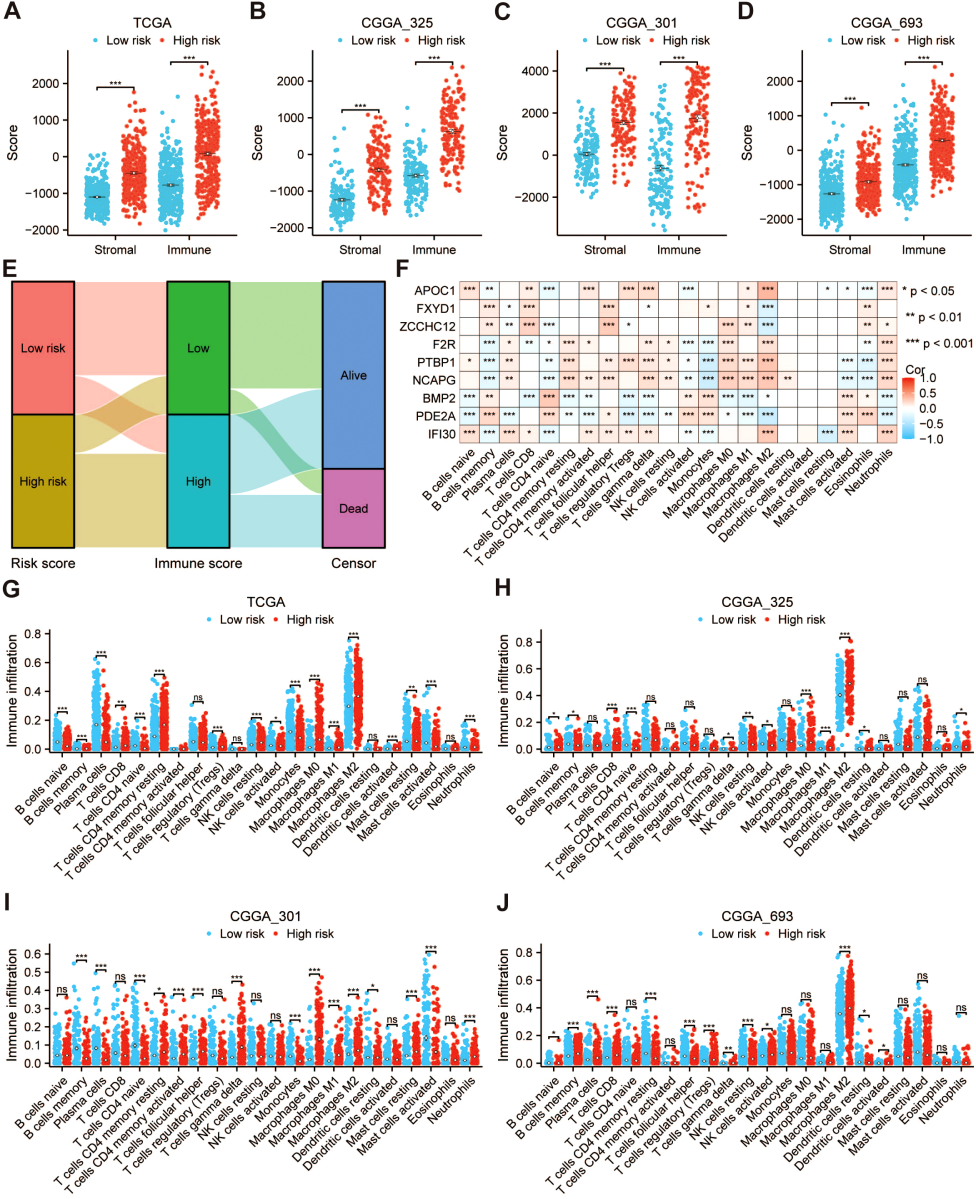
Differences in immune infiltration among risk groups. The stromal and immune scores of the high- and low-risk groups in the **(A)** TCGA-GBMLGG, **(B)** CGGA_325, **(C)** CGGA_301, and **(D)** CGGA_693 cohorts were compared. **(E)** The Sankey diagram of risk score, immune score, and survival status. **(F)** Correlation heatmap of PRGs and different immune cells. Difference of immune cell infiltration between high- and low-risk groups based on the **(G)** TCGA-GBMLGG, **(H)** CGGA_325, **(I)** CGGA_301, and **(J)** CGGA_693 cohorts. *p < 0.05, **p < 0.01, ***p < 0.001, NS, not significant.

We evaluated the differences in immune infiltration between different risk groups in the training cohort (TCGA-GBMLGG) and the validation cohorts (CGGA_325, CGGA_301, and CGGA_693), respectively. The findings revealed that both the immune and stromal scores were higher in the high-risk group compared to those in the low-risk group (**Figures 9A–D**). High immune score corresponded to low survival rate (**Figure 9E**). Then, we compared the differences in TIICs, ICIs, MHC, and T-cell stimulators between different risk groups in multiple cohorts. The results of the training and validation cohorts showed that the abundance of M2 macrophages in the high-risk group was significantly higher than that in the low-risk group (**Figures 9G–J**). The mRNA expression levels of immune checkpoint genes, including *CD274*, *HAVCR2*, *CTLA4*, *PDCD1*, *TIGIT*, *CD80*, *LAG3*, *CD96*, and *PDCD1LG2*, were notably elevated in the high-risk group relative to the low-risk group, whereas the mRNA level of *RGMB* was conspicuously decreased (**Supplementary Figures 5A-D**). Moreover, with the exception of *HLA-DOB* and *HLA-DQA1*, the mRNA levels of MHC were significantly higher in the high-risk group compared to the low-risk group (**Supplementary Figures 5E-H**). Lastly, the mRNA expression levels of T-cell stimulators such as *CD2*, *CD226*, *CD27*, *ICOS*, *TNFRSF14*, *TNFRSF18*, *TNFRSF4*, and *TNFRSF8* were notably increased in the high-risk group when compared to the low-risk group (**Supplementary Figures 5I-L**). In addition, 9 PRGs were correlated with the infiltration of various TIICs (**Figure 9F**).

### Correlation between expression levels of PRGs and tumor immune cell infiltration

Accumulating evidence indicates that the infiltration of immune cells within the TME plays a pivotal role in tumor progression (37). However, it remains unclear whether elevated expression of PRGs influences the recruitment of immune cells in glioma. An analysis conducted utilizing the TIMER database revealed a positive correlation between the expression levels of *APOC1* and *IFI30* in GBM and the abundance of infiltrating B cells, CD8+ T cells, macrophages, and neutrophils. Similarly, in LGG, the expression levels of *APOC1*, *PTBP1*, *NCAPG*, and *IFI30* exhibited a positive correlation with the infiltration of CD4+ T cells, macrophages, neutrophils, and dendritic cells (**Supplementary Figure 6**). Subsequently, we constructed lollipop plots to illustrate the associations between 9 PRGs and the infiltration levels of 24 immune cell types in glioma. The expression levels of *APOC1* and *IFI30* were positively correlated with the infiltration rates of most immune cells (**Supplementary Figure 7**). TIICs residing within the microenvironment are capable of secreting a diverse array of cytokines, thereby making them essential to the progression of glioma. We visualized the relationship between the expression levels of 9 PRGs and various cytokines associated with infiltrating immune cells in the form of heatmaps. The findings demonstrated that the majority of immunostimulators, immunoinhibitors, MHC molecules, chemokines, and chemokine receptors were positively related to the expression levels of *APOC1* and *IFI30* in GBM and LGG (**Supplementary Figures 8A-E**). The expression of important genes is changed by DNA methylation, which impacts the development of glioma (38). Therefore, we investigated the DNA methylation associated with PRGs, and the results showed that the methylation of APOC1 and IFI30 was negatively correlated with most cytokines (**Supplementary Figure 8F-J**). These results unveil new glioma treatment targets and prognostic indicators.

### Single-cell analysis of 9 PRGs

**Figure 10:**
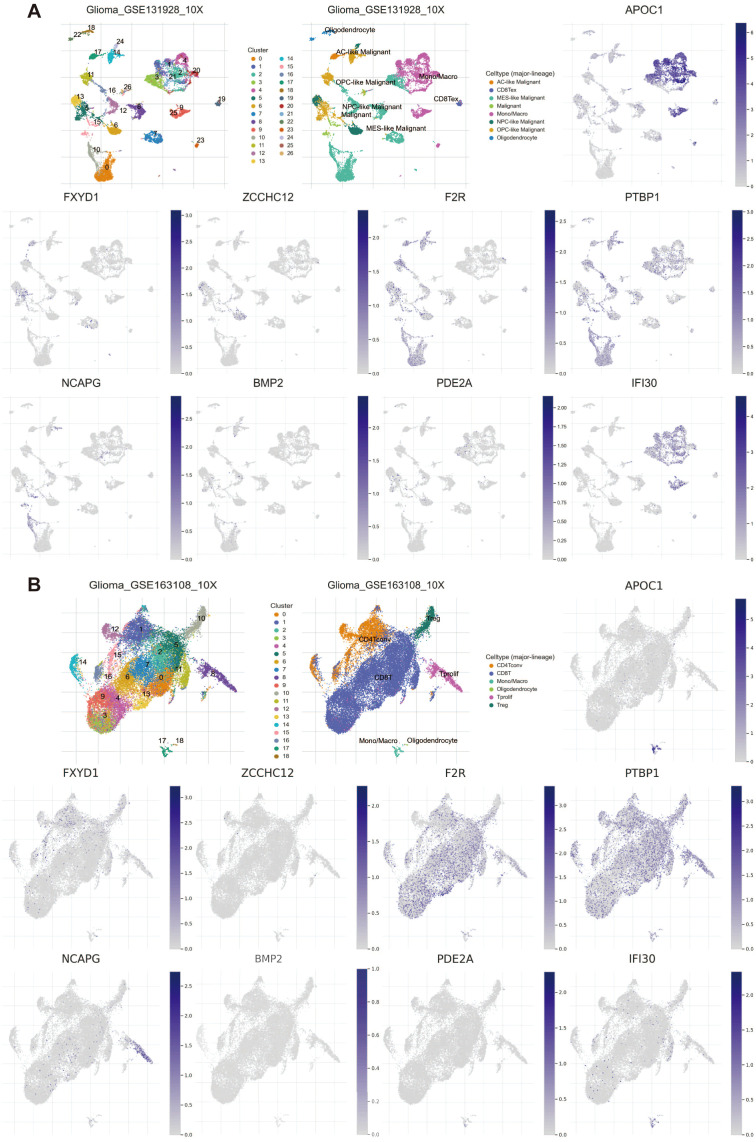
Single-cell transcriptome analysis of PRGs expression distribution in multiple cell types in tumor microenvironment. The expression state of 9 PRGs in multiple cell types based on **(A)** GSE131928 and **(B)** GSE163108 datasets.

Given the prognostic significance and abnormal distribution of PRGs in glioma tissues, we conducted an investigation into the cell types in which these genes are enriched based on single-cell RNA sequencing (scRNA-seq) data. Analysis of the scRNA-seq data in the GSE131928 dataset revealed the identification of 27 cell clusters and 8 cell types in glioma tissues. We observed significant enrichment of *APOC1* and *IFI30* in monocytes/macrophages (**Figure 10A**). 19 cell clusters and 6 cell types were identified in glioma tissues by scRNA-seq data analysis of the GSE163108 dataset. It was observed that *APOC1* and *IFI30* were markedly enriched within monocytes/macrophages, notably in the C17 cluster. Similarly, *NCAPG* exhibited significant enrichment in Tprolif cells, predominantly in the C8 cluster. Furthermore, notable enrichments of *F2R* and *PTBP1* were identified within conventional CD4+ T cells, CD8+ T cells, Tprolif cells, and regulatory T cells (**Figure 10B**). To substantiate our discoveries, we conducted an analysis of the scRNA-seq datasets GSE89567 and GSE84465. Consistently, our results demonstrated a significant enrichment of *APOC1* and *IFI30* within monocytes/macrophages (**Supplementary Figures 9A, B**).

To further explore the functional significance of PRGs in glioma, single-cell analysis was conducted using the CancerSEA database, revealing a significant correlation between PRGs expression and pivotal cellular functions at the single-cell level. The research results found that in HGG, *PTBP1* was positively correlated with the cell cycle, DNA damage, DNA repair, proliferation, and stemness, while negatively correlated with angiogenesis, hypoxia, inflammation, and quiescence. *FXYD1*, *ZCCHC12*, and *PDE2A* exhibited a negative correlation with DNA damage, DNA repair, epithelial-mesenchymal transition (EMT), hypoxia, and invasion in GBM. *NCAPG* was positively correlated with cell cycle, DNA damage, DNA repair, EMT, invasion, metastasis, and proliferation in glioma (**Supplementary Figure 10**). The above results suggest that PRGs may be involved in regulating multiple biological functions of glioma cells.

### Analysis of mutation and methylation levels of PRGs in glioma

**Figure 11:**
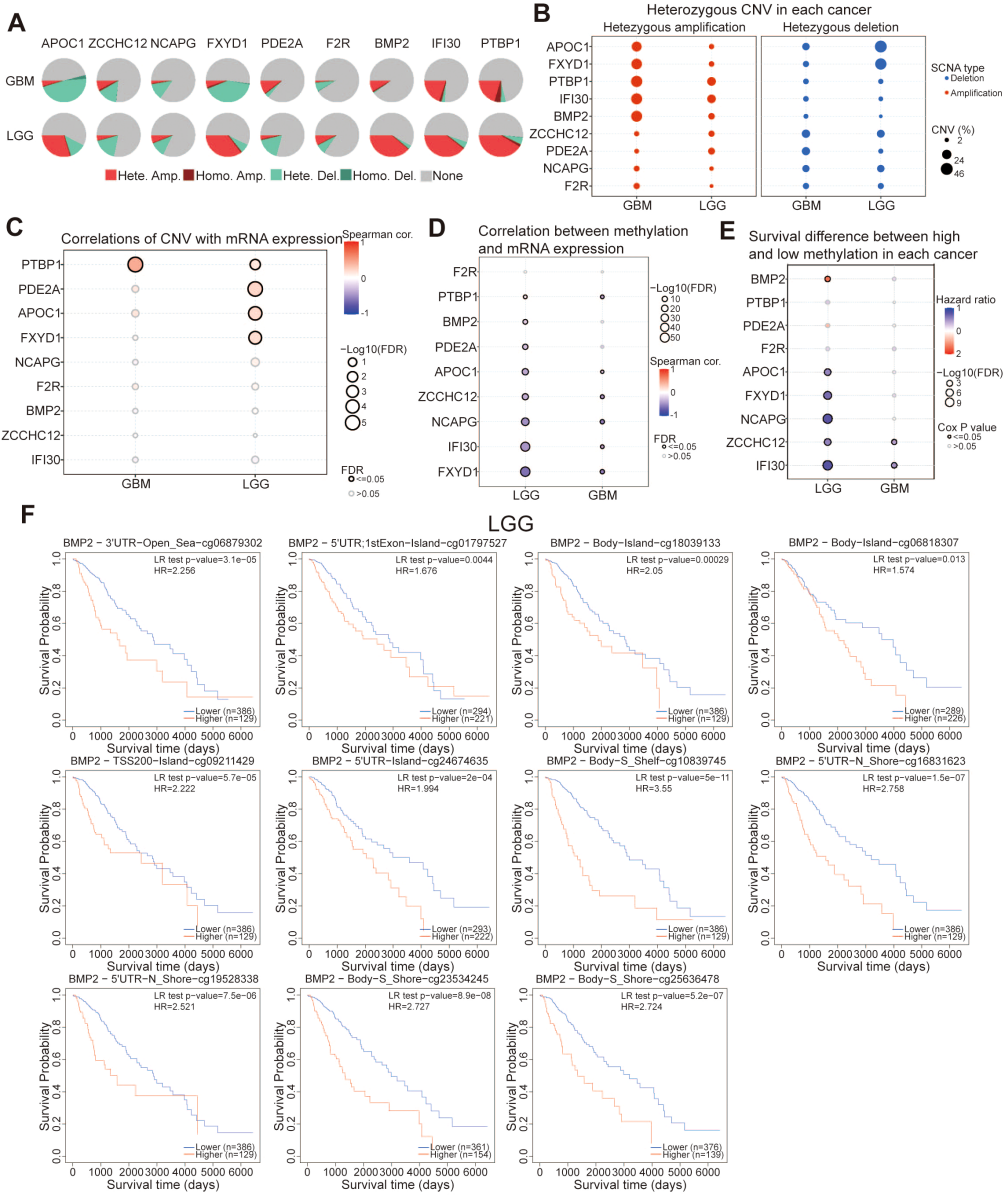
Analysis of mutation and methylation levels of PRGs in glioma. **(A)** Homozygous or heterozygous CNV of PRGs in LGG and GBM. Homo Amp: homozygous amplification; Hete Amp: heterozygous amplification; Homo Del: homozygous deletion; Hete Del: heterozygous deletion; None: without CNV. **(B)** The heterozygous CNV of PRGs and **(C)** the correlation between CNV and mRNA expression. **(D)** Correlation between methylation and PRGs expression in LGG and GBM. **(E)** The difference in overall survival between LGG and GBM caused by hypermethylation and hypomethylation of PRGs. **(F)** The Kaplan–Meier survival analysis of the promoter methylation of BMP2 in LGG.

The genomic variations of 9 PRGs in glioma were analyzed using the cBioPortal database. In the database, 8.6% (95/1099) of glioma patients were found to carry PRGs gene variations, with *PTBP1* accounting for the highest proportion at 2.8% (**Supplementary Figure 11A**). Mutation and amplification constituted the most common genetic alterations observed in PRGs among patients with GBM and LGG (**Supplementary Figure 11B**). Furthermore, we investigated the correlation between the mRNA expression levels and CNV of PRGs (**Supplementary Figure 11C**). Next, we investigated the CNV status of these genes in GBM and LGG. PRGs showed a high sensitivity to mutation, with *APOC1*, *NCAPG*, *FXYD1*, and *F2R* mainly exhibiting heterozygous deletion in GBM, whereas *PDE2A*, *BMP2*, *IFI30*, and *PTBP1* exhibited heterozygous amplification as the main CNV type. In contrast, *ZCCHC12* and *PDE2A* primarily demonstrated heterozygous deletion in LGG, while *APOC1*, *FXYD1*, *BMP2*, *IFI30*, and *PTBP1* showed heterozygous amplification as the main CNV pattern (**Figure 11A**). The susceptibility of these genes to heterozygous deletion and amplification was validated by the CNV analysis in **Figure 11B**. **Figure 11C** presented a notable correlation between CNV and the expression of *PTBP1*, *PDE2A*, *APOC1*, and *FXYD1* in LGG, alongside a significant association between CNV and the expression of *PTBP1* specifically in GBM.

We found that the expression levels of PRGs were negatively correlated with methylation levels. Notably, *APOC1*, *ZCCHC12*, *NCAPG*, *IFI30*, and *FXYD1* showed significant statistical significance, indicating that they were dysregulated (**Figure 11D**). We further analyzed the survival differences between high and low methylation of PRGs, and the results showed that high methylation of *APOC1*, *ZCCHC12*, *NCAPG*, *IFI30*, and *FXYD1* was associated with a lower risk of survival in LGG, while high methylation of *BMP2* indicated a higher risk of survival. Additionally, high methylation of *IFI30* and *ZCCHC12* exhibited a correlation with a lower survival risk in GBM (**Figure 11E**). The influence of methylation levels at various cytosine-phosphate-guanine (CpG) sites on the prognosis in LGG and GBM was further investigated utilizing MethSurv. The results indicated that patients with LGG exhibiting higher methylation levels of *BMP2* demonstrated inferior OS compared to those with lower methylation levels (**Figure 11F**). Conversely, patients with LGG who exhibited higher methylation levels of *APOC1*, *ZCCHC12*, *NCAPG*, *IFI30*, and *FXYD1* displayed superior OS compared to those with lower methylation levels (**Supplementary Figure 12A-E**). Additionally, patients with GBM who harbored higher methylation levels of *IFI30* and *ZCCHC12* exhibited better OS compared to those with lower methylation levels (**Supplementary Figures 13A, B**). In conclusion, there was a negative correlation between the methylation levels of *APOC1*, *NCAPG*, and *IFI30* and their expression levels. It can be inferred that the reduced methylation of *APOC1*, *NCAPG*, and *IFI30* contributed to their elevated expression in glioma, which eventually led to a poor prognosis for glioma patients.

### The role of PRRS in immunotherapy

**Figure 12:**
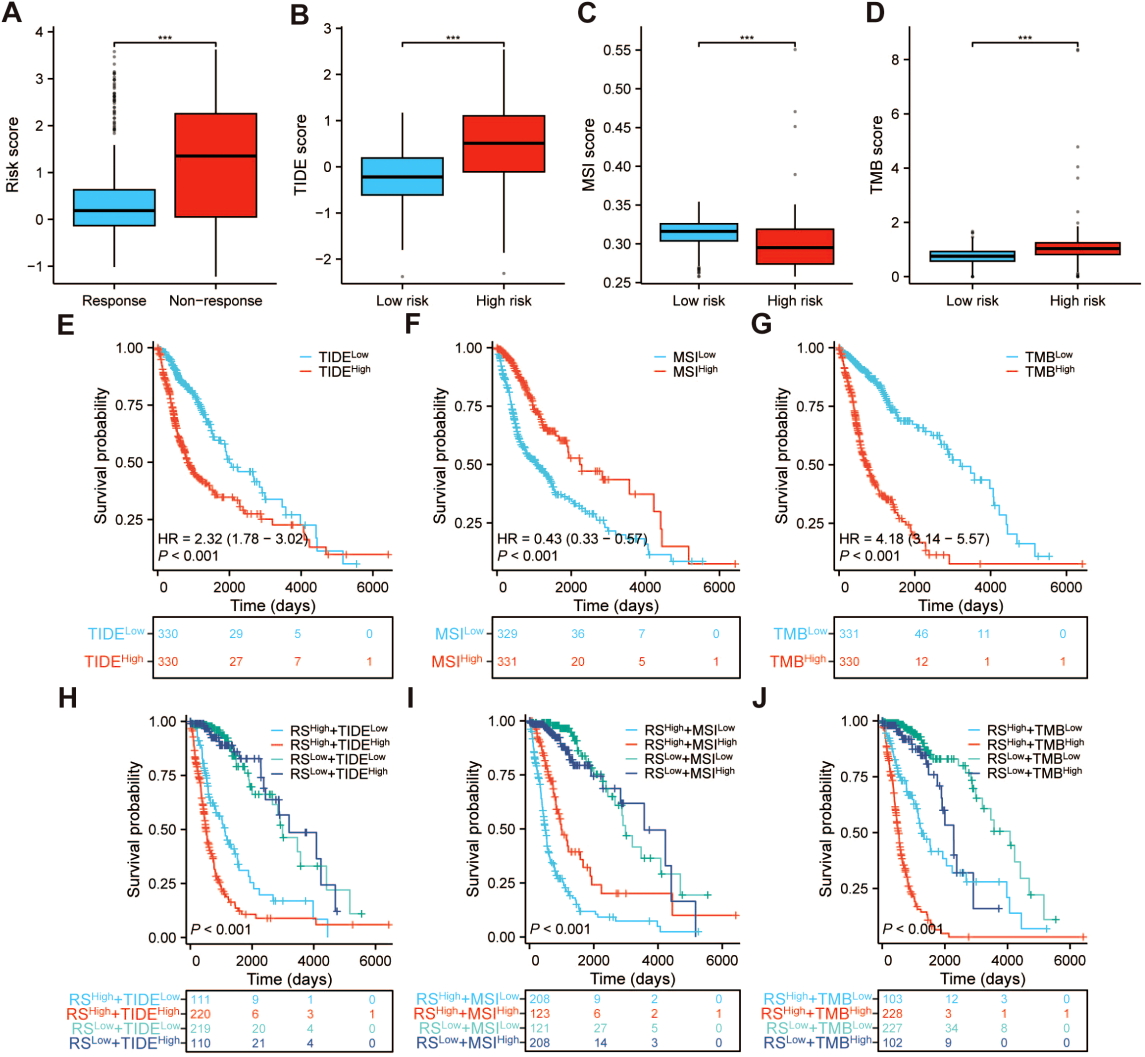
The benefit of the PRRS in immunotherapy. **(A)** Box plot showing that the PRRS of patients with immunotherapy-resistant glioma is higher than that of patients with immunotherapy-responsive glioma. Box plot of **(B)** TIDE, **(C)** MSI, and **(D)** TMB scores between two PRRS subgroups. Kaplan–Meier survival analysis comparing high- and low- **(E)** TIDE groups, **(F)** MSI groups, **(G)** TMB groups. Combined analysis of **(H)** TIDE or **(I)** MSI or **(J)** TMB and risk score, stratifying patients into four groups with significant differences in overall survival. ***p < 0.001.

To assess the potential of PRRS as a biomarker for predicting the clinical efficacy of immunotherapy, we compared the distribution of TIDE score, MSI score, and TMB score in different risk groups. According to our findings, glioma patients who showed a high efficacy of immunotherapy responses had significantly lower PRRS than those who were immunotherapy resistant (**Figure 12A**). The TIDE and TMB scores were higher in the high-risk group, whereas the MSI score was the opposite (**Figures 12B–D**). Furthermore, the Kaplan-Meier survival analysis revealed that patients with elevated TIDE scores exhibited decreased OS rates compared to those with lower TIDE scores (**Figure 12E**). However, patients with high MSI scores demonstrated enhanced OS rates in comparison to those with low MSI scores (**Figure 12F**). Conversely, patients with high TMB scores displayed diminished OS rates when compared to those with low TMB scores (**Figure 12G**). In addition, by integrating the TIDE score with PRRS, we stratified glioma patients into four distinct subgroups. Similarly, using the same methodology, we combined the MSI and TMB scores with PRRS, respectively, uncovering marked disparities in survival outcomes (**Figures 12H–J**). TIDE and PRRS appear to exert a joint and common influence on the survival outcomes of glioma patients, with TMB similarly sharing an effect with PRRS in this regard. These findings indicate that glioma patients with high PRRS may be more prone to cancer immune evasion. To delve into the role of PRRGs in the immune responses and mechanisms within TME, we evaluated the correlation between PRRGs and TMB as well as MSI. The results demonstrated a positive correlation between *APOC1*, *F2R*, *PTBP1*, *NCAPG*, and *IFI30* (*P<* 0.001) with TMB, whereas *BMP2* and *PDE2A* (*P<* 0.001) exhibited a negative correlation with TMB (**Supplementary Figure 14A**). On the contrary, *APOC1*, *F2R*, *PTBP1*, *NCAPG*, and *IFI30* (*P<* 0.001) were negatively correlated with MSI, while *BMP2* and *PDE2A* (*P<* 0.001) were positively correlated with MSI (**Supplementary Figure 14B**).

### Analysis of molecular docking and molecular dynamics simulations targeting PRGs

**Figure 13:**
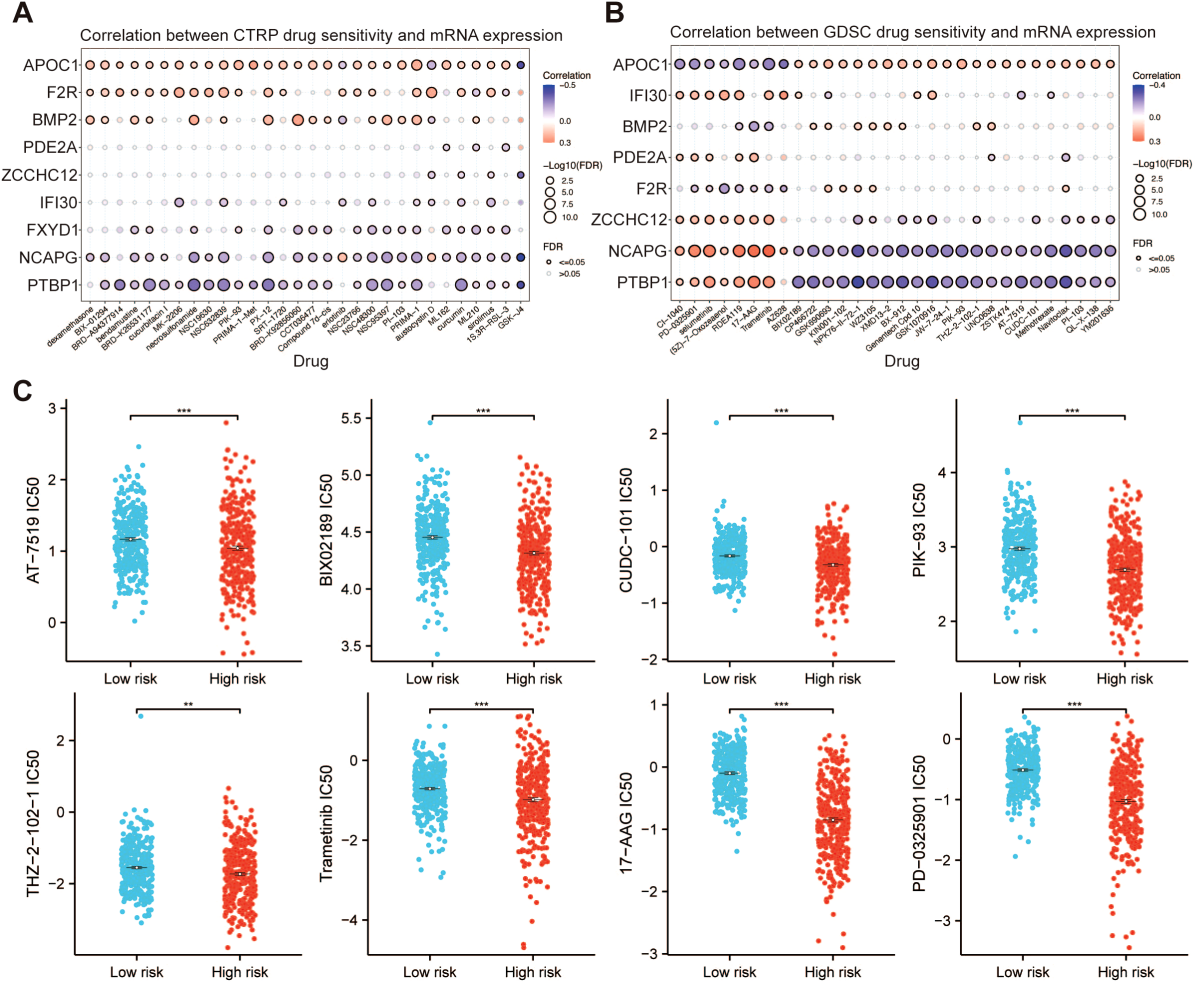
Drug sensitivity analysis. Antitumor medication predictions based on the expression of 9 PRGs in gliomas from the **(A)** CTRP and **(B)** GDSC datasets. **(C)** IC50 value distribution between high- and low-risk groups. **p < 0.01, ***p < 0.001.

**Figure 14:**
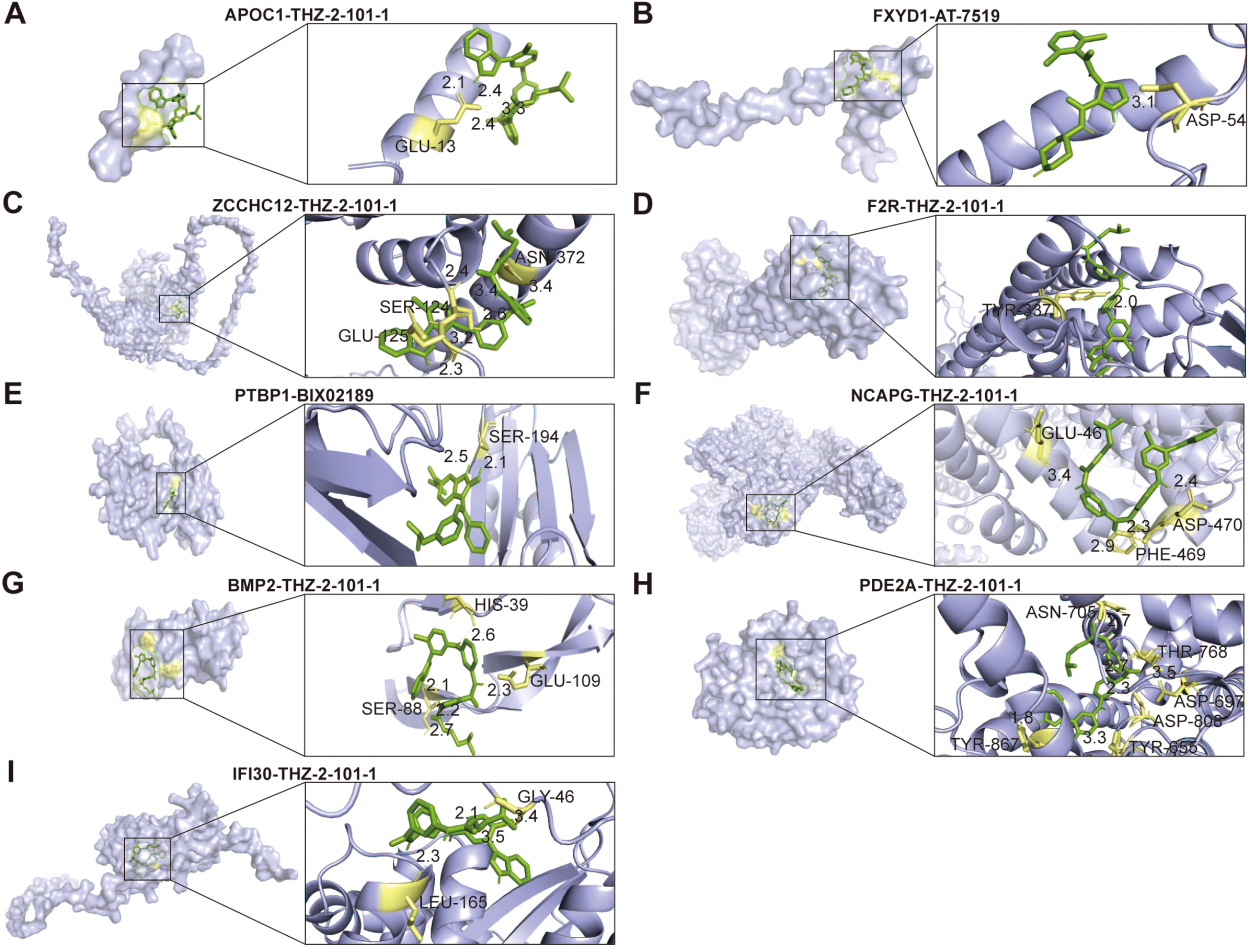
Molecular docking of the drug small molecules and **(A)** APOC1, **(B)** FXYD1, **(C)** ZCCHC12, **(D)** F2R, **(E)** PTBP1, **(F)** NCAPG, **(G)** BMP2, **(H)** PDE2A, **(I)** IFI30. The yellow dotted line and numbers indicate hydrogen bonding and hydrogen bond length, while green represents small molecule drugs.

To fully look into the potential worth of PRGs as new therapeutic targets, we first selected a number of drugs from the CTRP and GDSC datasets, which presented a significant association between drug sensitivity and the PRRS model (**Figures 13A, B**). In the high-risk group, the IC50 values of AT-7519, BIX02189, CUDC-101, PIK-93, THZ-2-102-1, Trametinib, 17-AAG, and PD-0325901 were significantly lower than those in the low-risk group, indicating that the sensitivity of glioma patients in the high-risk group to these drugs was higher than that in the low-risk group (**Figure 13C**). Subsequently, the lollipop plots demonstrated a statistically significant correlation between the IC50 values of these drugs and nine PRGs (**Supplementary Figure 15**). These findings suggested that these drugs could potentially serve as therapeutic options for glioma by modulating the expression of PRGs products. Utilizing both AutoDock and AutoDock Vina, we performed molecular docking between the proteins corresponding to the 9 PRGs and the drugs mentioned, with the results presented in **Supplementary Table 2**. We selected the best small molecules that bind to each protein and then used the PyMol process for visual analysis. The results indicated the following optimal docking binding energy: -5.4 kcal/mol for the binding energy between APOC1 and THZ-2-101-1, -6.1 kcal/mol for FXYD1 and AT-7519, -8.0 kcal/mol for ZCCHC12 and THZ-2-101-1, -11.0 kcal/mol for F2R and THZ-2-101-1, -8.4 kcal/mol for PTBP1 and BIX02189, -8.2 kcal/mol for NCAPG and THZ-2-101-1, -7.5 kcal/mol for BMP2 and THZ-2-101-1, -10.8 kcal/mol for PDE2A and THZ-2-101-1, and -7.3 kcal/mol for IFI30 and THZ-2-101-1 (**Figures 14A–I**).

The outcomes of MD simulations, encompassing RMSD and RMSF, constitute crucial evidence for assessing the stability of protein-ligand complexes. A lower RMSD value indicates a higher degree of stability (39). Moreover, the computation of RMSF offers deep insights into the fluctuations of protein residues throughout the simulation, influencing protein functionality, with elevated values signifying enhanced residue flexibility and diminished values indicating limited motion (40). We performed MD simulations on these 9 molecular docking systems and visualized their output data (**Supplementary Figure 16**). The RMSD results obtained from MD simulations revealed suboptimal overall performance for the NCAPG and THZ-2-101–1 complex system. Significant RMSD fluctuations suggested that the system failed to demonstrate effective inhibition of glioma (**Supplementary Figure 16F**). The RMSD of the FXYD1-AT-7519 complex exhibits initial fluctuations but stabilizes after 15 ns, whereas the RMSD of the PTBP1-BIX02189 complex stabilizes after 10 ns (**Supplementary Figures 16B, E**). It was also observed that APOC1, ZCCHC12, F2R, BMP2, PDE2A, and IFI30 could form stable complex molecular dynamics systems with THZ-2-101-1, respectively (**Supplementary Figures 16A, C, D, G-I**). **Supplementary Figures 16J-R** displays the fluctuations of residues pertaining to nine palmitoylation-associated proteins, respectively. These results suggested that APOC1, FXYD1, ZCCHC12, F2R, PTBP1, BMP2, PDE2A, and IFI30 might be new therapeutic targets for glioma.

### Functional verification of the anti-glioma effects of AT-7519, BIX02189, and THZ-2-101-1

**Figure 15:**
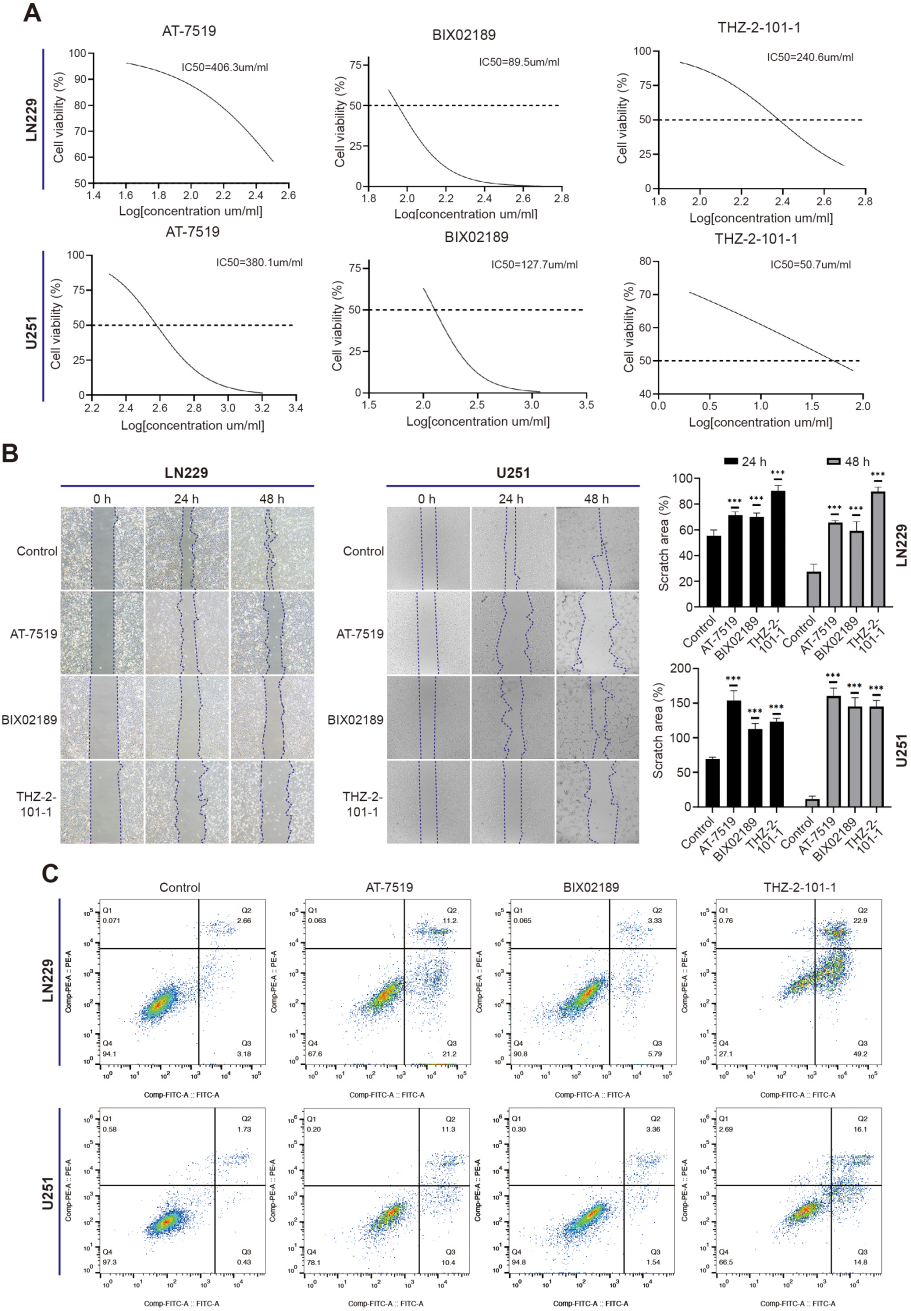
AT-7519, BIX02189, and THZ-2-101–1 are capable of inhibiting glioma cell migration and inducing apoptosis in these cells. **(A)** IC50 curves of AT-7519, BIX02189, and THZ-2-101–1 in LN229 and U251 cells were determined by CCK-8 assay following 48 hours of treatment. **(B)** Wound healing assays demonstrated impaired cell migration at 24 and 48 hours. **(C)** Flow cytometry analysis revealed a significant increase in the apoptosis rate of LN229 and U251 cells treated with AT-7519, BIX02189, and THZ-2-101–1 for 24 hours, compared to the control group. ***p < 0.001.

To validate the anti-glioma effects of the aforementioned compounds, we conducted a series of *in vitro* experiments employing LN229 and U251 glioma cells. Firstly, we employed the CCK-8 assay to assess the cytotoxicity. In LN229 cells, a concentration-dependent decline in cell viability was observed after 48 hours of treatment with AT-7519, BIX02189, and THZ-2-101-1, with IC50 values of 406.3, 89.5, and 240.6 μm/mL, respectively. Similarly, in U251 cells, the IC50 values for AT-7519, BIX02189, and THZ-2-101–1 were determined to be 380.1, 127.7, and 50.7 μm/mL, respectively (**Figure 15A**). Wound healing assays were employed to assess the effects of the three compounds on cell migration. Following 24 and 48 hours of treatment, a notable decrease in wound closure was evident in the drug-treated groups relative to the control, thereby underscoring the capacity of these compounds to suppress glioma cell motility (**Figure 15B**). Furthermore, following 24 hours of drug treatment, we employed flow cytometry to assess cell apoptosis. When compared to the cells in the control group, those treated with AT-7519, BIX02189, and THZ-2-101–1 demonstrated a notably elevated apoptotic index. (**Figure 15C**). Collectively, these findings confirm that AT-7519, BIX02189, and THZ-2-101–1 can inhibit the migration of glioma cells *in vitro* and also promote their apoptosis, thereby supporting their potential as effective anti-glioma agents.

## Discussion

Glioma, including LGG and GBM, is the most prevalent primary intracranial tumor, posing distinct challenges in treatment due to their aggressive nature and infiltrative growth patterns, which contribute to their high morbidity and mortality rates (41). Early diagnosis coupled with effective therapeutic interventions is conducive to the prognosis of glioma patients. There is therefore an urgent necessity to delve into the molecular mechanisms underlying glioma genesis and identify novel biomarkers, thereby facilitating the formulation of personalized treatment strategies and innovative therapies aimed at enhancing patient prognosis. The immune tumor microenvironment is crucial at every stage of glioma development, ranging from its onset to immune evasion, invasion, and relapse (42). In recent years, advancements in our comprehension of the immunopathological mechanisms and microenvironment of glioma have led to the emergence of immunotherapy targeting the glioma tumor immune microenvironment as a novel therapeutic option for glioma patients (43, 44). In addition to having fewer side effects and a low tumor recurrence rate, immunotherapy has the clinical benefit of inducing a long-lasting, effective, and systemic antitumor immune response (45).

Palmitoylation is a reversible post-translational lipid modification that can regulate immune cell infiltration and tumor immune landscape in the immune microenvironment (10, 46). It has been reported that glioma cells exhibit a heightened level of free fatty acids, which can potentiate post-translational modifications, notably palmitoylation (47). This palmitoylation process is instrumental in influencing oncogenic proteins and tumor suppressors, thereby playing an important role in metabolic and oncogenesis-promoting pathways (5, 48). Research has shown that palmitoylation does participate in regulating glioma cell survival, EMT, glycolysis, heterogeneity, tumorigenicity, chemotherapy resistance, and progression, as well as promoting ferroptosis resistance in glioma (11–14, 49–51). Hence, the accumulating evidence suggests that strategies aimed at modulating protein palmitoylation hold great promise for the development of novel and more effective therapeutic approaches for glioma patients.

This research systematically screened DEGs associated with palmitoylation between glioma and normal brain tissues. Utilizing an unsupervised clustering approach, we identified two distinct glioma clusters based on the expression profiles of PRGs. We observed that the prognosis of cluster 1 related to palmitoylation was significantly worse than that of cluster 2. Clinical feature analysis also showed that the WHO grade of glioma patients in cluster 1 was higher, which explained the lower survival rate of this cluster of patients. Given the intimate correlation between palmitoylation and the immune microenvironment, we conducted an investigation into the relationship between two palmitoylation subtypes and the infiltration patterns of tumor-associated immune cells, as well as the associated cytokines. Different immune cells have different roles in the TME. Tumor-associated macrophages (TAMs) encompass two functionally divergent subtypes. Specifically, M1 macrophages elicit an immune response by secreting pro-inflammatory cytokines, thereby exerting inhibitory effects on tumor growth and dissemination. Conversely, M2 macrophages, characterized by their secretion of anti-inflammatory cytokines, attenuate the immune surveillance and elimination functions of the body against tumors, manifesting properties that facilitate tumor progression, invasion, and metastasis (52). Similarly, tumor-associated neutrophils can undergo polarization into either an anti-tumor (N1) or a pro-tumor (N2) phenotype (53). CD8+ T lymphocytes elicit anti-tumor responses through the production of interferon-gamma (IFN-γ) (54). Conversely, Tregs can impede immune activation by expressing co-inhibitory molecules or secreting immunosuppressive cytokines (55). Tfh cells are characterized by their high expression of programmed death-1 (PD-1) and facilitating tumor immune responses (56). In our study, macrophages and Tregs that promote tumor immune suppression infiltrated more into cluster 1 than cluster 2. However, Tfh cells and activated NK cells can promote cancer immune infiltration into cluster 2 rather than cluster 1. Immune checkpoint genes, MHC, and T cell stimulators were also upregulated in cluster 1. An immunosuppressive milieu and tumor immune escape are more likely to occur when the immune checkpoint genes gene is highly expressed. Therefore, cluster 1 is associated with immunosuppression and leads to poor prognosis in glioma patients; on the other hand, cluster 2 is significantly associated with immune activation and leads to better prognosis. Additionally, cluster 2 responds better to immunotherapy, suggesting that it is an immune-favorable tumor.

A palmitoylation-related risk score comprising 9 prognostic markers found using LASSO Cox regression analysis was developed, taking into account the influence of PRGs and palmitoylation clusters on the clinical outcomes. Using the previously indicated formula, the PRRS was determined for every patient, and the median cutoff was used to stratify the high and low PRRS groups. In the TCGA-GBM LGG, CGGA_325, and CGGA_301 cohorts, the median OS of patients in the high PRRS subgroup was significantly shorter than that of patients in the low PRRS subgroup. Univariate and multivariate Cox regression analyses showed that PRRS was an independent prognostic factor. Of the genes identified by LASSO analysis, *APOC1* and *IFI30* expression levels in glioma were favorably correlated with the majority of immune cells, immunostimulators, immunoinhibitors, MHC molecules, chemokines, and chemokine receptors. Single-cell analysis results demonstrated a significant enrichment of *APOC1* and *IFI30* within monocytes/macrophages. Additionally, significant *PTBP1* enrichments were found in Tprolif cells, regulatory T cells, CD8+ T cells, and conventional CD4+ T cells. The expression of *APOC1* facilitates the proliferation, migration, and invasion of esophageal cancer cells and correlates with the characteristics of the immune microenvironment (57). *ZCCHC12* can promote the progression of osteosarcoma through the PI3K/AKT pathway, and ZCCHC12+ tumor cells in papillary thyroid cancer interact with CD36+ pro-inflammatory macrophages to promote tumor progression and recurrence (58). *PTBP1* and *NCAPG* play a role in some cancers, including colorectal cancer, renal cell carcinoma, breast cancer, and glioma, and can regulate tumorigenesis, invasion, and migration (59, 60). In contrast, single-cell analysis based on the CancerSEA database revealed that *PTBP1* and *NCAPG* are associated with various functions such as cell cycle, DNA damage, DNA repair, and proliferation, suggesting that they may be involved in the malignant progression of glioma. *IFI30* predominantly localizes within monocyte/macrophage populations and exhibits a strong correlation with the immune infiltration of glioma (61). Furthermore, LINC00265 stimulates the malignant progression of glioma by activating the expression of *IFI30* through the regulation of the transcription factor ZNF384 (62). In general, the genes that are part of the PRRS model have a major role in the development and TME of different types of cancer, ultimately impacting the proliferation, migration, invasion, and EMT of tumor cells. PRGs serve as potential biomarkers for the immune infiltration of glioma and may exert inhibitory or promotional effects on cancer-associated signaling pathways through the modulation of protein palmitoylation, thereby implying their potential as therapeutic targets in glioma treatment.

CNV can facilitate cancer progression through the upregulation of oncogene expression or the inactivation of tumor suppressor genes (63). We have observed heterozygous amplification of *IFI30* and *PTBP1*, suggesting that their CNV may have the potential to modify the immune infiltration status of glioma patients to a certain degree. TMB is recognized as a promising biomarker for immune responsiveness, and the accumulation of somatic mutations represents one of the primary causes underlying tumorigenesis, promoting the expression of neoantigens. Our analysis revealed a statistically significant disparity in survival duration between patients belonging to cluster 1, characterized by high TMB, and those in cluster 2, exhibiting low TMB. We found that the top two genes with higher mutation frequencies in cluster 1 were *IDH1* (54%) and *TP53* (45%), whereas the top two genes with higher mutation frequencies in cluster 2 were *IDH1* (79%) and *TP53* (35%). In glioma, over 80% of WHO grade II/III patients exhibit IDH mutations, while in WHO grade IV GBM, IDH mutations are prevalent in secondary GBM (73%) but rare in primary GBM (3.7%) (64, 65). A study demonstrated that the presence of IDH mutations in glioma predicts a good prognosis for patients (64). *TP53*, a common mutated gene in tumors, can function as a physiological inhibitor in the M2 macrophage polarization process by engaging the TP53/MDM2/c-MYC axis, with its mutation significantly associated with poor prognosis in various tumors (66, 67). This could account for the poorer prognosis observed in glioma patients with higher TMB in cluster 1. In addition, we found that the prognosis of glioma patients could be effectively differentiated according to PRRS and TMB. MSI is a potential driver of the progression and aggressive behavior of IDH-mutant astrocytomas, and combining PRRS and MSI can also effectively differentiate the prognosis of patients (68).

The frequent occurrence of CNV in cancer cells also increases tumor heterogeneity and can further regulate the biological behavior of tumor cells through epigenetic mechanisms such as DNA methylation (69, 70). High methylation can lead to gene expression silencing, while a decrease in promoter methylation levels is associated with higher gene expression (71). Our findings revealed a positive correlation between the expression levels of *APOC1* and *IFI30* in glioma and the majority of cytokines, while a negative correlation was observed between their methylation status and these cytokines. Furthermore, a reduced chance of survival in LGG was linked to high methylation of *APOC1*, *ZCCHC12*, *NCAPG*, *IFI30*, and *FXYD1*, but a higher risk of survival was linked to high methylation of *BMP2*. This is potentially attributed to the differential regulation of these genes’ expression by methylation at various loci within gliomas, ultimately exerting distinct influences on glioma prognosis.

DEGs between high and low PRRS subgroups were enriched in immune-related pathways such as complement and coagulation cascades. Therefore, differences in immune infiltration among risk groups were compared. In our research, both the immune score and stromal score were observed to be higher in the high-risk group, with a notable increase in M2 macrophage infiltration compared to the low-risk group. Moreover, the expression levels of ICIs, MHC molecules, and T-cell stimulatory factors were found to be higher in the high-risk group as compared to the low-risk group. The TIDE and TMB scores in the high-risk group are higher than those in the low-risk group, with such heightened risk scores implying an unfavorable prognosis for glioma patients. These findings indirectly imply that PRRS may play a key role in predicting the effectiveness of immunotherapy and that individuals with lower PRRS values may have a greater likelihood of deriving benefit from immunotherapeutic interventions. It is noteworthy that due to the existence of tumor heterogeneity, the blood-brain barrier, and mechanisms of drug resistance, the relatively poor sensitivity of glioma to various chemotherapy drugs results in limited therapeutic benefits derived from chemotherapy, possibly as a result of increased antitumor immune activity (72, 73). In our investigation, significant differences in the responses of different risk groups to chemotherapeutic agents were observed, suggesting that the developed PRRS model can serve as an adjunct in guiding the selection of chemotherapy drugs for glioma patients. In addition, molecular docking and molecular dynamics simulations were conducted on the screened small molecule compounds and 9 palmitoylation-related proteins. The results showed that these compounds may inhibit the proliferation and invasion of glioma through palmitoylation-related proteins. Finally, through *in vitro* drug experiments, we confirmed that AT-7519, BIX02189, and THZ-2-101–1 are capable of inhibiting glioma cell migration while simultaneously promoting apoptosis. These findings provide strong support for their potential as effective therapeutic agents against glioma.

The objective of this study was to categorize glioma patients into distinct palmitoylation-based clusters, identify DEGs among these clusters, develop a PRRS model, establish a connection between PRGs and glioma patient prognosis, and conduct validation from multiple angles and across various databases, revealing promising prospects for the PRRS model in predicting glioma patient prognosis. Of course, our study has several limitations as well. Given our retrospective analysis of public datasets such as TCGA, CGGA, and GEO, which is inevitably constrained by intratumoral or intrapatient tumor heterogeneity and affects our capacity to establish causal inferences, potentially compromising the applicability of the results in broader clinical practice, there is a pressing need for a prospective, multicenter study with a larger sample size to validate the accuracy of the established PRRS model. Furthermore, single-cell sequencing analysis has helped us understand the changes of PRGs in the glioma TME, it is imperative to conduct further functional and mechanistic investigations to elucidate the underlying mechanisms of PRGs in glioma, particularly focusing on their influence on the TME, and to validate the potential targets of action of the small molecules that have been screened for compounds. Finally, more studies and clinical trials are required to translate the results into clinical practice, particularly in various populations and WHO grades.

In summary, this study has not only identified palmitoylation-related genes in glioma patients but has also developed and validated a PRRS model for predicting overall survival in this patient cohort, exhibiting robust predictive performance. Furthermore, we have evaluated the differences in chemotherapeutic drug sensitivity and immunotherapy response among PRRS groups. Ultimately, the outcomes derived from molecular docking studies, molecular dynamics simulations, and *in vitro* drug experiments collectively suggest that AT-7519, BIX02189, and THZ-2-101–1 have the potential to become effective therapeutic drugs for glioma patients. These findings contribute to an advanced understanding of protein palmitoylation and offer novel strategies for personalized therapeutic interventions tailored to glioma patients.

## Data Availability

The original contributions presented in the study are included in the article/**Supplementary Material**. Further inquiries can be directed to the corresponding authors.

## References

[1] Ostrom QT Price M Neff C Cioffi G Waite KA Kruchko C . CBTRUS statistical report: primary brain and other central nervous system tumors diagnosed in the United States in 2016-2020. Neuro Oncol. (2023) 25:iv1–iv99. doi: 10.1093/neuonc/noad149, PMID: 37793125 PMC10550277

[2] Gusyatiner O Hegi ME . Glioma epigenetics: From subclassification to novel treatment options. Semin Cancer Biol. (2018) 51:50–8. doi: 10.1016/j.semcancer.2017.11.010, PMID: 29170066

[3] Louis DN Perry A Wesseling P Brat DJ Cree IA Figarella-Branger D . et al: The 2021 WHO Classification of Tumors of the Central Nervous System: a summary. Neuro Oncol. (2021) 23:1231–51. doi: 10.1093/neuonc/noab106, PMID: 34185076 PMC8328013

[4] Wang H Xu T Huang Q Jin W Chen J . Immunotherapy for Malignant glioma: current status and future directions. Trends Pharmacol Sci. (2020) 41:123–38. doi: 10.1016/j.tips.2019.12.003, PMID: 31973881

[5] Ko PJ Dixon SJ . Protein palmitoylation and cancer. EMBO Rep. (2018) 19. doi: 10.15252/embr.201846666, PMID: 30232163 PMC6172454

[6] Gao X Hannoush RN . A decade of click chemistry in protein palmitoylation: impact on discovery and new biology. Cell Chem Biol. (2018) 25:236–46. doi: 10.1016/j.chembiol.2017.12.002, PMID: 29290622

[7] Linder ME Deschenes RJ . Palmitoylation: policing protein stability and traffic. Nat Rev Mol Cell Biol. (2007) 8:74–84. doi: 10.1038/nrm2084, PMID: 17183362

[8] Sadeghi Rad H Monkman J Warkiani ME Ladwa R O’Byrne K Rezaei N . Understanding the tumor microenvironment for effective immunotherapy. Med Res Rev. (2021) 41:1474–98. doi: 10.1002/med.21765, PMID: 33277742 PMC8247330

[9] Elguindy MM Young JS Ho WS Lu RO . Co-evolution of glioma and immune microenvironment. J Immunother Cancer. (2024) 12. doi: 10.1136/jitc-2024-009175, PMID: 39631850 PMC11624716

[10] Chen X Hu L Yang H Ma H Ye K Zhao C . DHHC protein family targets different subsets of glioma stem cells in specific niches. J Exp Clin Cancer Res. (2019) 38:25. doi: 10.1186/s13046-019-1033-2, PMID: 30658672 PMC6339410

[11] Chen X Ma H Wang Z Zhang S Yang H Fang Z . EZH2 palmitoylation mediated by ZDHHC5 in p53-mutant glioma drives Malignant development and progression. Cancer Res. (2017) 77:4998–5010. doi: 10.1158/0008-5472.CAN-17-1139, PMID: 28775165

[12] Wang Y Shen N Yang Y Xia Y Zhang W Lu Y . ZDHHC5-mediated S-palmitoylation of FAK promotes its membrane localization and epithelial-mesenchymal transition in glioma. Cell Commun Signaling. (2024) 22:46. doi: 10.1186/s12964-023-01366-z, PMID: 38233791 PMC10795333

[13] Zhao C Yu H Fan X Niu W Fan J Sun S . GSK3β palmitoylation mediated by ZDHHC4 promotes tumorigenicity of glioblastoma stem cells in temozolomide-resistant glioblastoma through the EZH2–STAT3 axis. Oncogenesis. (2022) 11:28. doi: 10.1038/s41389-022-00402-w, PMID: 35606353 PMC9126914

[14] Wang Z Wang Y Shen N Liu Y Xu X Zhu R . AMPKα1-mediated ZDHHC8 phosphorylation promotes the palmitoylation of SLC7A11 to facilitate ferroptosis resistance in glioblastoma. Cancer Lett. (2024) 584:216619. doi: 10.1016/j.canlet.2024.216619, PMID: 38211651

[15] Ritchie ME Phipson B Wu D Hu Y Law CW Shi W . limma powers differential expression analyses for RNA-sequencing and microarray studies. Nucleic Acids Res. (2015) 43:e47. doi: 10.1093/nar/gkv007, PMID: 25605792 PMC4402510

[16] Zhang M Sun L Ru Y Zhang S Miao J Guo P . A risk score system based on DNA methylation levels and a nomogram survival model for lung squamous cell carcinoma. Int J Mol Med. (2020) 46:252–64. doi: 10.3892/ijmm.2020.4590, PMID: 32377703 PMC7255475

[17] Warde-Farley D Donaldson SL Comes O Zuberi K Badrawi R Chao P . et al: The GeneMANIA prediction server: biological network integration for gene prioritization and predicting gene function. Nucleic Acids Res. (2010) 38:W214–20. doi: 10.1093/nar/gkq537, PMID: 20576703 PMC2896186

[18] Yu G Wang LG Han Y He QY . clusterProfiler: an R package for comparing biological themes among gene clusters. Omics. (2012) 16:284–7. doi: 10.1089/omi.2011.0118, PMID: 22455463 PMC3339379

[19] Subramanian A Tamayo P Mootha VK Mukherjee S Ebert BL Gillette MA . Gene set enrichment analysis: a knowledge-based approach for interpreting genome-wide expression profiles. Proc Natl Acad Sci U S A. (2005) 102:15545–50. doi: 10.1073/pnas.0506580102, PMID: 16199517 PMC1239896

[20] Wilkerson MD Hayes DN . ConsensusClusterPlus: a class discovery tool with confidence assessments and item tracking. Bioinformatics. (2010) 26:1572–3. doi: 10.1093/bioinformatics/btq170, PMID: 20427518 PMC2881355

[21] Newman AM Liu CL Green MR Gentles AJ Feng W Xu Y . Robust enumeration of cell subsets from tissue expression profiles. Nat Methods. (2015) 12:453–7. doi: 10.1038/nmeth.3337, PMID: 25822800 PMC4739640

[22] Jiang P Gu S Pan D Fu J Sahu A Hu X . et al: Signatures of T cell dysfunction and exclusion predict cancer immunotherapy response. Nat Med. (2018) 24:1550–8. doi: 10.1038/s41591-018-0136-1, PMID: 30127393 PMC6487502

[23] Mayakonda A Lin DC Assenov Y Plass C Koeffler HP . Maftools: efficient and comprehensive analysis of somatic variants in cancer. Genome Res. (2018) 28:1747–56. doi: 10.1101/gr.239244.118, PMID: 30341162 PMC6211645

[24] Simon N Friedman J Hastie T Tibshirani R . Regularization paths for cox’s proportional hazards model via coordinate descent. J Stat Softw. (2011) 39:1–13. doi: 10.18637/jss.v039.i05, PMID: 27065756 PMC4824408

[25] Iasonos A Schrag D Raj GV Panageas KS . How to build and interpret a nomogram for cancer prognosis. J Clin Oncol. (2008) 26:1364–70. doi: 10.1200/JCO.2007.12.9791, PMID: 18323559

[26] Li T Fan J Wang B Traugh N Chen Q Liu JS . TIMER: A web server for comprehensive analysis of tumor-infiltrating immune cells. Cancer Res. (2017) 77:e108–10. doi: 10.1158/1538-7445.AM2017-108 PMC6042652 29092952

[27] Hänzelmann S Castelo R Guinney J . GSVA: gene set variation analysis for microarray and RNA-seq data. BMC Bioinf. (2013) 14:7. doi: 10.1186/1471-2105-14-7, PMID: 23323831 PMC3618321

[28] Han Y Wang Y Dong X Sun D Liu Z Yue J . TISCH2: expanded datasets and new tools for single-cell transcriptome analyses of the tumor microenvironment. Nucleic Acids Res. (2023) 51:D1425–d1431. doi: 10.1093/nar/gkac959, PMID: 36321662 PMC9825603

[29] Cerami E Gao J Dogrusoz U Gross BE Sumer SO Aksoy BA . et al: The cBio cancer genomics portal: an open platform for exploring multidimensional cancer genomics data. Cancer Discov. (2012) 2:401–4. doi: 10.1158/2159-8290.CD-12-0095, PMID: 22588877 PMC3956037

[30] Collier TA Piggot TJ Allison JR . Molecular dynamics simulation of proteins. Methods Mol Biol. (2020) 2073:311–27. doi: 10.1007/978-1-4939-9869-2_17, PMID: 31612449

[31] Collier TA Piggot TJ Allison JR . Molecular dynamics simulation of proteins. In: Protein Nanotechnology: Protocols, Instrumentation, and Applications (2020). New York: Springer p. 311–27. 10.1007/978-1-4939-9869-2_17 31612449

[32] Zhang D Wang Z Li J Zhu J . Exploring the possible molecular targeting mechanism of Saussurea involucrata in the treatment of COVID-19 based on bioinformatics and network pharmacology. Comput Biol Med. (2022) 146:105549. doi: 10.1016/j.compbiomed.2022.105549, PMID: 35751193 PMC9035664

[33] Balasubramanian A Hsu AY Ghimire L Tahir M Devant P Fontana P . et al: The palmitoylation of gasdermin D directs its membrane translocation and pore formation during pyroptosis. Sci Immunol. (2024) 9:eadn1452. doi: 10.1126/sciimmunol.adn1452, PMID: 38530158 PMC11367861

[34] Chan TA Wolchok JD Snyder A . Genetic basis for clinical response to CTLA-4 blockade in melanoma. N Engl J Med. (2015) 373:1984. doi: 10.1056/NEJMc1508163, PMID: 26559592

[35] Tang B Zhu J Zhao Z Lu C Liu S Fang S . et al: Diagnosis and prognosis models for hepatocellular carcinoma patient’s management based on tumor mutation burden. J Adv Res. (2021) 33:153–65. doi: 10.1016/j.jare.2021.01.018, PMID: 34603786 PMC8463909

[36] Gregson J Sharples L Stone GW Burman CF Öhrn F Pocock S . Nonproportional hazards for time-to-event outcomes in clinical trials: JACC review topic of the week. J Am Coll Cardiol. (2019) 74:2102–12. doi: 10.1016/j.jacc.2019.08.1034, PMID: 31623769

[37] Zhang Y Zhang Z . The history and advances in cancer immunotherapy: understanding the characteristics of tumor-infiltrating immune cells and their therapeutic implications. Cell Mol Immunol. (2020) 17:807–21. doi: 10.1038/s41423-020-0488-6, PMID: 32612154 PMC7395159

[38] Jiang X Chen X Huang X Wang C Wang C Pan C . DNA methylation-regulated YTHDF2 correlates with cell migration and immune cell infiltration in glioma. Aging (Albany NY). (2022) 14:7774–93. doi: 10.18632/aging.204104, PMID: 35661004 PMC9596213

[39] Zaheen A Rajkhowa S Al-Hussain SA Zaki MEA . Integrated computational strategies for Polypharmacological profiling and identification of anti-inflammatory targets in Rungia pectinata L. J Cell Mol Med. (2024) 28:e70158. doi: 10.1111/jcmm.70158, PMID: 39629503 PMC11615512

[40] Bora N Nath Jha A . An integrative approach using systems biology, mutational analysis with molecular dynamics simulation to challenge the functionality of a target protein. Chem Biol Drug Des. (2019) 93:1050–60. doi: 10.1111/cbdd.13502, PMID: 30891955

[41] Van Meir EG Hadjipanayis CG Norden AD Shu HK Wen PY Olson JJ . Exciting new advances in neuro-oncology: the avenue to a cure for Malignant glioma. CA Cancer J Clin. (2010) 60:166–93. doi: 10.3322/caac.20069, PMID: 20445000 PMC2888474

[42] Wen J Liu D Zhu H Shu K . Microenvironmental regulation of tumor-associated neutrophils in Malignant glioma: from mechanism to therapy. J Neuroinflamm. (2024) 21:226. doi: 10.1186/s12974-024-03222-4, PMID: 39285276 PMC11406851

[43] Look T Sankowski R Bouzereau M Fazio S Sun M Buck A . CAR T cells, CAR NK cells, and CAR macrophages exhibit distinct traits in glioma models but are similarly enhanced when combined with cytokines. Cell Rep Med. (2025) 2025:101931. doi: 10.1016/j.xcrm.2025.101931, PMID: 39889712 PMC11866521

[44] Sui X Ji J Zhang H . CTCs detection methods *in vivo* and *in vitro* and their application in tumor immunotherapy. J Surg Oncol. (2025) 132:80–7. doi: 10.1002/jso.28102, PMID: 39878404

[45] Khalil DN Smith EL Brentjens RJ Wolchok JD . The future of cancer treatment: immunomodulation, CARs and combination immunotherapy. Nat Rev Clin Oncol. (2016) 13:273–90. doi: 10.1038/nrclinonc.2016.25, PMID: 26977780 PMC5551685

[46] Tang F Yang C Li FP Yu DH Pan ZY Wang ZF . Palmitoyl transferases act as potential regulators of tumor-infiltrating immune cells and glioma progression. Mol Ther Nucleic Acids. (2022) 28:716–31. doi: 10.1016/j.omtn.2022.04.030, PMID: 35664705 PMC9126852

[47] Tang F Liu Z Chen X Yang J Wang Z Li Z . Current knowledge of protein palmitoylation in gliomas. Mol Biol Rep. (2022) 49:10949–59. doi: 10.1007/s11033-022-07809-z, PMID: 36044113

[48] Chen S Zhu B Yin C Liu W Han C Chen B . et al: Palmitoylation-dependent activation of MC1R prevents melanomagenesis. Nature. (2017) 549:399–403. doi: 10.1038/nature23887, PMID: 28869973 PMC5902815

[49] Chen X Li H Fan X Zhao C Ye K Zhao Z . Protein palmitoylation regulates cell survival by modulating XBP1 activity in glioblastoma multiforme. Mol Ther Oncolyt. (2020) 17:518–30. doi: 10.1016/j.omto.2020.05.007, PMID: 33024813 PMC7525067

[50] Fan X Fan J Yang H Zhao C Niu W Fang Z . Heterogeneity of subsets in glioblastoma mediated by Smad3 palmitoylation. Oncogenesis. (2021) 10:72. doi: 10.1038/s41389-021-00361-8, PMID: 34707087 PMC8551152

[51] Zhang Z Li X Yang F Chen C Liu P Ren Y . DHHC9-mediated GLUT1 S-palmitoylation promotes glioblastoma glycolysis and tumorigenesis. Nat Commun. (2021) 12:5872. doi: 10.1038/s41467-021-26180-4, PMID: 34620861 PMC8497546

[52] Shao N Qiu H Liu J Xiao D Zhao J Chen C . Targeting lipid metabolism of macrophages: A new strategy for tumor therapy. J Adv Res. (2025) 68:99–114. doi: 10.1016/j.jare.2024.02.009, PMID: 38373649 PMC11785569

[53] Fridlender ZG Sun J Kim S Kapoor V Cheng G Ling L . Polarization of tumor-associated neutrophil phenotype by TGF-beta: “N1” versus “N2” TAN. Cancer Cell. (2009) 16:183–94. doi: 10.1016/j.ccr.2009.06.017, PMID: 19732719 PMC2754404

[54] St Paul M Ohashi PS . The roles of CD8(+) T cell subsets in antitumor immunity. Trends Cell Biol. (2020) 30:695–704. doi: 10.1016/j.tcb.2020.06.003, PMID: 32624246

[55] Granito A Muratori L Lalanne C Quarneti C Ferri S Guidi M . Hepatocellular carcinoma in viral and autoimmune liver diseases: Role of CD4+ CD25+ Foxp3+ regulatory T cells in the immune microenvironment. World J Gastroenterol. (2021) 27:2994–3009. doi: 10.3748/wjg.v27.i22.2994, PMID: 34168403 PMC8192285

[56] Jogdand GM Mohanty S Devadas S . Regulators of tfh cell differentiation. Front Immunol. (2016) 7:520. doi: 10.3389/fimmu.2016.00520, PMID: 27933060 PMC5120123

[57] Guo Q Liu XL Jiang N Zhang WJ Guo SW Yang H . Decreased APOC1 expression inhibited cancer progression and was associated with better prognosis and immune microenvironment in esophageal cancer. Am J Cancer Res. (2022) 12:4904–29., PMID: 36504892 PMC9729889

[58] Zhang X Guo L Tian W Yang Y Yin Y Qiu Y . et al: CD36+ Proinflammatory macrophages interact with ZCCHC12+ Tumor cells in papillary thyroid cancer promoting tumor progression and recurrence. Cancer Immunol Res. (2024) 12:1621–39. doi: 10.1158/2326-6066.CIR-23-1047, PMID: 39178310

[59] Zhu W Zhou BL Rong LJ Ye L Xu HJ Zhou Y . et al: Roles of PTBP1 in alternative splicing, glycolysis, and oncogensis. J Zhejiang Univ Sci B. (2020) 21:122–36. doi: 10.1631/jzus.B1900422, PMID: 32115910 PMC7076342

[60] Cai X Gao J Shi C Guo WZ Guo D Zhang S . The role of NCAPG in various of tumors. BioMed Pharmacother. (2022) 155:113635. doi: 10.1016/j.biopha.2022.113635, PMID: 36095957

[61] Jiang L Wang P Hou Y Chen J Li H . Comprehensive single-cell pan-cancer atlas unveils IFI30+ macrophages as key modulators of intra-tumoral immune dynamics. Front Immunol. (2025) 16:1523854. doi: 10.3389/fimmu.2025.1523854, PMID: 39925804 PMC11802554

[62] Yang J Yang S Cai J Chen H Sun L Wang J . A transcription factor ZNF384, regulated by LINC00265, activates the expression of IFI30 to stimulate Malignant progression in glioma. ACS Chem Neurosci. (2024) 15:290–9. doi: 10.1021/acschemneuro.3c00562, PMID: 38141017

[63] Xiao J Jin X Zhang C Zou H Chang Z Han N . Systematic analysis of enhancer regulatory circuit perturbation driven by copy number variations in Malignant glioma. Theranostics. (2021) 11:3060–73. doi: 10.7150/thno.54150, PMID: 33537074 PMC7847679

[64] Yan H Parsons DW Jin G McLendon R Rasheed BA Yuan W . et al: IDH1 and IDH2 mutations in gliomas. N Engl J Med. (2009) 360:765–73. doi: 10.1056/NEJMoa0808710, PMID: 19228619 PMC2820383

[65] Nobusawa S Watanabe T Kleihues P Ohgaki H . IDH1 mutations as molecular signature and predictive factor of secondary glioblastomas. Clin Cancer Res. (2009) 15:6002–7. doi: 10.1158/1078-0432.CCR-09-0715, PMID: 19755387

[66] Hafner A Bulyk ML Jambhekar A Lahav G . The multiple mechanisms that regulate p53 activity and cell fate. Nat Rev Mol Cell Biol. (2019) 20:199–210. doi: 10.1038/s41580-019-0110-x, PMID: 30824861

[67] Kandoth C McLellan MD Vandin F Ye K Niu B Lu C . et al: Mutational landscape and significance across 12 major cancer types. Nature. (2013) 502:333–9. doi: 10.1038/nature12634, PMID: 24132290 PMC3927368

[68] Richardson TE Walker JM Hambardzumyan D Brem S Hatanpaa KJ Viapiano MS . et al: Genetic and epigenetic instability as an underlying driver of progression and aggressive behavior in IDH-mutant astrocytoma. Acta Neuropathol. (2024) 148:5. doi: 10.1007/s00401-024-02761-7, PMID: 39012509 PMC11252228

[69] Rooney K Sadikovic B . DNA methylation episignatures in neurodevelopmental disorders associated with large structural copy number variants: clinical implications. Int J Mol Sci. (2022) 23. doi: 10.3390/ijms23147862, PMID: 35887210 PMC9324454

[70] El Khoury LY Pan X Hlady RA Wagner RT Shaikh S Wang L . Extensive intratumor regional epigenetic heterogeneity in clear cell renal cell carcinoma targets kidney enhancers and is associated with poor outcome. Clin Epigenet. (2023) 15:71. doi: 10.1186/s13148-023-01471-3, PMID: 37120552 PMC10149001

[71] Long S Yan Y Xu H Wang L Jiang J Xu Z . et al: Insights into the regulatory role of RNA methylation modifications in glioma. J Transl Med. (2023) 21:810. doi: 10.1186/s12967-023-04653-y, PMID: 37964279 PMC10644640

[72] Maity S Bhuyan T Jewell C Kawakita S Sharma S Nguyen HT . et al: recent developments in glioblastoma-on-A-chip for advanced drug screening applications. Small. (2025) 21:e2405511. doi: 10.1002/smll.202405511, PMID: 39535474 PMC11719323

[73] Chen T Ma W Wang X Ye Q Hou X Wang Y . Insights of immune cell heterogeneity, tumor-initiated subtype transformation, drug resistance, treatment and detecting technologies in glioma microenvironment. J Adv Res. (2024) 73:527–54. doi: 10.1016/j.jare.2024.07.033, PMID: 39097088 PMC12147621

