# Supplementary material for: Comprehensive analysis of palmitoylation-related proteins for prognostic risk evaluation and tumor immune microenvironment assessment in glioma
Source: Front Immunol. 2025 Dec 8;16:1599769. doi: 10.3389/fimmu.2025.1599769 (PMC12719430; doi:10.3389/fimmu.2025.1599769)
Supplement: Supplementary file 1 [file DataSheet1.docx]

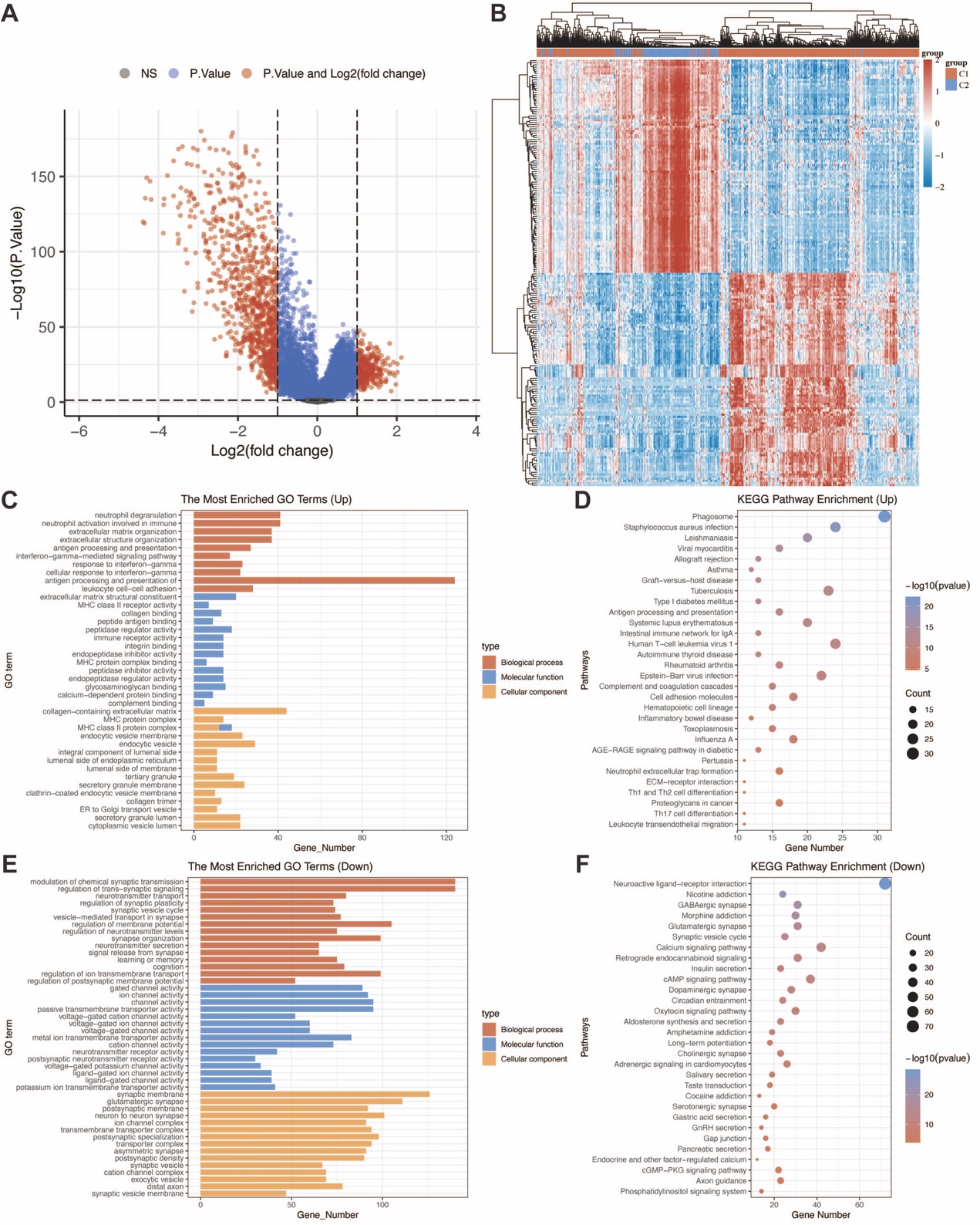


**Supplementary Figure 1.** Identification of DEGs between two clusters and functional enrichment analysis. (**A**) Exhibition of DEGs in theTCGA-GBMLGG dataset between two clusters using volcano plots. (**B**) The heatmap displaying the expression of DEGs. Functional enrichment analysis includes the (**C**) GO term enrichment results and (**D**) KEGG pathway enrichment results for differentially upregulated genes, as well as the (**E**) GO term enrichment results and (**F**) KEGG pathway enrichment results for differentially downregulated genes.


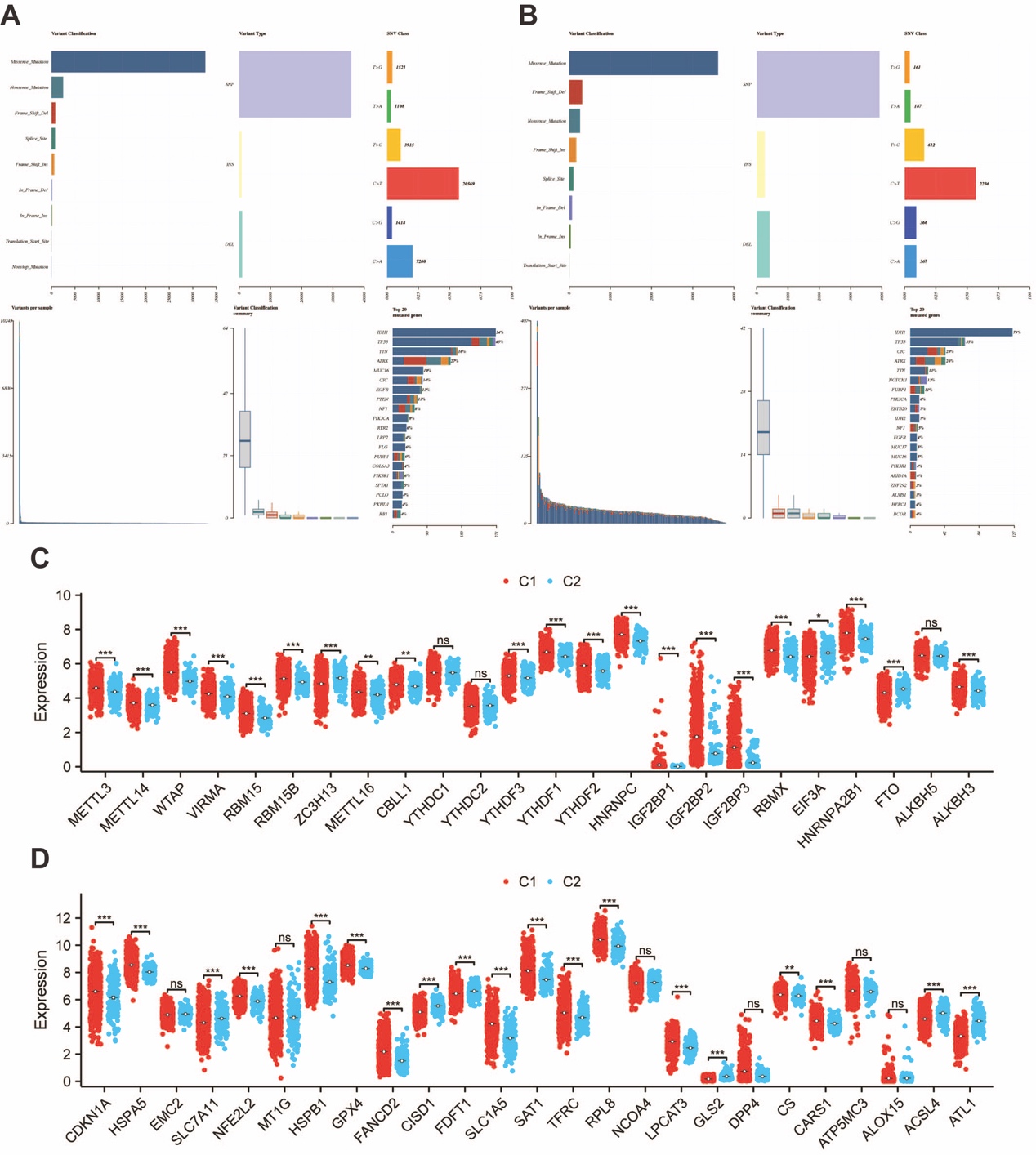


**Supplementary Figure 2.** (**A-B**) Cohort summary plot depicting the distribution of variants based on their classification, type, and single-nucleotide variants (SNV) class. The lower section (proceeding from left to right) signifies the mutation burden for each sample and the type of variant classification. Additionally, a stacked bar chart illustrates the top 20 most mutated genes. Difference of (**C**) m6A-related genes and (**D**) ferroptosis-related genes between different clusters based on the TCGA-GBMLGG dataset. * *p* < 0.05, ** *p* < 0.01, *** *p* < 0.001, NS, not significant.


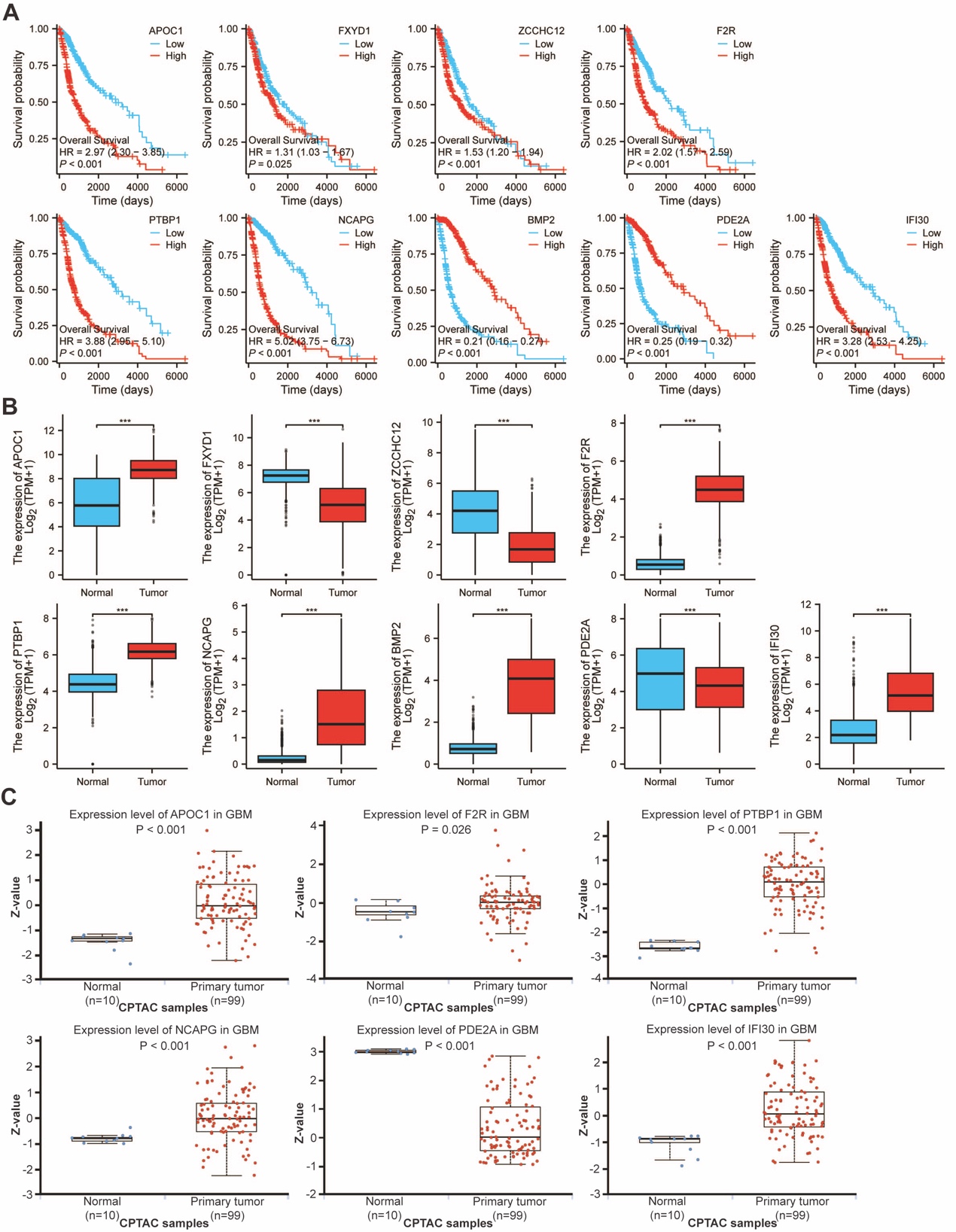


**Supplementary Figure 3.** Kaplan–Meier survival and differential expression analysis of 9 PRGs. (**A**) A Kaplan–Meier survival analysis of the eight genes, showing significant associations with overall survival in the TCGA-GBMLGG cohort. (**B**) Comparison of mRNA expression levels of 9 PRGs in glioma. (**C**) Comparison of protein expression levels of PRGs in GBM. *** *p* < 0.001.


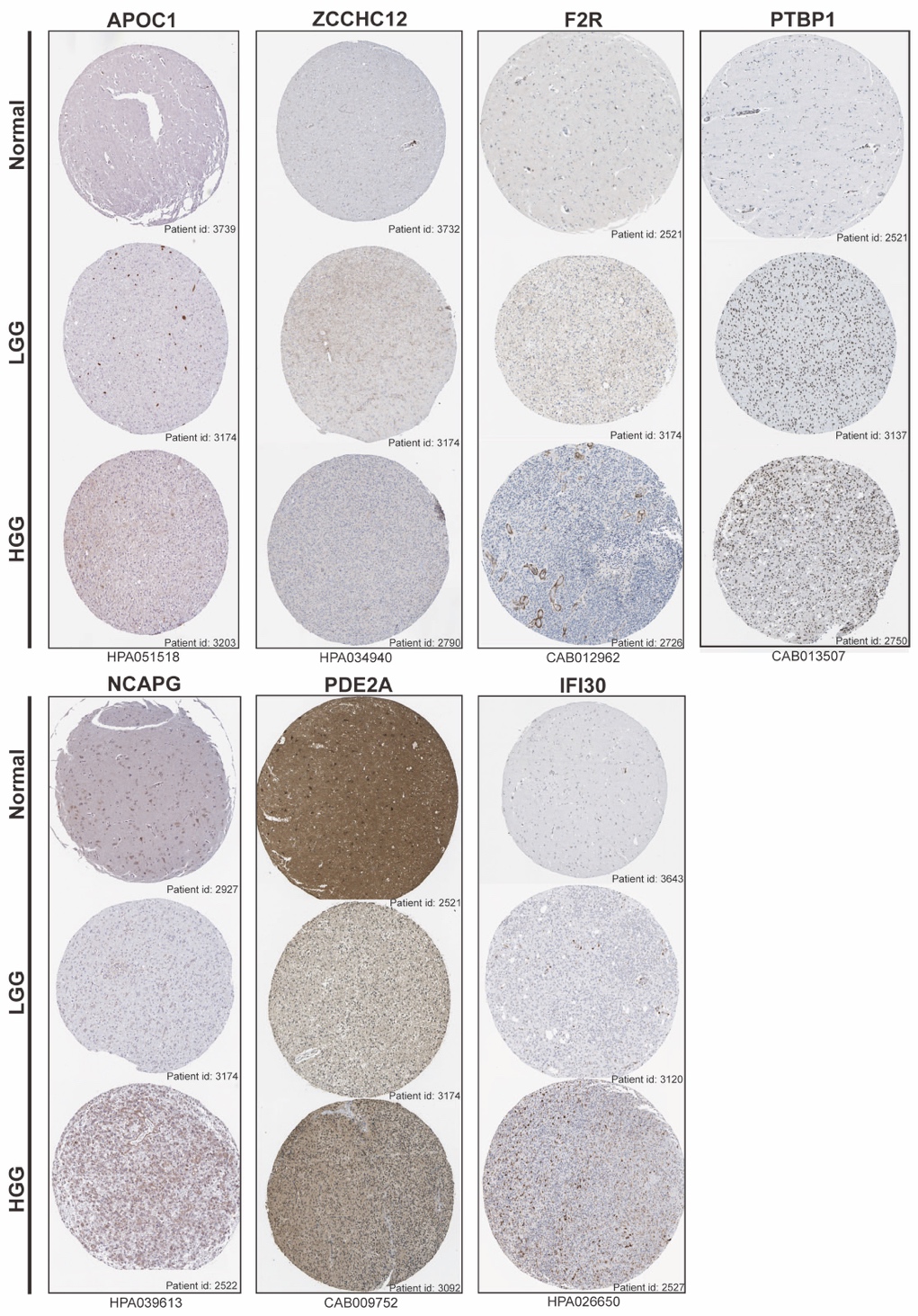


**Supplementary Figure 4.** Immunohistochemistry revealed differential expression of PRGs-corresponding proteins in normal brain tissue, lower grade glioma (LGG), and high-grade glioma (HGG).


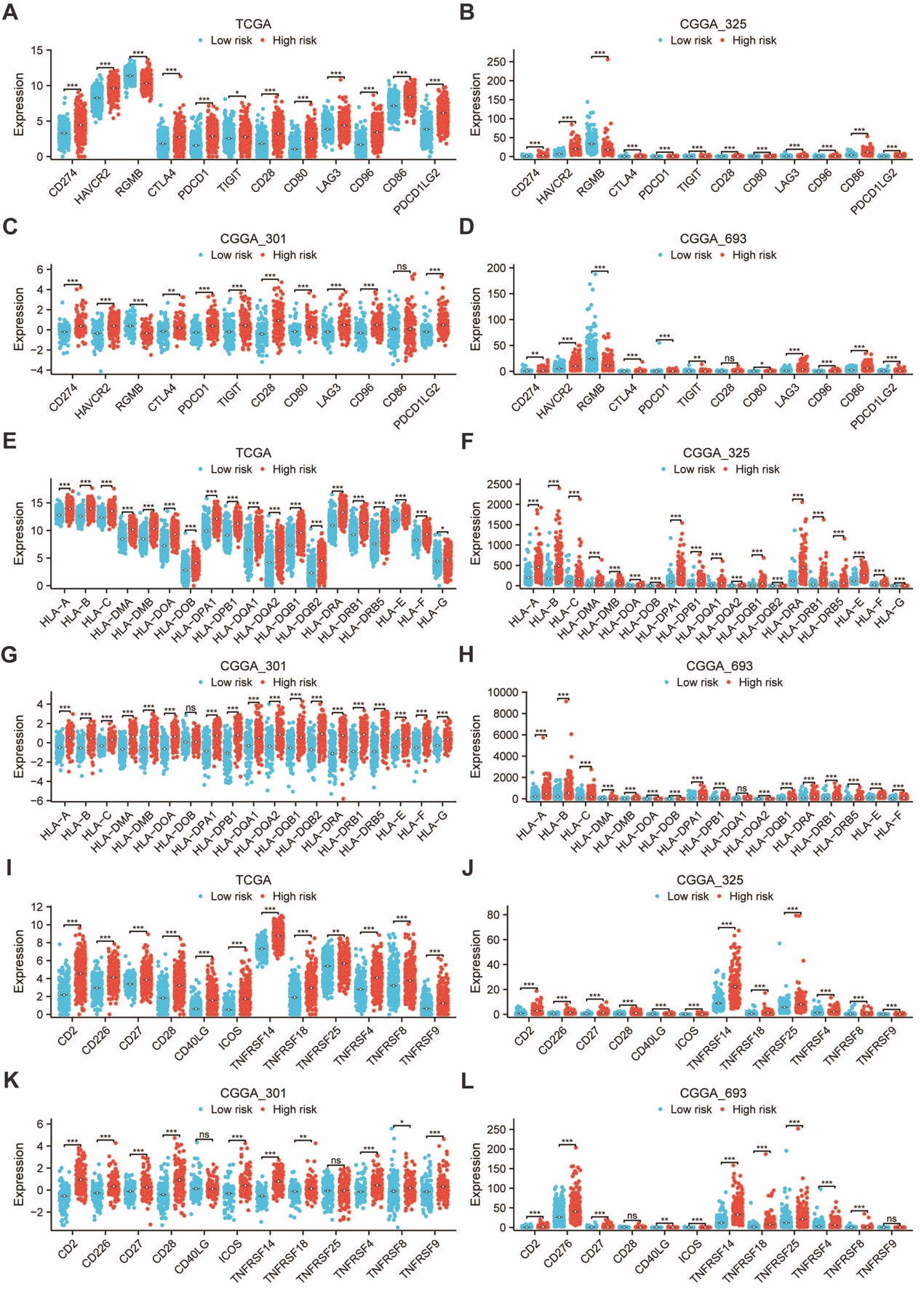


**Supplementary Figure 5.** Differential expression analysis of immune checkpoint genes between high- and low-risk groups based on the (**A**) TCGA-GBMLGG, (**B**) CGGA_325, (**C**) CGGA_301, and (**D**) CGGA_693 cohorts. Differential expression analysis of MHC between high- and low-risk groups based on the (**E**) TCGA-GBMLGG, (**F**) CGGA_325, (**G**) CGGA_301, and (**H**) CGGA_693 cohorts. Differential expression analysis of T-cell stimulators between high- and low-risk groups based on the (**I**) TCGA-GBMLGG, (**J**) CGGA_325, (**K**) CGGA_301, and (**L**) CGGA_693 cohorts. * *p* < 0.05, ** *p* < 0.01, *** *p* < 0.001, NS, not significant.


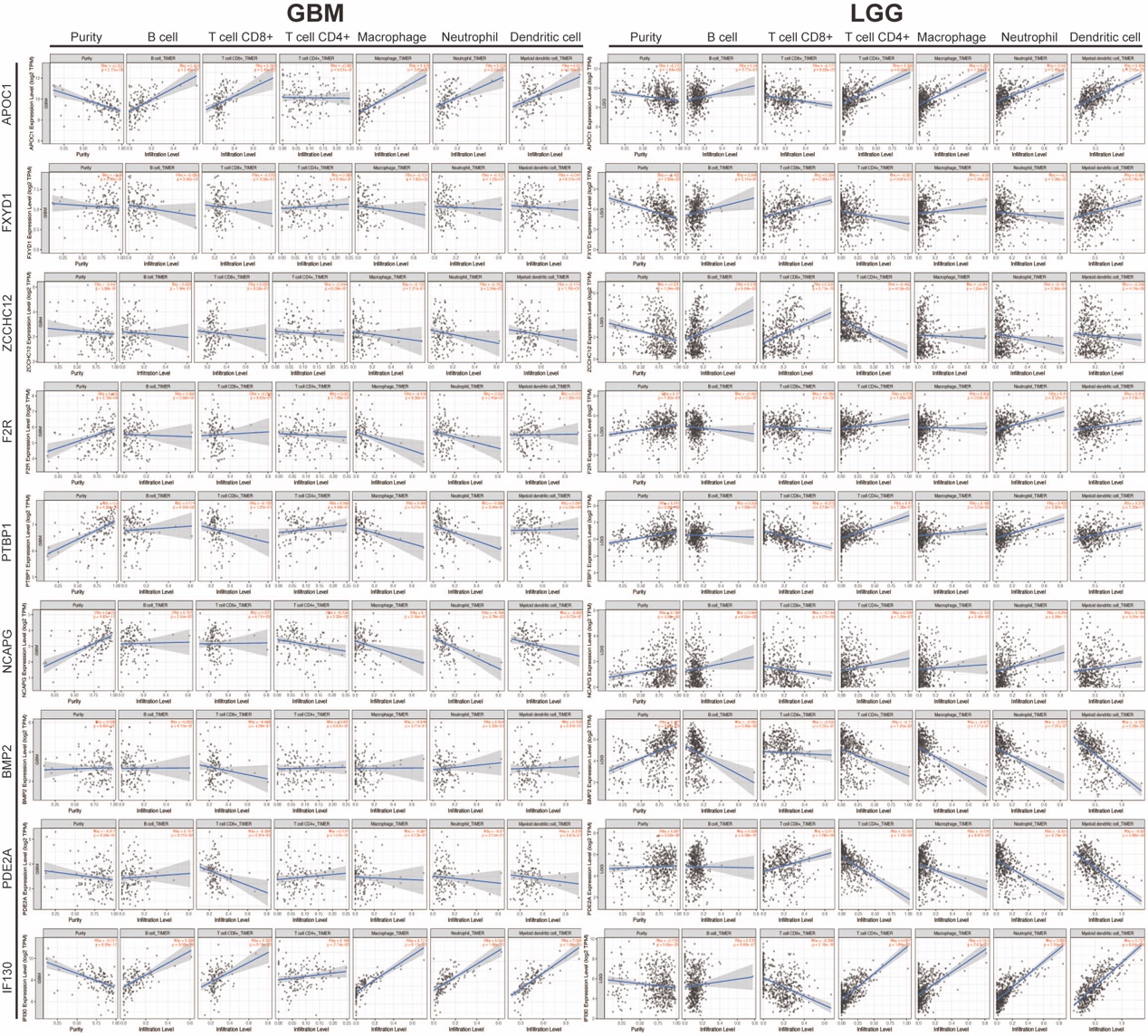


**Supplementary Figure 6.** Correlations of 9 PRGs expression levels with infiltration rates of B cells, CD8+ T cells, CD4+ T cells, macrophages, neutrophils, and dendritic cells in GBM and LGG.


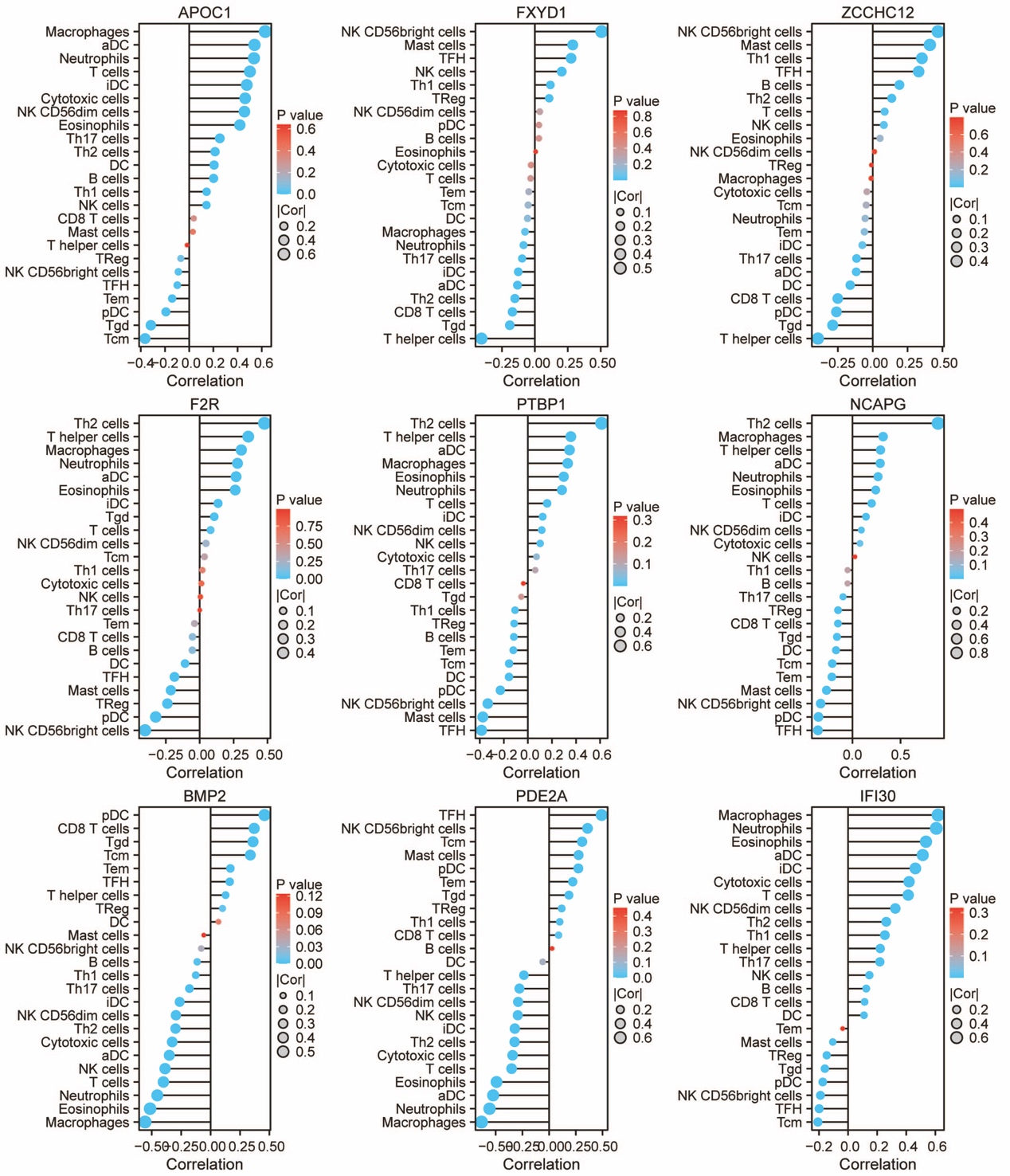


**Supplementary Figure 7.** Relationships between 9 PRGs expression profiles and the infiltration levels of 24 immune cell types.


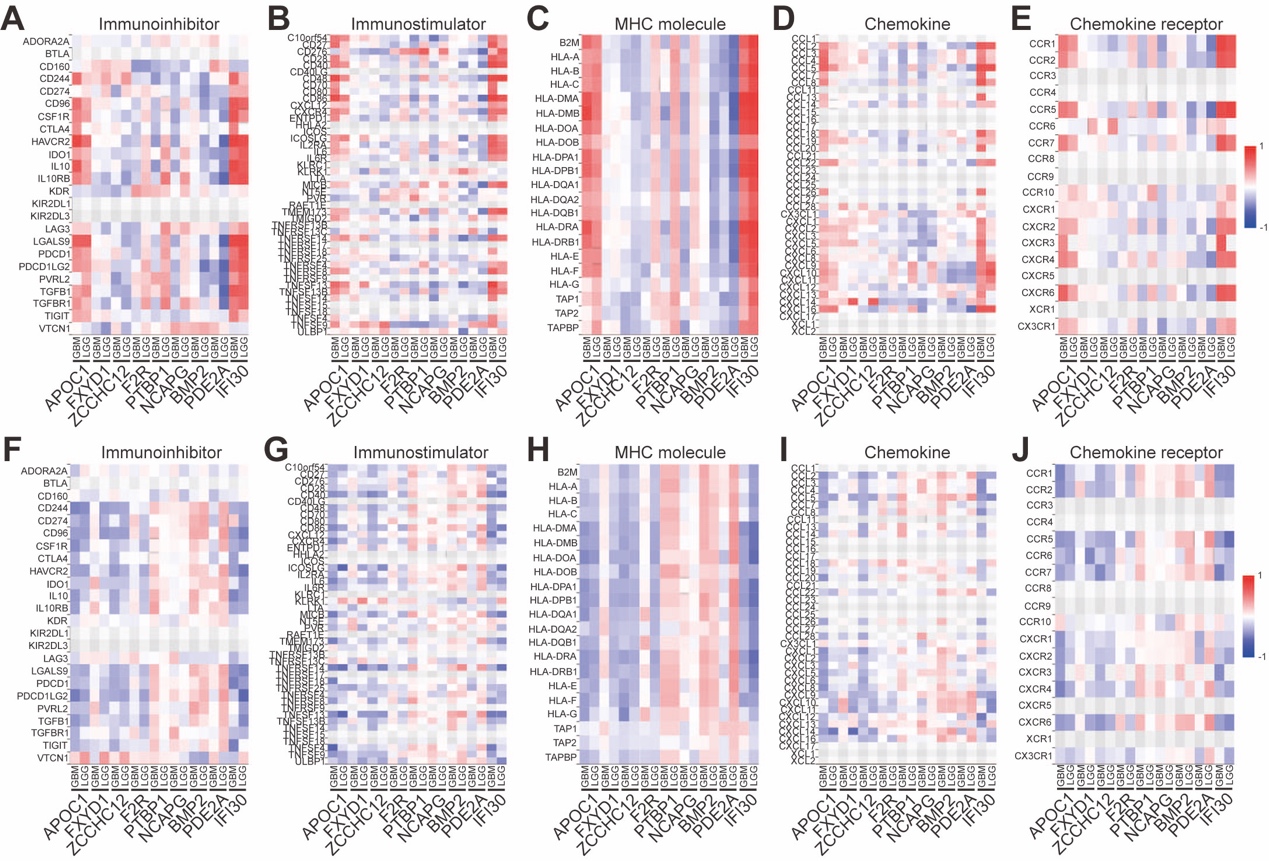


**Supplementary Figure 8.** Heatmaps showing the correlations between 9 PRGs expression levels and (**A**) immunostimulators, (**B**) immunoinhibitors, (**C**) MHC molecules, (**D**) chemokines, and (**E**) chemokine receptors in LGG and GBM. Heatmaps showing the correlations between methylation of 9 PRGs and (**F**) immunostimulators, (**G**) immunoinhibitors, (**H**) MHC molecules, (**I**) chemokines, and (**J**) chemokine receptors in LGG and GBM.


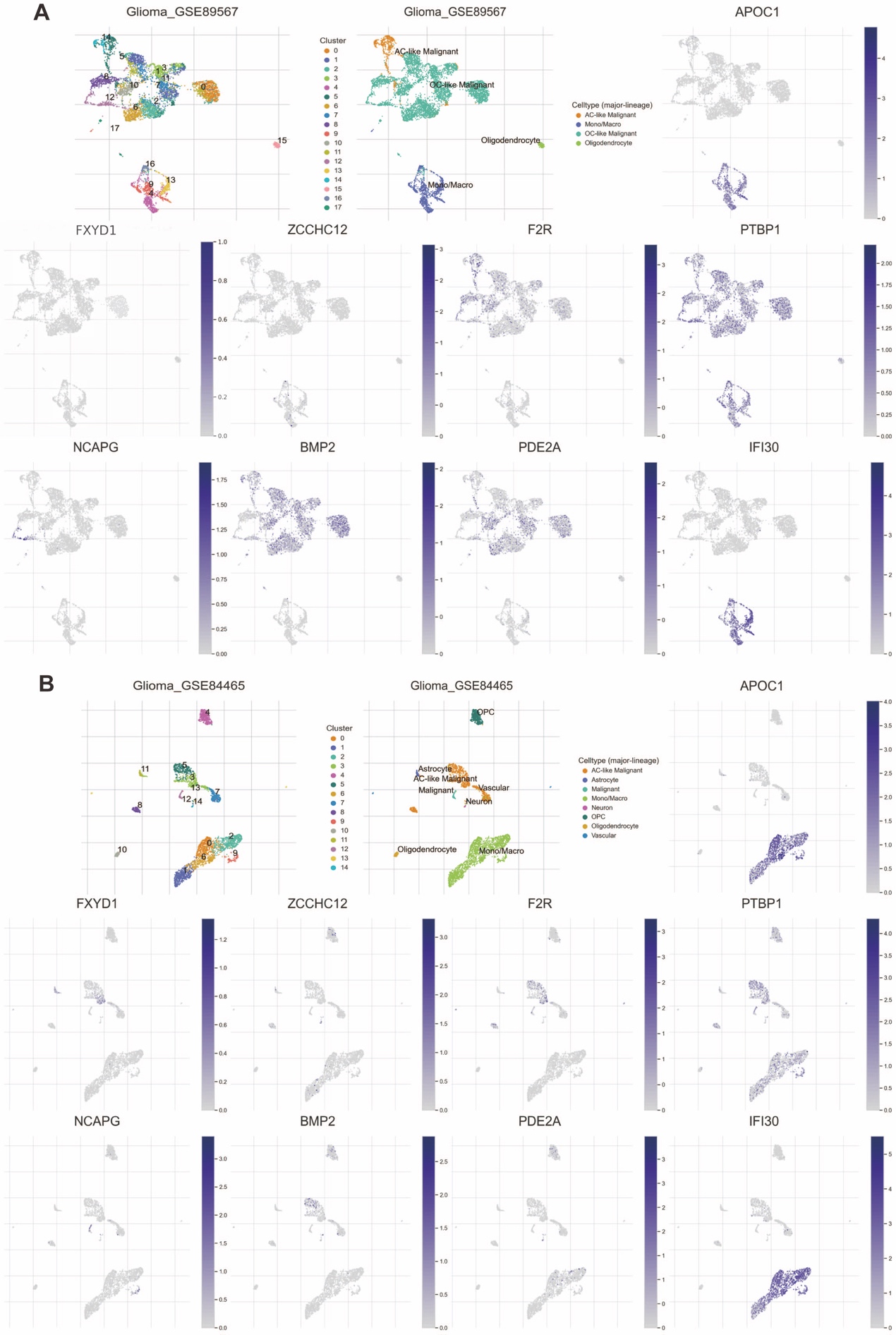


**Supplementary Figure 9.** Single-cell transcriptome analysis of PRGs expression distribution in multiple cell types in tumor microenvironment. The expression state of 9 PRGs in multiple cell types based on (**A**) GSE89567 and (**B**) GSE84465 datasets.


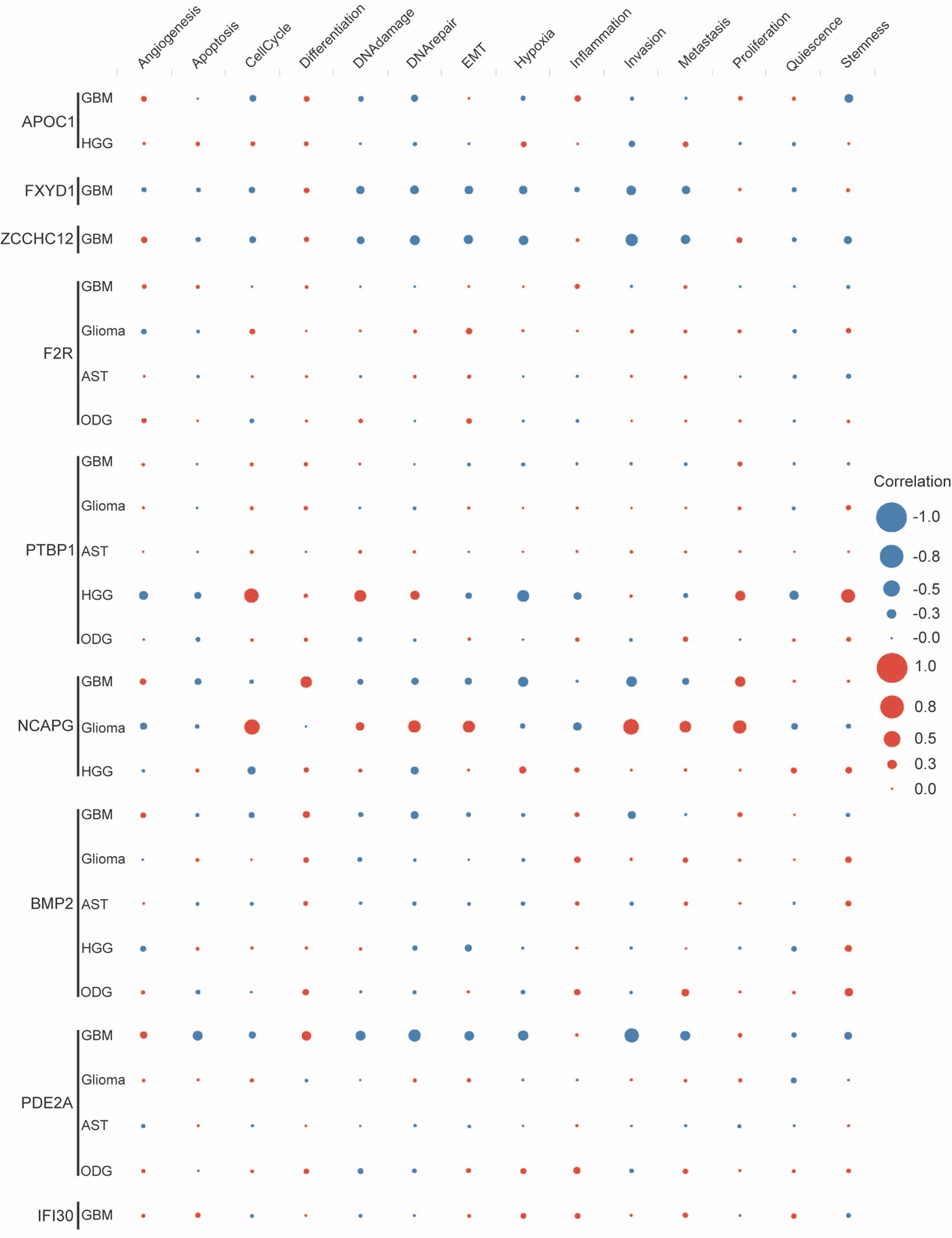


**Supplementary Figure 10.** The interactive bubble chart presents the correlation of PRGs with the functional state in glioma.


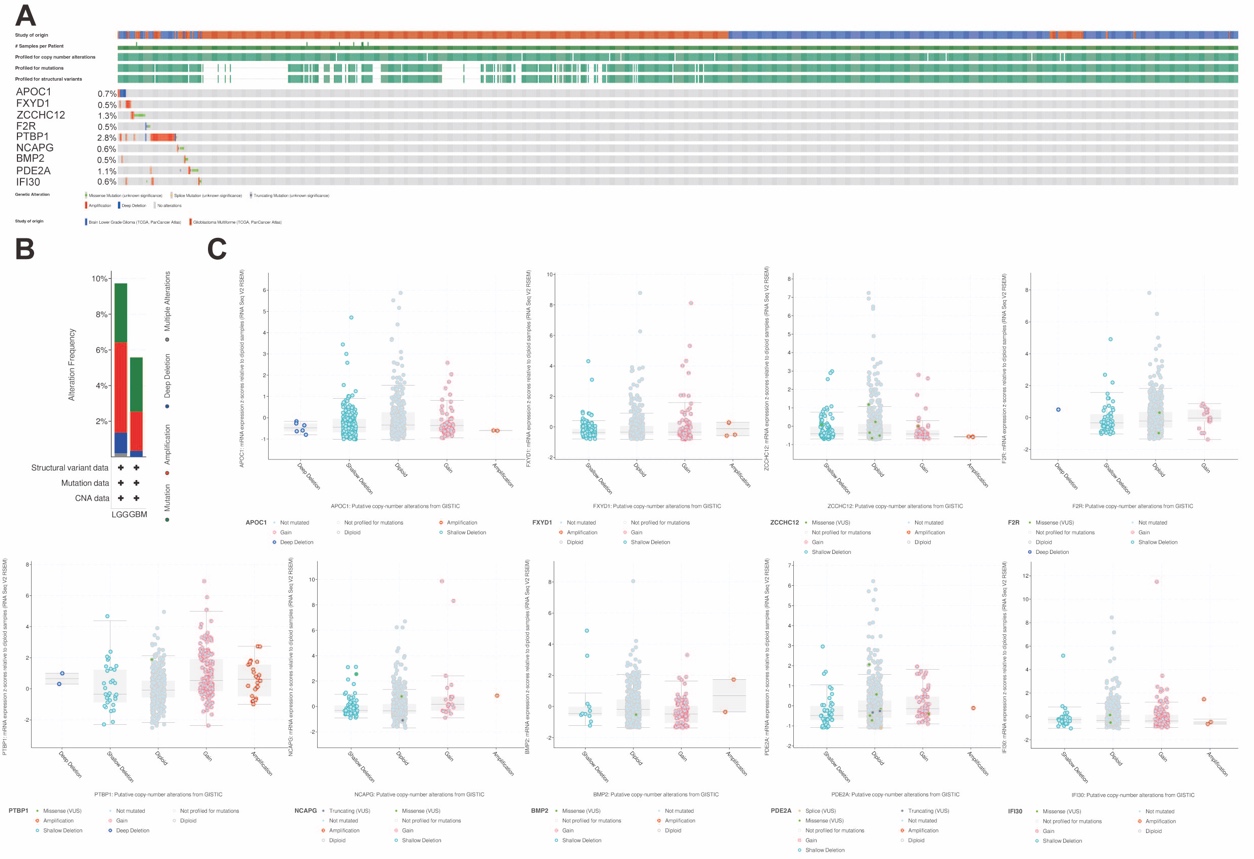


**Supplementary Figure 11.** Alterations of PRGs. (**A**) Summary of PRGs mutation rates. (**B**) Genetic alteration summary of PRGs in LGG and GBM. (**C**) PRGs mutation types.


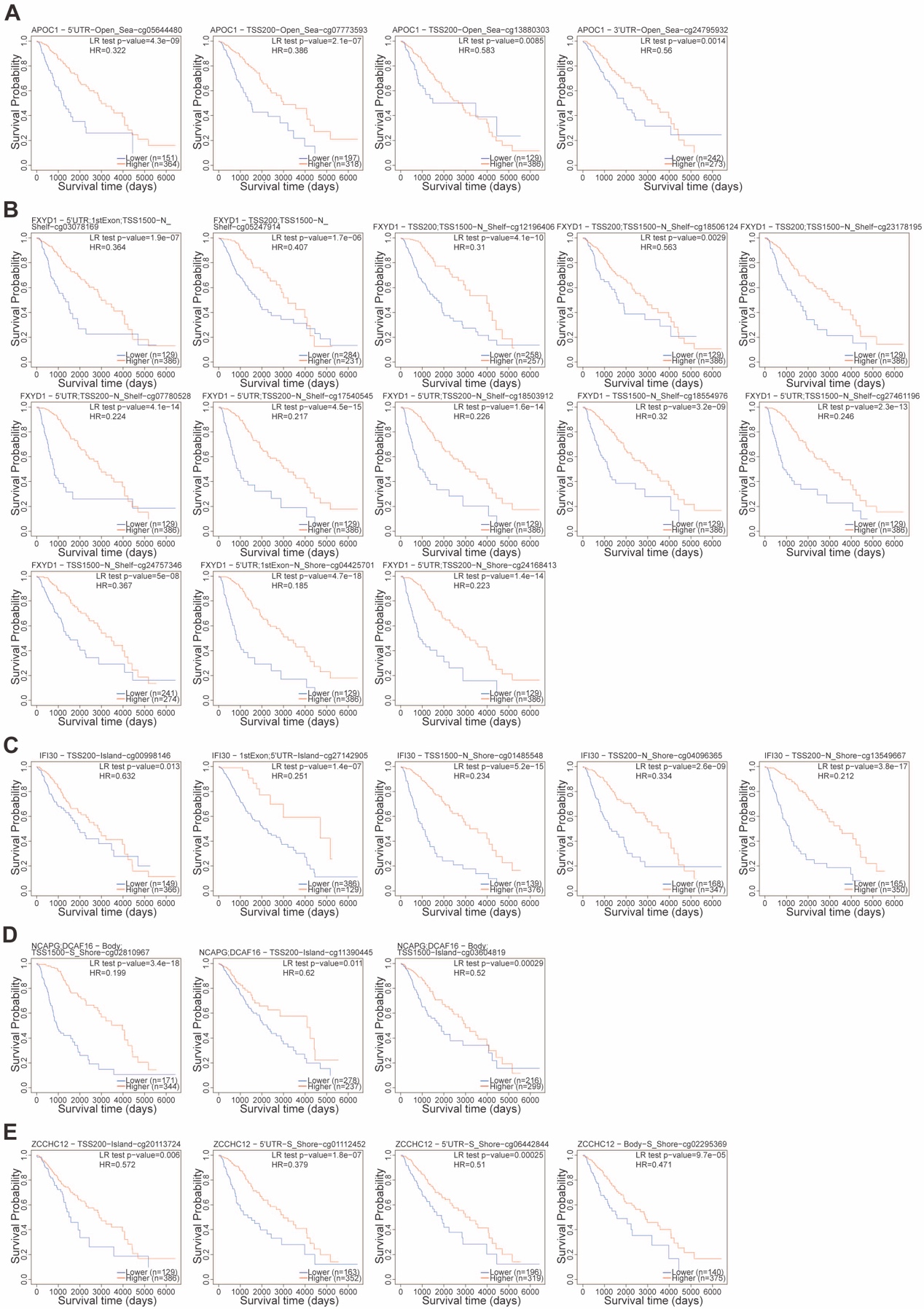


**Supplementary Figure 12.** The Kaplan–Meier survival analysis of the promoter methylation of (**A**) *APOC1*, (**B**) *FXYD1*, (**C**) *IFI30*, (**D**) *NCAPG*, and (**E**) *ZCCHC12* in LGG.


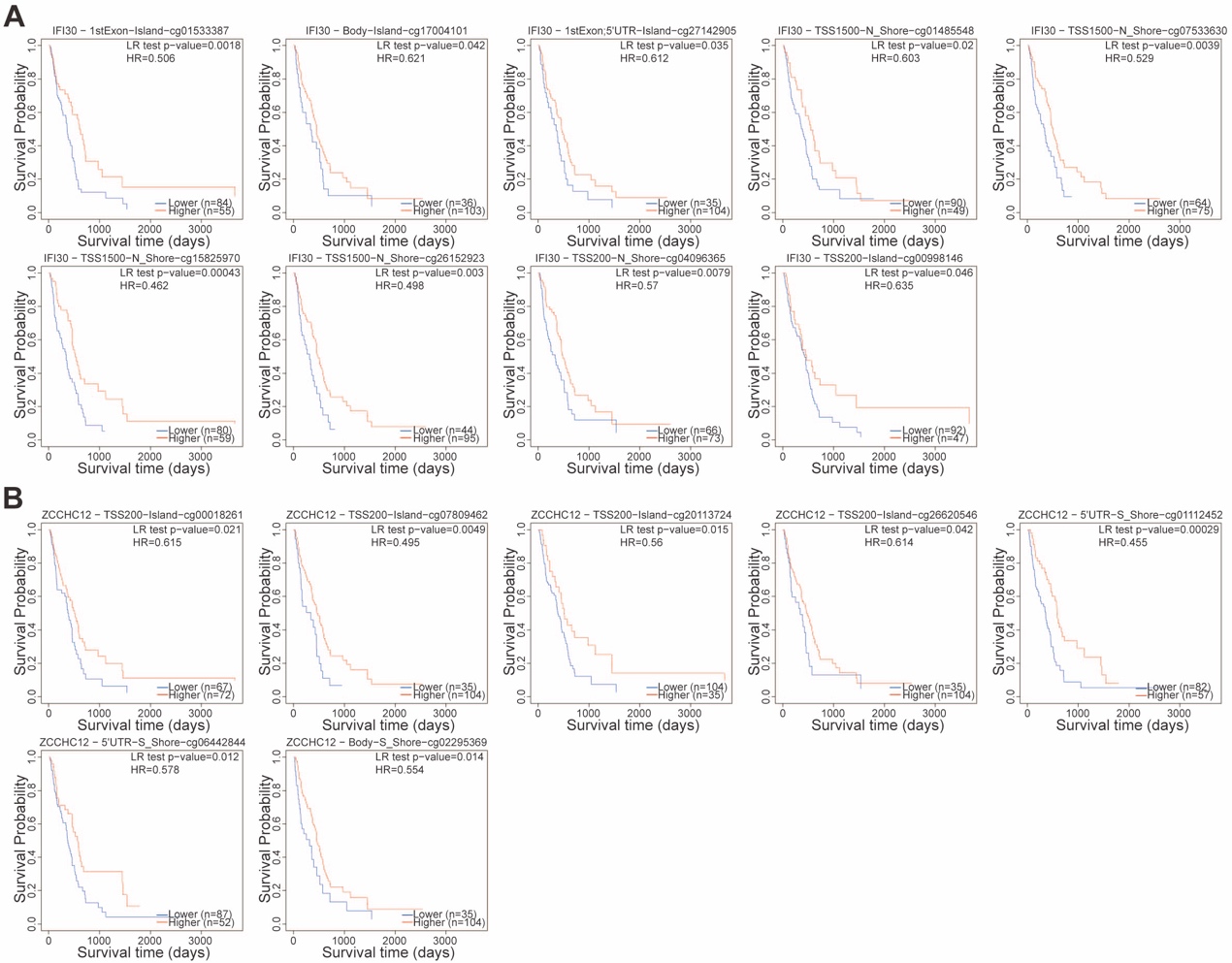


**Supplementary Figure 13.** The Kaplan–Meier survival analysis of the promoter methylation of (**A**) *IFI30* and (**B**) *ZCCHC12* in GBM.


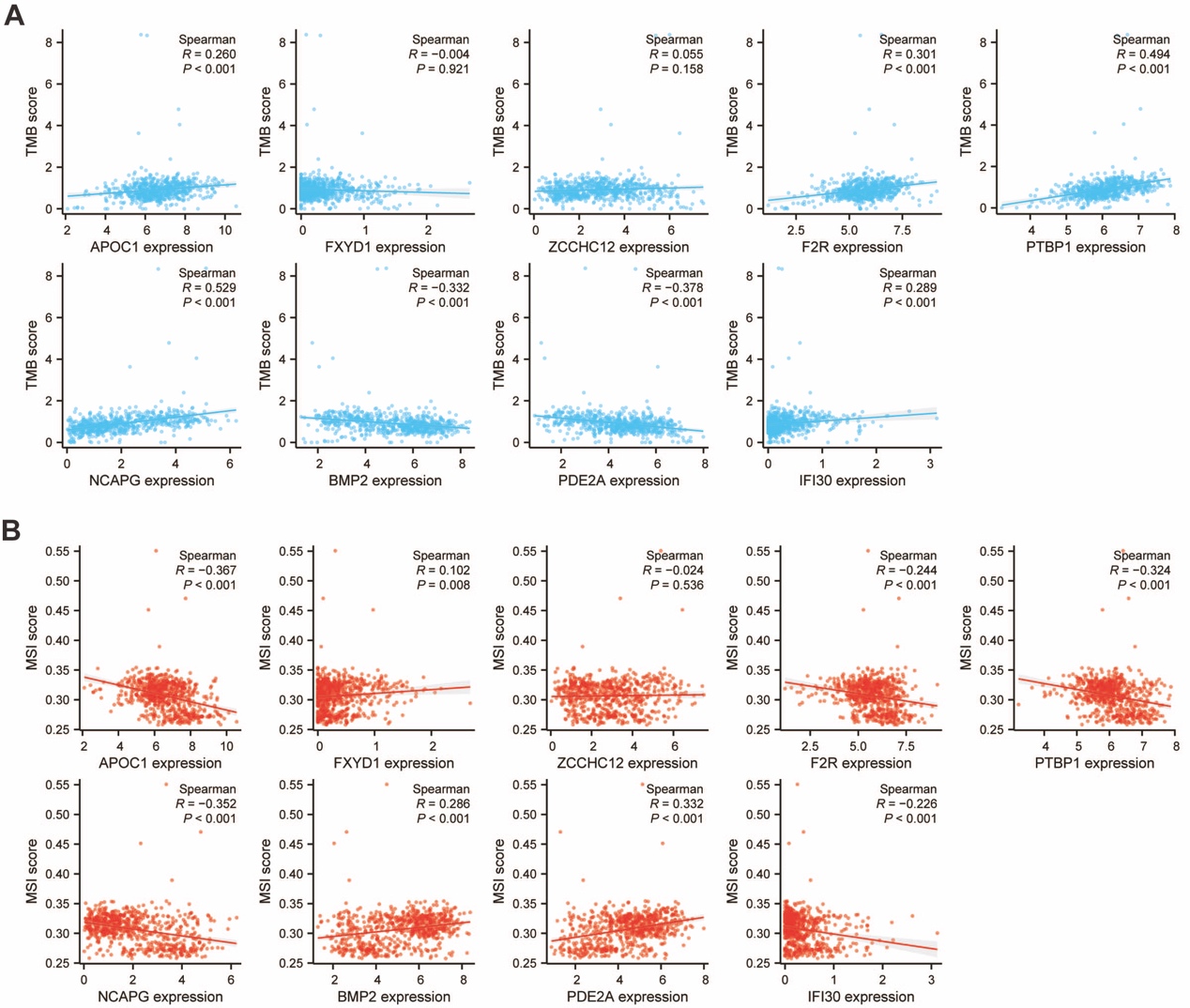


**Supplementary Figure 14.** Correlation analysis between 9 PRGs and (**A**) TMB score and (**B**) MSI score.


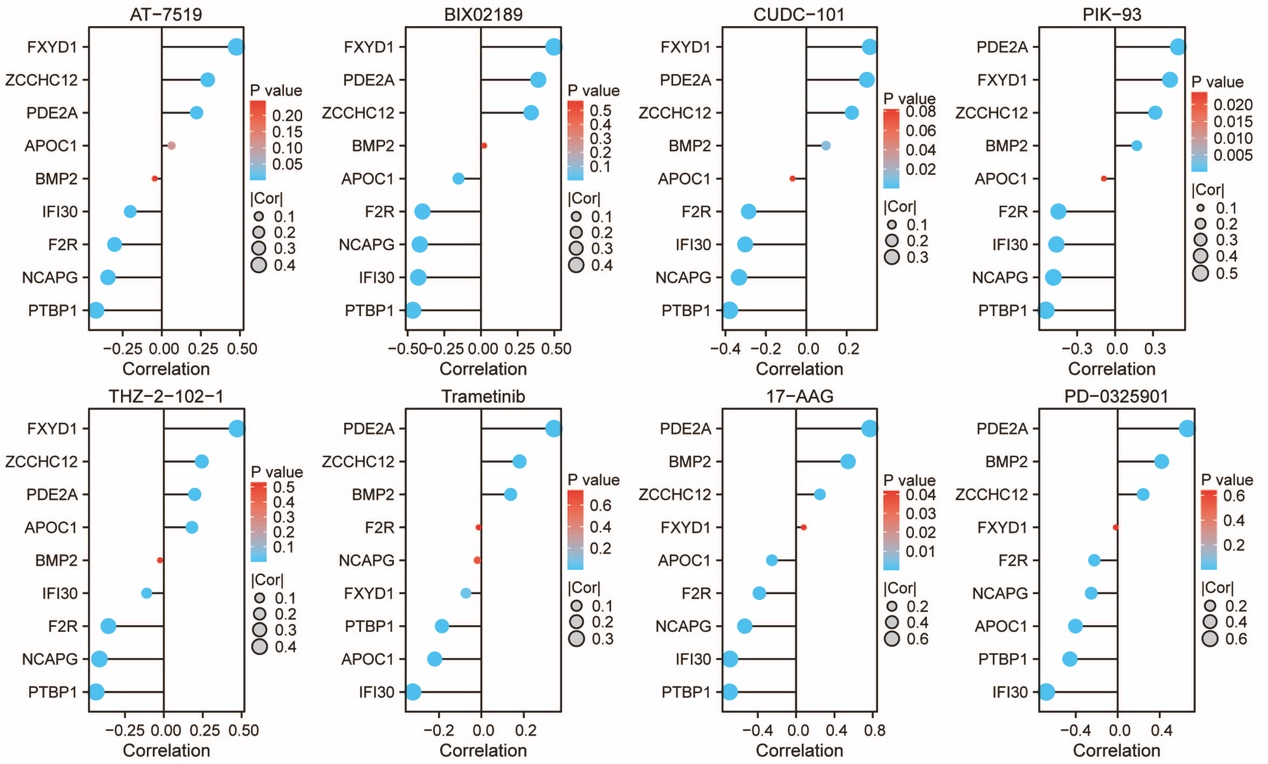


**Supplementary Figure 15.** Spearman correlation analysis of 9 PRGs and IC50 of drugs.

**Supplementary**
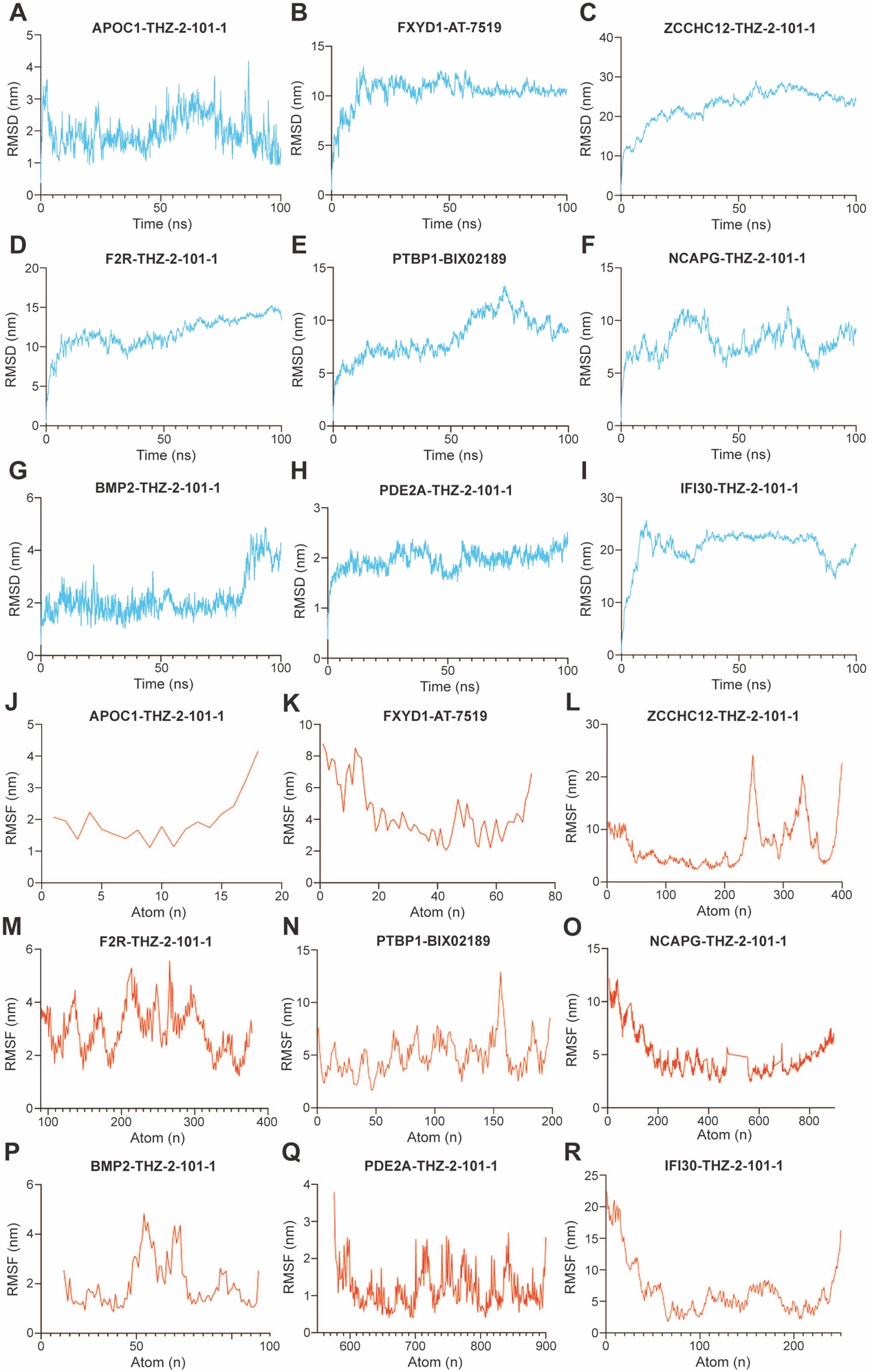
**Figure 16.** Molecular dynamics simulation results display. (**A-I**) Root mean square deviation (RMSD). (**J-R**) Root mean square fluctuation (RMSF).
