# Supplementary material for: Comprehensive analysis of palmitoylation-related proteins for prognostic risk evaluation and tumor immune microenvironment assessment in glioma
Source: Front Immunol. 2025 Dec 8;16:1599769. doi: 10.3389/fimmu.2025.1599769 (PMC12719430; doi:10.3389/fimmu.2025.1599769)
Supplement: Supplementary file 2 [file DataSheet2.docx]

| **Supplementary Table 1.** PH assumption test on the nomogram model | | | | |
| --- | --- | --- | --- | --- |
|  | **Characteristic** | **Chi-Square value** | **Degree of freedom** | ***P*-value** |
| **TCGA-GBMLGG** | WHO grade | 1.9306 | 2 | 0.3809 |
|  | Age | 1.3750 | 1 | 0.2410 |
|  | 1p/19q codeletion status | 0.0009 | 1 | 0.9767 |
|  | MGMT methylation status | 3.4402 | 1 | 0.0636 |
|  | Risk score | 2.5621 | 1 | 0.1095 |
|  | Global | 10.4980 | 6 | 0.1052 |
| If the *p*-value in the global test > 0.05, it indicates that the nomogram adheres to the PH assumption. | | | | |

| **Supplementary Table 2.** Molecular docking results | | | | |
| --- | --- | --- | --- | --- |
| **Receptor-ligand** | **mode** | **affinity (kcal/mol)** | **rmsd l.b.** | **rmsd u.b.** |
| APOC1-AT-7519 | 1 | -5.2 | 0.000 | 0.000 |
|  | 2 | -5.2 | 10.493 | 11.932 |
|  | 3 | -5.1 | 11.156 | 12.564 |
|  | 4 | -5 | 2.900 | 5.280 |
|  | 5 | -4.9 | 2.277 | 3.175 |
|  | 6 | -4.9 | 2.147 | 5.397 |
|  | 7 | -4.7 | 10.108 | 11.618 |
|  | 8 | -4.7 | 10.791 | 11.633 |
|  | 9 | -4.7 | 3.879 | 6.323 |
|  | 10 | -4.7 | 11.463 | 13.851 |
|  | 11 | -4.6 | 10.040 | 13.646 |
|  | 12 | -4.6 | 3.251 | 5.202 |
|  | 13 | -4.5 | 3.677 | 7.847 |
|  | 14 | -4.5 | 11.536 | 12.838 |
|  | 15 | -4.5 | 11.442 | 12.701 |
|  | 16 | -4.4 | 2.106 | 2.680 |
|  | 17 | -4.4 | 2.767 | 5.895 |
|  | 18 | -4.3 | 3.863 | 7.149 |
|  | 19 | -4.3 | 2.984 | 6.151 |
|  | 20 | -4.3 | 11.560 | 12.428 |
| APOC1-BIX02189 | 1 | -5.2 | 0.000 | 0.000 |
|  | 2 | -5.1 | 5.046 | 8.014 |
|  | 3 | -5.1 | 12.270 | 14.864 |
|  | 4 | -5 | 10.825 | 15.193 |
|  | 5 | -5 | 11.017 | 13.532 |
|  | 6 | -5 | 10.624 | 12.853 |
|  | 7 | -5 | 2.631 | 8.534 |
|  | 8 | -5 | 5.934 | 8.563 |
|  | 9 | -5 | 3.332 | 8.495 |
|  | 10 | -5 | 10.987 | 13.607 |
|  | 11 | -5 | 12.151 | 15.080 |
|  | 12 | -4.9 | 3.563 | 5.294 |
|  | 13 | -4.8 | 5.880 | 8.616 |
|  | 14 | -4.6 | 11.133 | 14.282 |
|  | 15 | -4.6 | 3.317 | 8.350 |
|  | 16 | -4.5 | 10.950 | 13.140 |
|  | 17 | -4.5 | 10.491 | 13.464 |
|  | 18 | -4.5 | 3.688 | 6.681 |
|  | 19 | -4.4 | 1.545 | 2.316 |
|  | 20 | -4.3 | 2.427 | 4.152 |
| APOC1-CUDC-101 | 1 | -4.8 | 0.000 | 0.000 |
|  | 2 | -4.8 | 2.407 | 5.424 |
|  | 3 | -4.7 | 11.275 | 13.378 |
|  | 4 | -4.6 | 1.627 | 2.253 |
|  | 5 | -4.5 | 11.027 | 12.690 |
|  | 6 | -4.5 | 10.589 | 13.175 |
|  | 7 | -4.5 | 3.010 | 5.737 |
|  | 8 | -4.5 | 10.483 | 12.675 |
|  | 9 | -4.4 | 10.993 | 12.382 |
|  | 10 | -4.4 | 11.193 | 12.884 |
|  | 11 | -4.3 | 2.791 | 3.951 |
|  | 12 | -4.3 | 11.168 | 13.330 |
|  | 13 | -4.2 | 11.036 | 13.511 |
|  | 14 | -4.2 | 2.075 | 2.598 |
|  | 15 | -4.2 | 10.893 | 13.218 |
|  | 16 | -4.2 | 2.999 | 6.042 |
|  | 17 | -4.1 | 3.964 | 7.971 |
|  | 18 | -4.1 | 1.377 | 2.168 |
|  | 19 | -4 | 2.887 | 6.767 |
|  | 20 | -4 | 2.340 | 6.139 |
| APOC1-PIK-93 | 1 | -5.3 | 0.000 | 0.000 |
|  | 2 | -4.9 | 10.628 | 12.658 |
|  | 3 | -4.8 | 2.473 | 3.915 |
|  | 4 | -4.8 | 11.069 | 12.511 |
|  | 5 | -4.7 | 10.652 | 12.498 |
|  | 6 | -4.5 | 2.792 | 8.189 |
|  | 7 | -4.5 | 9.916 | 11.789 |
|  | 8 | -4.5 | 2.364 | 3.039 |
|  | 9 | -4.5 | 5.030 | 6.490 |
|  | 10 | -4.4 | 11.175 | 12.067 |
|  | 11 | -4.4 | 9.731 | 11.910 |
|  | 12 | -4.4 | 3.924 | 7.502 |
|  | 13 | -4.3 | 4.529 | 7.095 |
|  | 14 | -4.3 | 10.310 | 12.297 |
|  | 15 | -4.1 | 5.657 | 11.299 |
|  | 16 | -4 | 10.141 | 12.517 |
|  | 17 | -4 | 11.524 | 12.776 |
|  | 18 | -4 | 5.616 | 8.403 |
|  | 19 | -3.9 | 11.610 | 13.799 |
|  | 20 | -3.9 | 5.740 | 7.578 |
| APOC1- THZ-2-102-1 | 1 | -5.4 | 0.000 | 0.000 |
|  | 2 | -5.2 | 11.208 | 14.373 |
|  | 3 | -5 | 2.097 | 3.807 |
|  | 4 | -4.9 | 7.691 | 9.072 |
|  | 5 | -4.9 | 11.377 | 13.985 |
|  | 6 | -4.9 | 2.493 | 5.273 |
|  | 7 | -4.8 | 10.391 | 13.775 |
|  | 8 | -4.8 | 3.993 | 9.115 |
|  | 9 | -4.8 | 11.639 | 14.276 |
|  | 10 | -4.8 | 10.765 | 12.680 |
|  | 11 | -4.7 | 11.830 | 14.583 |
|  | 12 | -4.7 | 2.151 | 5.028 |
|  | 13 | -4.7 | 2.884 | 5.882 |
|  | 14 | -4.7 | 1.958 | 2.302 |
|  | 15 | -4.7 | 11.335 | 13.894 |
|  | 16 | -4.7 | 7.760 | 9.528 |
|  | 17 | -4.7 | 10.954 | 13.845 |
|  | 18 | -4.7 | 5.503 | 10.075 |
|  | 19 | -4.6 | 11.288 | 13.578 |
|  | 20 | -4.5 | 3.324 | 5.862 |
| APOC1-Trametinib | 1 | -4.9 | 0.000 | 0.000 |
|  | 2 | -4.7 | 13.478 | 16.543 |
|  | 3 | -4.7 | 3.525 | 6.114 |
|  | 4 | -4.7 | 12.406 | 14.989 |
|  | 5 | -4.7 | 3.007 | 5.509 |
|  | 6 | -4.6 | 12.603 | 15.329 |
|  | 7 | -4.6 | 12.528 | 14.739 |
|  | 8 | -4.5 | 12.047 | 14.203 |
|  | 9 | -4.5 | 11.555 | 14.464 |
|  | 10 | -4.5 | 12.526 | 15.496 |
|  | 11 | -4.5 | 2.609 | 3.550 |
|  | 12 | -4.5 | 12.230 | 14.459 |
|  | 13 | -4.4 | 12.602 | 15.206 |
|  | 14 | -4.4 | 11.797 | 14.426 |
|  | 15 | -4.4 | 4.243 | 7.854 |
|  | 16 | -4.4 | 12.142 | 14.798 |
|  | 17 | -4.3 | 13.731 | 15.905 |
|  | 18 | -4.3 | 11.871 | 13.824 |
|  | 19 | -4.3 | 12.482 | 14.994 |
|  | 20 | -4.3 | 11.540 | 13.866 |
| APOC1-17-AAG | 1 | -4.8 | 0.000 | 0.000 |
|  | 2 | -4.7 | 2.786 | 7.263 |
|  | 3 | -4.6 | 2.098 | 8.590 |
|  | 4 | -4.5 | 2.775 | 5.657 |
|  | 5 | -4.5 | 11.622 | 13.930 |
|  | 6 | -4.4 | 13.038 | 16.095 |
|  | 7 | -4.3 | 12.558 | 16.262 |
|  | 8 | -4.3 | 12.610 | 16.060 |
|  | 9 | -4.3 | 11.980 | 14.191 |
|  | 10 | -4.3 | 12.981 | 15.831 |
|  | 11 | -4.1 | 3.410 | 7.235 |
|  | 12 | -4.1 | 11.833 | 14.214 |
|  | 13 | -4 | 3.117 | 7.293 |
|  | 14 | -4 | 13.102 | 16.805 |
|  | 15 | -4 | 12.224 | 15.386 |
|  | 16 | -4 | 12.999 | 15.745 |
|  | 17 | -4 | 12.545 | 15.395 |
|  | 18 | -3.9 | 13.379 | 17.547 |
|  | 19 | -3.8 | 5.548 | 8.525 |
|  | 20 | -3.8 | 12.386 | 15.053 |
| APOC1-PD-0325901 | 1 | -5.200 | 0.000 | 0.000 |
|  | 2 | -5.000 | 10.244 | 11.333 |
|  | 3 | -4.800 | 10.652 | 11.609 |
|  | 4 | -4.800 | 3.329 | 5.123 |
|  | 5 | -4.700 | 10.709 | 11.998 |
|  | 6 | -4.500 | 5.898 | 8.614 |
|  | 7 | -4.500 | 10.605 | 12.255 |
|  | 8 | -4.500 | 11.037 | 12.741 |
|  | 9 | -4.500 | 1.716 | 3.491 |
|  | 10 | -4.400 | 10.731 | 12.342 |
|  | 11 | -4.400 | 10.174 | 11.724 |
|  | 12 | -4.200 | 10.675 | 12.388 |
|  | 13 | -4.200 | 1.983 | 3.499 |
|  | 14 | -4.100 | 3.948 | 6.607 |
|  | 15 | -4.100 | 2.346 | 2.966 |
|  | 16 | -4.000 | 2.757 | 4.84 |
|  | 17 | -4.000 | 5.315 | 8.563 |
|  | 18 | -3.900 | 10.863 | 12.525 |
|  | 19 | -3.900 | 3.644 | 5.396 |
|  | 20 | -3.800 | 3.712 | 6.566 |
| FXYD1-AT-7519 | 1 | -6.1 | 0.000 | 0.000 |
|  | 2 | -5.6 | 3.022 | 7.666 |
|  | 3 | -5.4 | 18.026 | 20.527 |
|  | 4 | -5.4 | 2.536 | 7.150 |
|  | 5 | -5.2 | 3.287 | 6.821 |
|  | 6 | -5 | 24.668 | 26.101 |
|  | 7 | -4.9 | 18.798 | 20.974 |
|  | 8 | -4.8 | 16.814 | 19.522 |
|  | 9 | -4.8 | 9.565 | 12.193 |
|  | 10 | -4.7 | 25.772 | 27.182 |
|  | 11 | -4.7 | 18.147 | 20.638 |
|  | 12 | -4.7 | 17.847 | 21.248 |
|  | 13 | -4.6 | 26.789 | 28.520 |
|  | 14 | -4.6 | 4.074 | 7.700 |
|  | 15 | -4.5 | 23.603 | 25.027 |
|  | 16 | -4.4 | 16.929 | 19.542 |
|  | 17 | -4.4 | 18.312 | 20.710 |
|  | 18 | -4.3 | 4.034 | 8.426 |
|  | 19 | -4.3 | 25.848 | 27.314 |
|  | 20 | -4.2 | 14.772 | 17.684 |
| FXYD1-BIX02189 | 1 | -5.9 | 0.000 | 0.000 |
|  | 2 | -5.8 | 23.615 | 26.388 |
|  | 3 | -5.8 | 22.776 | 26.741 |
|  | 4 | -5.7 | 17.862 | 20.984 |
|  | 5 | -5.6 | 14.025 | 16.992 |
|  | 6 | -5.6 | 23.545 | 26.558 |
|  | 7 | -5.6 | 9.652 | 11.753 |
|  | 8 | -5.5 | 13.818 | 16.609 |
|  | 9 | -5.5 | 23.228 | 26.086 |
|  | 10 | -5.5 | 49.792 | 52.313 |
|  | 11 | -5.4 | 10.394 | 12.889 |
|  | 12 | -5.4 | 48.944 | 51.355 |
|  | 13 | -5.4 | 44.031 | 46.371 |
|  | 14 | -5.4 | 24.199 | 26.876 |
|  | 15 | -5.3 | 42.014 | 44.643 |
|  | 16 | -5.3 | 23.106 | 25.044 |
|  | 17 | -5.3 | 32.316 | 34.857 |
|  | 18 | -5.2 | 11.763 | 14.762 |
|  | 19 | -5.1 | 28.036 | 31.546 |
|  | 20 | -5.1 | 32.429 | 34.975 |
| FXYD1-CUDC-101 | 1 | -5.9 | 0.000 | 0.000 |
|  | 2 | -5.6 | 10.432 | 13.689 |
|  | 3 | -5.4 | 17.564 | 20.733 |
|  | 4 | -5.4 | 17.041 | 20.968 |
|  | 5 | -5.4 | 17.121 | 20.953 |
|  | 6 | -5.4 | 16.960 | 20.949 |
|  | 7 | -5.3 | 10.452 | 13.113 |
|  | 8 | -5.3 | 11.095 | 13.230 |
|  | 9 | -5.3 | 29.957 | 33.444 |
|  | 10 | -5.3 | 14.431 | 17.064 |
|  | 11 | -5.3 | 17.016 | 21.126 |
|  | 12 | -5.3 | 16.824 | 19.873 |
|  | 13 | -5.3 | 43.583 | 46.180 |
|  | 14 | -5.2 | 5.834 | 10.520 |
|  | 15 | -5.2 | 8.870 | 12.212 |
|  | 16 | -5.2 | 10.626 | 12.814 |
|  | 17 | -5.2 | 8.512 | 12.377 |
|  | 18 | -5.2 | 17.248 | 21.837 |
|  | 19 | -5.1 | 17.411 | 20.925 |
|  | 20 | -5.1 | 30.349 | 33.506 |
| FXYD1-PIK-93 | 1 | -5.4 | 0.000 | 0.000 |
|  | 2 | -5.2 | 4.999 | 6.589 |
|  | 3 | -5.1 | 4.029 | 7.733 |
|  | 4 | -5.1 | 14.571 | 17.176 |
|  | 5 | -4.9 | 32.855 | 35.585 |
|  | 6 | -4.9 | 17.067 | 19.928 |
|  | 7 | -4.8 | 6.262 | 9.002 |
|  | 8 | -4.8 | 14.232 | 17.301 |
|  | 9 | -4.8 | 30.405 | 33.132 |
|  | 10 | -4.8 | 31.984 | 34.155 |
|  | 11 | -4.8 | 9.851 | 12.066 |
|  | 12 | -4.7 | 10.132 | 12.582 |
|  | 13 | -4.7 | 8.176 | 11.810 |
|  | 14 | -4.7 | 6.847 | 10.185 |
|  | 15 | -4.7 | 4.167 | 4.415 |
|  | 16 | -4.7 | 5.185 | 7.605 |
|  | 17 | -4.6 | 6.263 | 9.934 |
|  | 18 | -4.6 | 6.748 | 11.593 |
|  | 19 | -4.5 | 11.054 | 13.534 |
|  | 20 | -4.4 | 19.776 | 22.871 |
| FXYD1- THZ-2-102-1 | 1 | -5.5 | 0.000 | 0.000 |
|  | 2 | -5.3 | 14.714 | 17.703 |
|  | 3 | -5.3 | 4.969 | 8.907 |
|  | 4 | -5.3 | 12.783 | 16.343 |
|  | 5 | -5.2 | 12.163 | 15.583 |
|  | 6 | -5.2 | 4.064 | 6.999 |
|  | 7 | -5.1 | 15.494 | 18.881 |
|  | 8 | -5 | 8.120 | 11.696 |
|  | 9 | -5 | 2.945 | 5.501 |
|  | 10 | -5 | 14.008 | 17.419 |
|  | 11 | -4.9 | 19.054 | 22.501 |
|  | 12 | -4.9 | 14.805 | 17.582 |
|  | 13 | -4.9 | 2.526 | 5.941 |
|  | 14 | -4.9 | 15.179 | 18.175 |
|  | 15 | -4.8 | 14.059 | 17.951 |
|  | 16 | -4.8 | 11.612 | 16.025 |
|  | 17 | -4.8 | 14.477 | 17.999 |
|  | 18 | -4.8 | 3.608 | 6.808 |
|  | 19 | -4.7 | 14.368 | 18.260 |
|  | 20 | -4.7 | 3.366 | 5.759 |
| FXYD1-Trametinib | 1 | -6.1 | 0.000 | 0.000 |
|  | 2 | -5.7 | 3.476 | 7.224 |
|  | 3 | -5.6 | 2.193 | 3.213 |
|  | 4 | -5.6 | 3.754 | 7.703 |
|  | 5 | -5.5 | 11.374 | 14.001 |
|  | 6 | -5.4 | 20.768 | 22.951 |
|  | 7 | -5.3 | 2.729 | 3.677 |
|  | 8 | -5.3 | 21.034 | 23.407 |
|  | 9 | -5.2 | 20.280 | 23.126 |
|  | 10 | -5 | 32.625 | 37.136 |
|  | 11 | -5 | 2.219 | 2.398 |
|  | 12 | -5 | 4.104 | 6.878 |
|  | 13 | -4.9 | 3.139 | 5.133 |
|  | 14 | -4.7 | 12.656 | 17.041 |
|  | 15 | -4.7 | 11.702 | 13.998 |
|  | 16 | -4.6 | 12.938 | 15.687 |
|  | 17 | -4.5 | 11.240 | 13.293 |
|  | 18 | -4.4 | 4.973 | 7.288 |
|  | 19 | -4.4 | 21.253 | 23.109 |
|  | 20 | -4.3 | 33.947 | 37.370 |
| FXYD1-17-AAG | 1 | -5.9 | 0.000 | 0.000 |
|  | 2 | -5.7 | 2.373 | 4.168 |
|  | 3 | -5.6 | 14.103 | 17.359 |
|  | 4 | -5.6 | 2.865 | 7.119 |
|  | 5 | -5.6 | 13.741 | 18.482 |
|  | 6 | -5.4 | 17.268 | 19.261 |
|  | 7 | -5.3 | 2.362 | 5.294 |
|  | 8 | -5.2 | 2.675 | 8.027 |
|  | 9 | -5.2 | 20.860 | 24.152 |
|  | 10 | -5.2 | 3.221 | 7.338 |
|  | 11 | -5.1 | 3.911 | 8.183 |
|  | 12 | -5.1 | 16.202 | 19.559 |
|  | 13 | -5 | 16.674 | 19.933 |
|  | 14 | -5 | 19.216 | 23.373 |
|  | 15 | -4.9 | 2.321 | 7.726 |
|  | 16 | -4.9 | 11.053 | 15.107 |
|  | 17 | -4.9 | 15.131 | 18.246 |
|  | 18 | -4.8 | 36.824 | 39.465 |
|  | 19 | -4.8 | 38.626 | 42.194 |
|  | 20 | -4.6 | 20.179 | 23.065 |
| FXYD1-PD-0325901 | 1 | -5 | 0.000 | 0.000 |
|  | 2 | -4.5 | 3.687 | 5.445 |
|  | 3 | -4.5 | 15.968 | 18.637 |
|  | 4 | -4.4 | 12.592 | 13.833 |
|  | 5 | -4.4 | 12.243 | 13.822 |
|  | 6 | -4.3 | 31.282 | 33.714 |
|  | 7 | -4.3 | 1.759 | 2.089 |
|  | 8 | -4.3 | 12.920 | 15.141 |
|  | 9 | -4.2 | 3.926 | 6.772 |
|  | 10 | -4.2 | 13.182 | 15.009 |
|  | 11 | -4.2 | 13.302 | 15.650 |
|  | 12 | -4.1 | 13.865 | 15.257 |
|  | 13 | -4.1 | 13.189 | 15.054 |
|  | 14 | -4.1 | 6.750 | 9.553 |
|  | 15 | -4 | 12.948 | 14.632 |
|  | 16 | -4 | 14.304 | 16.086 |
|  | 17 | -3.9 | 4.128 | 6.413 |
|  | 18 | -3.9 | 3.851 | 5.881 |
|  | 19 | -3.9 | 17.051 | 19.109 |
|  | 20 | -3.9 | 12.665 | 14.396 |
| ZCCHC12-AT-7519 | 1 | -6.5 | 0.000 | 0.000 |
|  | 2 | -6.1 | 18.749 | 20.438 |
|  | 3 | -6 | 32.679 | 35.046 |
|  | 4 | -5.9 | 32.907 | 35.798 |
|  | 5 | -5.7 | 2.569 | 4.718 |
|  | 6 | -5.1 | 32.222 | 34.848 |
|  | 7 | -5.1 | 23.520 | 25.759 |
|  | 8 | -5.1 | 36.371 | 39.766 |
|  | 9 | -5 | 19.578 | 22.386 |
|  | 10 | -4.9 | 27.350 | 28.218 |
|  | 11 | -4.8 | 30.799 | 32.998 |
|  | 12 | -4.7 | 26.178 | 29.582 |
|  | 13 | -4.6 | 2.633 | 6.646 |
|  | 14 | -4.6 | 30.856 | 31.757 |
|  | 15 | -4.6 | 27.903 | 29.474 |
|  | 16 | -4.5 | 17.437 | 19.297 |
|  | 17 | -4.5 | 30.261 | 33.776 |
|  | 18 | -4.4 | 15.346 | 18.371 |
|  | 19 | -4.3 | 27.530 | 28.678 |
|  | 20 | -4.3 | 19.516 | 22.926 |
| ZCCHC12-BIX02189 | 1 | -6.9 | 0.000 | 0.000 |
|  | 2 | -6.1 | 25.292 | 28.742 |
|  | 3 | -6 | 2.241 | 2.630 |
|  | 4 | -5.9 | 25.923 | 29.454 |
|  | 5 | -5.9 | 30.478 | 33.468 |
|  | 6 | -5.9 | 28.942 | 31.823 |
|  | 7 | -5.7 | 30.272 | 33.354 |
|  | 8 | -5.7 | 44.861 | 47.787 |
|  | 9 | -5.6 | 46.783 | 49.870 |
|  | 10 | -5.5 | 45.399 | 48.456 |
|  | 11 | -5.4 | 27.430 | 31.690 |
|  | 12 | -5.4 | 50.401 | 53.993 |
|  | 13 | -5.3 | 23.095 | 24.689 |
|  | 14 | -5.3 | 14.612 | 18.903 |
|  | 15 | -5.2 | 49.764 | 54.240 |
|  | 16 | -5.2 | 29.133 | 32.091 |
|  | 17 | -5.1 | 14.619 | 18.798 |
|  | 18 | -5.1 | 27.600 | 30.351 |
|  | 19 | -5 | 9.108 | 11.335 |
|  | 20 | -5 | 26.068 | 30.046 |
| ZCCHC12-CUDC-101 | 1 | -6.2 | 0.000 | 0.000 |
|  | 2 | -5.6 | 1.558 | 2.071 |
|  | 3 | -5.2 | 2.278 | 4.709 |
|  | 4 | -5 | 10.293 | 13.011 |
|  | 5 | -4.9 | 2.665 | 6.139 |
|  | 6 | -4.7 | 2.323 | 5.516 |
|  | 7 | -4.6 | 24.142 | 25.986 |
|  | 8 | -4.6 | 2.224 | 5.661 |
|  | 9 | -4.4 | 29.335 | 32.049 |
|  | 10 | -4.3 | 25.061 | 27.644 |
|  | 11 | -4.2 | 25.478 | 29.162 |
|  | 12 | -4.2 | 23.718 | 25.774 |
|  | 13 | -4.1 | 25.839 | 27.545 |
|  | 14 | -4.1 | 23.818 | 25.900 |
|  | 15 | -4.1 | 25.341 | 29.300 |
|  | 16 | -4.1 | 3.401 | 6.398 |
|  | 17 | -4 | 13.923 | 17.255 |
|  | 18 | -4 | 28.780 | 31.586 |
|  | 19 | -4 | 23.136 | 26.944 |
|  | 20 | -4 | 23.070 | 26.073 |
| ZCCHC12-PIK-93 | 1 | -6.2 | 0.000 | 0.000 |
|  | 2 | -6.1 | 1.885 | 1.987 |
|  | 3 | -5.8 | 1.913 | 2.279 |
|  | 4 | -5.4 | 34.237 | 36.443 |
|  | 5 | -5.4 | 26.334 | 29.020 |
|  | 6 | -5.3 | 24.166 | 25.565 |
|  | 7 | -5.2 | 28.793 | 31.423 |
|  | 8 | -5.1 | 24.414 | 25.447 |
|  | 9 | -5.1 | 26.190 | 27.378 |
|  | 10 | -5 | 33.253 | 35.485 |
|  | 11 | -5 | 46.165 | 47.606 |
|  | 12 | -4.9 | 6.247 | 7.104 |
|  | 13 | -4.8 | 22.935 | 23.771 |
|  | 14 | -4.6 | 28.790 | 31.983 |
|  | 15 | -4.6 | 20.329 | 24.589 |
|  | 16 | -4.5 | 24.491 | 26.942 |
|  | 17 | -4.4 | 2.620 | 3.600 |
|  | 18 | -4.4 | 5.670 | 7.272 |
|  | 19 | -4.4 | 29.432 | 31.416 |
|  | 20 | -4.3 | 6.317 | 7.941 |
| ZCCHC12- THZ-2-102-1 | 1 | -8 | 0.000 | 0.000 |
|  | 2 | -7.3 | 35.153 | 38.597 |
|  | 3 | -7.2 | 3.460 | 4.788 |
|  | 4 | -7.1 | 26.051 | 29.647 |
|  | 5 | -7.1 | 6.711 | 10.421 |
|  | 6 | -7.1 | 26.235 | 28.751 |
|  | 7 | -7.1 | 7.060 | 11.510 |
|  | 8 | -7 | 22.461 | 25.580 |
|  | 9 | -7 | 25.332 | 26.875 |
|  | 10 | -6.9 | 14.214 | 18.184 |
|  | 11 | -6.9 | 14.294 | 21.552 |
|  | 12 | -6.9 | 5.976 | 11.473 |
|  | 13 | -6.8 | 25.675 | 28.296 |
|  | 14 | -6.8 | 13.683 | 17.519 |
|  | 15 | -6.7 | 26.087 | 29.090 |
|  | 16 | -6.7 | 24.216 | 27.710 |
|  | 17 | -6.6 | 21.550 | 24.040 |
|  | 18 | -6.6 | 19.725 | 24.091 |
|  | 19 | -6.6 | 21.265 | 24.162 |
|  | 20 | -6.6 | 25.162 | 30.706 |
| ZCCHC12-Trametinib | 1 | -6.8 | 0.000 | 0.000 |
|  | 2 | -6.7 | 3.661 | 8.875 |
|  | 3 | -6.1 | 4.152 | 6.450 |
|  | 4 | -6.1 | 28.909 | 32.449 |
|  | 5 | -6.1 | 27.064 | 30.346 |
|  | 6 | -6.1 | 39.659 | 43.305 |
|  | 7 | -6 | 3.346 | 6.572 |
|  | 8 | -5.9 | 39.581 | 42.313 |
|  | 9 | -5.8 | 3.784 | 8.256 |
|  | 10 | -5.7 | 2.854 | 4.768 |
|  | 11 | -5.7 | 3.002 | 4.924 |
|  | 12 | -5.6 | 2.809 | 4.326 |
|  | 13 | -5.5 | 26.837 | 30.858 |
|  | 14 | -5.4 | 5.383 | 9.302 |
|  | 15 | -5.4 | 39.631 | 41.635 |
|  | 16 | -5.4 | 39.258 | 42.024 |
|  | 17 | -5.4 | 39.193 | 41.706 |
|  | 18 | -5.4 | 39.797 | 42.391 |
|  | 19 | -5.1 | 31.184 | 35.213 |
|  | 20 | -5.1 | 2.719 | 3.637 |
| ZCCHC12-17-AAG | 1 | -6.3 | 0.000 | 0.000 |
|  | 2 | -5.8 | 26.730 | 30.150 |
|  | 3 | -5.7 | 29.137 | 32.316 |
|  | 4 | -5.6 | 24.712 | 28.775 |
|  | 5 | -5.5 | 27.765 | 31.454 |
|  | 6 | -5.5 | 26.613 | 29.592 |
|  | 7 | -5.4 | 3.583 | 6.911 |
|  | 8 | -5.4 | 26.563 | 30.125 |
|  | 9 | -5.3 | 22.576 | 25.773 |
|  | 10 | -5 | 12.277 | 15.980 |
|  | 11 | -4.9 | 28.001 | 30.665 |
|  | 12 | -4.8 | 35.276 | 38.179 |
|  | 13 | -4.8 | 44.932 | 48.413 |
|  | 14 | -4.8 | 23.629 | 28.195 |
|  | 15 | -4.8 | 35.672 | 39.391 |
|  | 16 | -4.6 | 34.657 | 37.658 |
|  | 17 | -4.5 | 44.327 | 49.016 |
|  | 18 | -4.5 | 24.441 | 28.501 |
|  | 19 | -4.4 | 68.837 | 72.373 |
|  | 20 | -4.4 | 23.148 | 27.866 |
| ZCCHC12-PD-0325901 | 1 | -5.6 | 0.000 | 0.000 |
|  | 2 | -5.3 | 1.963 | 2.257 |
|  | 3 | -4.6 | 45.813 | 47.384 |
|  | 4 | -4.5 | 3.506 | 5.341 |
|  | 5 | -4.4 | 27.493 | 29.815 |
|  | 6 | -4.3 | 10.661 | 12.769 |
|  | 7 | -4.2 | 37.242 | 38.767 |
|  | 8 | -4.1 | 24.881 | 26.684 |
|  | 9 | -4.1 | 13.860 | 15.970 |
|  | 10 | -4 | 29.409 | 31.338 |
|  | 11 | -4 | 24.177 | 25.892 |
|  | 12 | -4 | 26.564 | 28.716 |
|  | 13 | -3.9 | 37.528 | 38.647 |
|  | 14 | -3.9 | 13.765 | 15.437 |
|  | 15 | -3.9 | 30.496 | 32.738 |
|  | 16 | -3.8 | 15.077 | 17.893 |
|  | 17 | -3.8 | 19.913 | 22.126 |
|  | 18 | -3.7 | 19.324 | 21.339 |
|  | 19 | -3.6 | 26.162 | 28.772 |
|  | 20 | -3.5 | 22.734 | 25.036 |
| F2R-AT-7519 | 1 | -7 | 0.000 | 0.000 |
|  | 2 | -6.9 | 45.343 | 48.573 |
|  | 3 | -6.4 | 10.205 | 14.467 |
|  | 4 | -6.4 | 22.193 | 25.777 |
|  | 5 | -6.3 | 29.851 | 33.117 |
|  | 6 | -6.3 | 2.260 | 7.004 |
|  | 7 | -6.2 | 22.816 | 25.814 |
|  | 8 | -6.2 | 16.832 | 20.669 |
|  | 9 | -6.2 | 50.450 | 54.584 |
|  | 10 | -6.1 | 16.806 | 20.580 |
|  | 11 | -6.1 | 25.935 | 28.592 |
|  | 12 | -6.1 | 22.472 | 25.370 |
|  | 13 | -6 | 16.829 | 19.927 |
|  | 14 | -6 | 25.887 | 28.974 |
|  | 15 | -5.9 | 45.096 | 48.384 |
|  | 16 | -5.8 | 27.743 | 30.280 |
|  | 17 | -5.8 | 42.071 | 46.750 |
|  | 18 | -5.7 | 27.655 | 30.612 |
|  | 19 | -5.7 | 29.856 | 32.866 |
|  | 20 | -5.6 | 2.919 | 7.316 |
| F2R-BIX02189 | 1 | -10.1 | 0.000 | 0.000 |
|  | 2 | -10 | 1.674 | 2.132 |
|  | 3 | -9 | 1.833 | 2.615 |
|  | 4 | -8 | 35.916 | 39.455 |
|  | 5 | -7.9 | 15.615 | 18.264 |
|  | 6 | -7.6 | 15.900 | 19.133 |
|  | 7 | -7.5 | 33.632 | 37.477 |
|  | 8 | -7.5 | 2.484 | 3.654 |
|  | 9 | -7.4 | 15.046 | 17.740 |
|  | 10 | -7.4 | 72.136 | 76.107 |
|  | 11 | -7.3 | 15.979 | 19.265 |
|  | 12 | -7.2 | 50.815 | 54.183 |
|  | 13 | -7.2 | 72.593 | 76.526 |
|  | 14 | -7.1 | 71.676 | 74.707 |
|  | 15 | -7.1 | 30.372 | 34.330 |
|  | 16 | -7.1 | 71.786 | 75.094 |
|  | 17 | -7 | 72.159 | 74.787 |
|  | 18 | -6.9 | 30.105 | 33.928 |
|  | 19 | -6.8 | 35.920 | 38.831 |
|  | 20 | -6.7 | 14.420 | 17.751 |
| F2R-CUDC-101 | 1 | -6.7 | 0.000 | 0.000 |
|  | 2 | -6.7 | 2.182 | 2.364 |
|  | 3 | -6.6 | 44.419 | 47.632 |
|  | 4 | -6.6 | 44.158 | 47.439 |
|  | 5 | -6.5 | 2.898 | 3.943 |
|  | 6 | -6.4 | 3.206 | 7.009 |
|  | 7 | -6.4 | 43.923 | 47.073 |
|  | 8 | -6.4 | 35.162 | 38.968 |
|  | 9 | -6.3 | 45.404 | 47.494 |
|  | 10 | -6.3 | 44.977 | 47.775 |
|  | 11 | -6.3 | 22.615 | 25.560 |
|  | 12 | -6.3 | 22.458 | 25.225 |
|  | 13 | -6.3 | 43.858 | 47.152 |
|  | 14 | -6.3 | 44.363 | 47.362 |
|  | 15 | -6.2 | 44.297 | 47.537 |
|  | 16 | -6.2 | 23.298 | 25.705 |
|  | 17 | -6.2 | 27.307 | 29.988 |
|  | 18 | -6 | 43.985 | 47.166 |
|  | 19 | -5.8 | 22.053 | 25.343 |
|  | 20 | -5.8 | 37.612 | 40.412 |
| F2R-PIK-93 | 1 | -6.4 | 0.000 | 0.000 |
|  | 2 | -6.3 | 80.069 | 82.700 |
|  | 3 | -6.3 | 9.130 | 14.507 |
|  | 4 | -6.2 | 16.373 | 20.007 |
|  | 5 | -6.1 | 20.607 | 24.747 |
|  | 6 | -6.1 | 1.990 | 2.871 |
|  | 7 | -6 | 45.658 | 47.577 |
|  | 8 | -5.9 | 10.738 | 15.388 |
|  | 9 | -5.9 | 16.375 | 18.738 |
|  | 10 | -5.8 | 23.758 | 27.480 |
|  | 11 | -5.7 | 80.690 | 82.548 |
|  | 12 | -5.7 | 13.981 | 18.088 |
|  | 13 | -5.6 | 43.663 | 45.670 |
|  | 14 | -5.6 | 82.277 | 83.746 |
|  | 15 | -5.6 | 44.011 | 45.912 |
|  | 16 | -5.5 | 82.996 | 85.980 |
|  | 17 | -5.5 | 82.076 | 83.159 |
|  | 18 | -5.5 | 80.901 | 83.916 |
|  | 19 | -5.4 | 46.003 | 50.342 |
|  | 20 | -5.4 | 1.650 | 2.049 |
| F2R-THZ-2-102-1 | 1 | -11 | 0.000 | 0.000 |
|  | 2 | -10.3 | 1.778 | 2.302 |
|  | 3 | -10 | 9.591 | 12.861 |
|  | 4 | -9.8 | 8.794 | 11.318 |
|  | 5 | -9.6 | 9.494 | 12.787 |
|  | 6 | -9.5 | 7.251 | 12.629 |
|  | 7 | -9.4 | 66.413 | 69.752 |
|  | 8 | -9.4 | 8.174 | 12.866 |
|  | 9 | -9.3 | 8.662 | 11.134 |
|  | 10 | -9.2 | 9.854 | 13.247 |
|  | 11 | -9.2 | 6.574 | 12.401 |
|  | 12 | -9.1 | 66.402 | 69.735 |
|  | 13 | -9 | 9.998 | 13.560 |
|  | 14 | -9 | 9.658 | 13.108 |
|  | 15 | -8.9 | 67.205 | 70.526 |
|  | 16 | -8.9 | 66.404 | 70.040 |
|  | 17 | -8.9 | 66.549 | 69.288 |
|  | 18 | -8.8 | 66.894 | 70.327 |
|  | 19 | -8.6 | 66.754 | 70.284 |
|  | 20 | -8.6 | 22.321 | 27.073 |
| F2R-Trametinib | 1 | -8.1 | 0.000 | 0.000 |
|  | 2 | -7.9 | 3.298 | 5.348 |
|  | 3 | -7.8 | 3.264 | 6.109 |
|  | 4 | -7.7 | 3.743 | 7.658 |
|  | 5 | -7.7 | 12.293 | 14.780 |
|  | 6 | -7.5 | 3.385 | 5.923 |
|  | 7 | -7.4 | 2.610 | 3.271 |
|  | 8 | -7.4 | 13.255 | 16.285 |
|  | 9 | -7.3 | 3.893 | 6.844 |
|  | 10 | -7.2 | 15.494 | 18.795 |
|  | 11 | -7 | 2.914 | 5.617 |
|  | 12 | -6.9 | 15.807 | 18.555 |
|  | 13 | -6.7 | 4.370 | 6.647 |
|  | 14 | -6.5 | 66.983 | 68.765 |
|  | 15 | -6.5 | 15.651 | 18.327 |
|  | 16 | -6.5 | 63.110 | 64.888 |
|  | 17 | -6.4 | 3.552 | 4.966 |
|  | 18 | -6.4 | 21.524 | 24.392 |
|  | 19 | -6.4 | 48.904 | 51.282 |
|  | 20 | -6.2 | 14.286 | 18.033 |
| F2R-17-AAG | 1 | -6.8 | 0.000 | 0.000 |
|  | 2 | -6.4 | 20.554 | 24.142 |
|  | 3 | -6.4 | 4.356 | 6.731 |
|  | 4 | -6.4 | 2.094 | 3.510 |
|  | 5 | -6.3 | 45.059 | 49.222 |
|  | 6 | -6.3 | 14.651 | 18.725 |
|  | 7 | -6.2 | 3.413 | 5.445 |
|  | 8 | -6.2 | 8.866 | 13.957 |
|  | 9 | -6.2 | 74.756 | 79.342 |
|  | 10 | -6.2 | 2.142 | 7.015 |
|  | 11 | -6.1 | 4.114 | 7.325 |
|  | 12 | -6.1 | 45.833 | 50.276 |
|  | 13 | -6.1 | 12.786 | 17.674 |
|  | 14 | -6.1 | 15.811 | 18.739 |
|  | 15 | -6 | 25.526 | 29.226 |
|  | 16 | -5.9 | 60.821 | 65.705 |
|  | 17 | -5.8 | 14.585 | 19.139 |
|  | 18 | -5.8 | 65.678 | 70.471 |
|  | 19 | -5.8 | 59.174 | 63.313 |
|  | 20 | -5.7 | 25.337 | 28.280 |
| F2R-PD-0325901 | 1 | -8.8 | 0.000 | 0.000 |
|  | 2 | -7.1 | 2.309 | 2.707 |
|  | 3 | -6.9 | 69.659 | 71.859 |
|  | 4 | -6.5 | 69.845 | 72.044 |
|  | 5 | -6.2 | 36.394 | 38.467 |
|  | 6 | -6.1 | 35.742 | 37.935 |
|  | 7 | -5.9 | 63.133 | 65.228 |
|  | 8 | -5.9 | 47.747 | 49.247 |
|  | 9 | -5.8 | 36.629 | 38.531 |
|  | 10 | -5.7 | 70.516 | 72.772 |
|  | 11 | -5.7 | 70.903 | 73.068 |
|  | 12 | -5.6 | 34.233 | 36.279 |
|  | 13 | -5.6 | 16.010 | 18.366 |
|  | 14 | -5.4 | 70.466 | 72.689 |
|  | 15 | -5.3 | 77.673 | 79.944 |
|  | 16 | -5.3 | 77.182 | 78.793 |
|  | 17 | -5.2 | 36.116 | 37.833 |
|  | 18 | -5.2 | 70.750 | 72.990 |
|  | 19 | -5.1 | 34.103 | 35.994 |
|  | 20 | -5.1 | 71.394 | 73.606 |
| PTBP1-AT-7519 | 1 | -6.9 | 0.000 | 0.000 |
|  | 2 | -6.7 | 2.840 | 6.219 |
|  | 3 | -6.6 | 3.090 | 7.088 |
|  | 4 | -6.5 | 1.962 | 2.497 |
|  | 5 | -6.3 | 2.898 | 5.788 |
|  | 6 | -6.2 | 14.074 | 15.728 |
|  | 7 | -6.2 | 2.932 | 7.079 |
|  | 8 | -6.1 | 14.851 | 17.605 |
|  | 9 | -6.1 | 3.477 | 5.951 |
|  | 10 | -6 | 2.863 | 3.240 |
|  | 11 | -5.9 | 28.300 | 30.258 |
|  | 12 | -5.9 | 2.596 | 3.441 |
|  | 13 | -5.9 | 13.351 | 14.702 |
|  | 14 | -5.9 | 18.373 | 20.939 |
|  | 15 | -5.8 | 13.442 | 15.644 |
|  | 16 | -5.8 | 13.480 | 16.027 |
|  | 17 | -5.8 | 12.483 | 14.464 |
|  | 18 | -5.8 | 16.878 | 18.860 |
|  | 19 | -5.8 | 14.408 | 16.816 |
|  | 20 | -5.6 | 13.503 | 15.984 |
| PTBP1-BIX02189 | 1 | -8.4 | 0.000 | 0.000 |
|  | 2 | -7.5 | 5.470 | 9.132 |
|  | 3 | -7.5 | 1.533 | 2.905 |
|  | 4 | -6.9 | 16.153 | 17.712 |
|  | 5 | -6.8 | 20.088 | 23.098 |
|  | 6 | -6.8 | 15.034 | 20.329 |
|  | 7 | -6.8 | 9.671 | 13.703 |
|  | 8 | -6.5 | 20.992 | 23.846 |
|  | 9 | -6.5 | 18.179 | 21.323 |
|  | 10 | -6.4 | 3.141 | 6.570 |
|  | 11 | -6.4 | 15.044 | 20.234 |
|  | 12 | -6.4 | 20.516 | 23.864 |
|  | 13 | -6.4 | 20.737 | 23.795 |
|  | 14 | -6.4 | 26.825 | 28.783 |
|  | 15 | -6.4 | 20.675 | 24.873 |
|  | 16 | -6.3 | 15.202 | 19.686 |
|  | 17 | -6.3 | 19.476 | 23.001 |
|  | 18 | -6.2 | 12.942 | 15.114 |
|  | 19 | -6.2 | 20.015 | 23.358 |
|  | 20 | -6.1 | 21.267 | 25.287 |
| PTBP1-CUDC-101 | 1 | -6.3 | 0.000 | 0.000 |
|  | 2 | -6.3 | 2.235 | 4.891 |
|  | 3 | -6.2 | 2.798 | 6.798 |
|  | 4 | -6 | 28.538 | 31.950 |
|  | 5 | -6 | 2.100 | 4.548 |
|  | 6 | -5.9 | 10.926 | 13.787 |
|  | 7 | -5.9 | 11.336 | 14.348 |
|  | 8 | -5.8 | 1.432 | 1.943 |
|  | 9 | -5.8 | 9.803 | 12.728 |
|  | 10 | -5.7 | 14.883 | 17.617 |
|  | 11 | -5.7 | 10.505 | 13.159 |
|  | 12 | -5.6 | 3.026 | 5.692 |
|  | 13 | -5.6 | 28.875 | 32.402 |
|  | 14 | -5.5 | 2.911 | 5.412 |
|  | 15 | -5.5 | 12.912 | 15.999 |
|  | 16 | -5.5 | 24.642 | 28.219 |
|  | 17 | -5.5 | 22.298 | 25.278 |
|  | 18 | -5.5 | 14.133 | 17.059 |
|  | 19 | -5.4 | 20.066 | 23.713 |
|  | 20 | -5.4 | 15.738 | 18.436 |
| PTBP1-PIK-93 | 1 | -7.3 | 0.000 | 0.000 |
|  | 2 | -7 | 24.089 | 25.988 |
|  | 3 | -6.6 | 25.535 | 27.397 |
|  | 4 | -6.5 | 24.840 | 26.684 |
|  | 5 | -6.3 | 4.641 | 6.665 |
|  | 6 | -6.3 | 24.665 | 26.461 |
|  | 7 | -6.3 | 1.691 | 2.456 |
|  | 8 | -6.3 | 24.238 | 26.415 |
|  | 9 | -6.2 | 28.424 | 29.871 |
|  | 10 | -6.2 | 6.328 | 7.670 |
|  | 11 | -6.2 | 28.798 | 30.616 |
|  | 12 | -6.1 | 30.442 | 32.197 |
|  | 13 | -6 | 14.869 | 17.276 |
|  | 14 | -5.9 | 15.478 | 18.028 |
|  | 15 | -5.9 | 16.236 | 18.189 |
|  | 16 | -5.8 | 3.836 | 5.042 |
|  | 17 | -5.7 | 5.289 | 7.028 |
|  | 18 | -5.7 | 15.079 | 17.433 |
|  | 19 | -5.6 | 24.208 | 26.391 |
|  | 20 | -5.6 | 7.751 | 12.439 |
| PTBP1-THZ-2-102-1 | 1 | -8.2 | 0.000 | 0.000 |
|  | 2 | -8.2 | 2.473 | 4.053 |
|  | 3 | -7.9 | 2.682 | 6.367 |
|  | 4 | -7.9 | 9.646 | 12.654 |
|  | 5 | -7.8 | 18.528 | 22.821 |
|  | 6 | -7.7 | 22.245 | 25.010 |
|  | 7 | -7.7 | 1.980 | 3.192 |
|  | 8 | -7.7 | 21.031 | 24.481 |
|  | 9 | -7.6 | 21.031 | 23.584 |
|  | 10 | -7.5 | 20.049 | 24.379 |
|  | 11 | -7.5 | 18.879 | 21.735 |
|  | 12 | -7.5 | 20.983 | 23.873 |
|  | 13 | -7.4 | 18.696 | 22.807 |
|  | 14 | -7.4 | 18.485 | 22.435 |
|  | 15 | -7.4 | 22.135 | 24.549 |
|  | 16 | -7.4 | 14.862 | 17.905 |
|  | 17 | -7.3 | 22.107 | 26.625 |
|  | 18 | -7.1 | 20.698 | 25.321 |
|  | 19 | -7 | 22.109 | 25.225 |
|  | 20 | -7 | 3.274 | 8.612 |
| PTBP1-Trametinib | 1 | -6.9 | 0.000 | 0.000 |
|  | 2 | -6.7 | 40.702 | 43.191 |
|  | 3 | -6.6 | 14.809 | 17.972 |
|  | 4 | -6.5 | 19.030 | 22.379 |
|  | 5 | -6.4 | 23.368 | 25.091 |
|  | 6 | -6.4 | 22.783 | 25.581 |
|  | 7 | -6.3 | 19.763 | 22.079 |
|  | 8 | -6.3 | 19.934 | 22.150 |
|  | 9 | -6.2 | 12.255 | 15.016 |
|  | 10 | -6.1 | 2.334 | 2.979 |
|  | 11 | -6.1 | 40.924 | 43.589 |
|  | 12 | -6 | 23.850 | 26.482 |
|  | 13 | -6 | 18.442 | 20.606 |
|  | 14 | -5.9 | 23.791 | 25.684 |
|  | 15 | -5.8 | 15.225 | 17.773 |
|  | 16 | -5.7 | 28.612 | 31.686 |
|  | 17 | -5.6 | 23.811 | 26.066 |
|  | 18 | -5.5 | 14.139 | 16.565 |
|  | 19 | -5.5 | 13.728 | 16.102 |
|  | 20 | -5.4 | 13.597 | 16.263 |
| PTBP1-17-AAG | 1 | -6.7 | 0.000 | 0.000 |
|  | 2 | -6.7 | 25.156 | 28.987 |
|  | 3 | -6.1 | 18.700 | 21.652 |
|  | 4 | -6.1 | 17.176 | 19.935 |
|  | 5 | -6 | 16.188 | 19.879 |
|  | 6 | -5.8 | 15.558 | 18.641 |
|  | 7 | -5.7 | 15.151 | 18.868 |
|  | 8 | -5.7 | 16.539 | 19.458 |
|  | 9 | -5.7 | 24.467 | 27.521 |
|  | 10 | -5.6 | 18.947 | 22.206 |
|  | 11 | -5.6 | 8.528 | 13.252 |
|  | 12 | -5.5 | 17.743 | 21.081 |
|  | 13 | -5.5 | 21.728 | 25.713 |
|  | 14 | -5.4 | 4.757 | 9.459 |
|  | 15 | -5.4 | 22.053 | 26.112 |
|  | 16 | -5.4 | 16.663 | 18.920 |
|  | 17 | -5.4 | 16.644 | 19.288 |
|  | 18 | -5.3 | 4.732 | 8.555 |
|  | 19 | -5.3 | 33.173 | 37.620 |
|  | 20 | -5.2 | 16.591 | 19.509 |
| PTBP1-PD-0325901 | 1 | -6 | 0.000 | 0.000 |
|  | 2 | -5.8 | 17.658 | 19.405 |
|  | 3 | -5.8 | 47.809 | 50.151 |
|  | 4 | -5.8 | 3.494 | 5.404 |
|  | 5 | -5.7 | 3.260 | 5.434 |
|  | 6 | -5.6 | 4.103 | 6.060 |
|  | 7 | -5.6 | 18.494 | 19.953 |
|  | 8 | -5.6 | 18.474 | 20.398 |
|  | 9 | -5.6 | 47.519 | 49.997 |
|  | 10 | -5.4 | 5.932 | 8.094 |
|  | 11 | -5.3 | 17.991 | 19.542 |
|  | 12 | -5.3 | 28.287 | 31.056 |
|  | 13 | -5.2 | 23.830 | 25.740 |
|  | 14 | -5.2 | 24.122 | 26.768 |
|  | 15 | -5.2 | 17.936 | 19.435 |
|  | 16 | -5.1 | 19.212 | 21.241 |
|  | 17 | -5.1 | 18.410 | 21.052 |
|  | 18 | -5 | 17.989 | 20.035 |
|  | 19 | -5 | 19.095 | 21.152 |
|  | 20 | -5 | 24.346 | 26.855 |
| NCAPG-AT-7519 | 1 | -6.3 | 0.000 | 0.000 |
|  | 2 | -6.2 | 73.563 | 76.222 |
|  | 3 | -6.1 | 3.116 | 5.238 |
|  | 4 | -6 | 63.187 | 64.830 |
|  | 5 | -5.9 | 3.206 | 5.528 |
|  | 6 | -5.7 | 71.645 | 73.976 |
|  | 7 | -5.7 | 56.966 | 58.795 |
|  | 8 | -5.7 | 67.826 | 69.827 |
|  | 9 | -5.7 | 91.150 | 92.970 |
|  | 10 | -5.7 | 37.843 | 41.350 |
|  | 11 | -5.7 | 46.823 | 49.176 |
|  | 12 | -5.6 | 58.611 | 60.505 |
|  | 13 | -5.5 | 20.855 | 22.814 |
|  | 14 | -5.5 | 86.859 | 88.309 |
|  | 15 | -5.4 | 16.811 | 19.406 |
|  | 16 | -5.3 | 3.582 | 6.824 |
|  | 17 | -5.3 | 17.026 | 20.259 |
|  | 18 | -4.8 | 29.599 | 32.827 |
|  | 19 | -4.8 | 91.651 | 93.072 |
|  | 20 | -4.7 | 15.697 | 18.130 |
| NCAPG-BIX02189 | 1 | -6.9 | 0.000 | 0.000 |
|  | 2 | -6.8 | 60.228 | 63.451 |
|  | 3 | -6.7 | 85.132 | 87.693 |
|  | 4 | -6.7 | 79.131 | 82.389 |
|  | 5 | -6.6 | 67.490 | 70.029 |
|  | 6 | -6.6 | 65.660 | 68.155 |
|  | 7 | -6.6 | 53.763 | 57.043 |
|  | 8 | -6.5 | 16.108 | 19.996 |
|  | 9 | -6.4 | 72.610 | 75.564 |
|  | 10 | -6.4 | 63.286 | 66.786 |
|  | 11 | -6.3 | 80.296 | 82.634 |
|  | 12 | -6.3 | 80.896 | 84.246 |
|  | 13 | -6.3 | 59.444 | 62.911 |
|  | 14 | -6.3 | 61.769 | 64.318 |
|  | 15 | -6.3 | 15.352 | 19.129 |
|  | 16 | -6.3 | 41.883 | 44.461 |
|  | 17 | -6.2 | 56.283 | 58.460 |
|  | 18 | -6.2 | 52.581 | 56.170 |
|  | 19 | -6.1 | 70.053 | 72.976 |
|  | 20 | -6.1 | 56.004 | 59.407 |
| NCAPG-CUDC-101 | 1 | -6.9 | 0.000 | 0.000 |
|  | 2 | -6.7 | 1.284 | 2.101 |
|  | 3 | -6.6 | 31.583 | 33.571 |
|  | 4 | -6.5 | 76.795 | 79.718 |
|  | 5 | -6.4 | 58.474 | 60.652 |
|  | 6 | -6.4 | 4.373 | 6.482 |
|  | 7 | -6.4 | 27.217 | 31.805 |
|  | 8 | -6.2 | 85.165 | 86.831 |
|  | 9 | -6.1 | 34.128 | 35.965 |
|  | 10 | -5.9 | 76.430 | 79.899 |
|  | 11 | -5.9 | 30.967 | 33.070 |
|  | 12 | -5.9 | 2.358 | 2.863 |
|  | 13 | -5.9 | 68.954 | 72.773 |
|  | 14 | -5.9 | 76.804 | 78.642 |
|  | 15 | -5.9 | 3.729 | 7.184 |
|  | 16 | -5.8 | 82.990 | 85.785 |
|  | 17 | -5.8 | 79.223 | 80.845 |
|  | 18 | -5.8 | 72.880 | 76.339 |
|  | 19 | -5.8 | 3.513 | 6.648 |
|  | 20 | -5.7 | 70.257 | 73.905 |
| NCAPG-PIK-93 | 1 | -6.9 | 0.000 | 0.000 |
|  | 2 | -6.4 | 1.998 | 2.241 |
|  | 3 | -6.3 | 3.939 | 7.288 |
|  | 4 | -6.2 | 3.414 | 7.638 |
|  | 5 | -6.2 | 3.081 | 7.132 |
|  | 6 | -6.1 | 80.344 | 81.567 |
|  | 7 | -5.9 | 1.934 | 2.500 |
|  | 8 | -5.7 | 2.977 | 7.347 |
|  | 9 | -5.7 | 3.463 | 4.304 |
|  | 10 | -5.6 | 5.038 | 7.185 |
|  | 11 | -5.6 | 6.182 | 9.748 |
|  | 12 | -5.6 | 34.081 | 37.504 |
|  | 13 | -5.5 | 28.859 | 30.502 |
|  | 14 | -5.5 | 3.247 | 7.845 |
|  | 15 | -5.5 | 83.161 | 85.920 |
|  | 16 | -5.3 | 66.619 | 68.467 |
|  | 17 | -5.2 | 13.914 | 17.104 |
|  | 18 | -5.1 | 82.709 | 85.399 |
|  | 19 | -5 | 43.718 | 45.227 |
|  | 20 | -5 | 43.561 | 45.531 |
| NCAPG-THZ-2-102-1 | 1 | -8.2 | 0.000 | 0.000 |
|  | 2 | -7.5 | 95.782 | 98.940 |
|  | 3 | -7.4 | 84.067 | 86.767 |
|  | 4 | -7.4 | 83.830 | 86.569 |
|  | 5 | -7.3 | 78.713 | 82.075 |
|  | 6 | -7.3 | 105.557 | 108.177 |
|  | 7 | -7.3 | 92.687 | 95.003 |
|  | 8 | -7.2 | 78.686 | 82.010 |
|  | 9 | -7.1 | 83.301 | 86.810 |
|  | 10 | -7.1 | 76.174 | 78.959 |
|  | 11 | -7 | 83.561 | 86.580 |
|  | 12 | -7 | 83.446 | 86.236 |
|  | 13 | -6.9 | 106.787 | 109.570 |
|  | 14 | -6.8 | 53.922 | 57.078 |
|  | 15 | -6.8 | 89.616 | 94.426 |
|  | 16 | -6.7 | 94.979 | 98.561 |
|  | 17 | -6.6 | 90.954 | 94.943 |
|  | 18 | -6.5 | 97.648 | 100.126 |
|  | 19 | -6.5 | 72.708 | 75.167 |
|  | 20 | -6.4 | 83.786 | 86.417 |
| NCAPG-Trametinib | 1 | -6.6 | 0.000 | 0.000 |
|  | 2 | -6.5 | 52.505 | 55.777 |
|  | 3 | -6.2 | 51.745 | 53.853 |
|  | 4 | -6.2 | 54.952 | 58.517 |
|  | 5 | -6.2 | 35.527 | 38.258 |
|  | 6 | -6.2 | 48.213 | 51.844 |
|  | 7 | -6 | 33.028 | 36.318 |
|  | 8 | -6 | 58.935 | 61.410 |
|  | 9 | -6 | 24.309 | 26.743 |
|  | 10 | -6 | 54.450 | 57.184 |
|  | 11 | -5.8 | 59.371 | 62.247 |
|  | 12 | -5.8 | 64.816 | 68.613 |
|  | 13 | -5.8 | 54.925 | 57.877 |
|  | 14 | -5.6 | 55.058 | 58.313 |
|  | 15 | -5.6 | 59.529 | 63.640 |
|  | 16 | -5.6 | 37.561 | 40.601 |
|  | 17 | -5.6 | 58.715 | 62.760 |
|  | 18 | -5.6 | 24.308 | 26.940 |
|  | 19 | -5.6 | 64.332 | 67.061 |
|  | 20 | -5.6 | 61.550 | 65.626 |
| NCAPG-17-AAG | 1 | -7 | 0.000 | 0.000 |
|  | 2 | -6.6 | 76.252 | 81.660 |
|  | 3 | -6.2 | 46.910 | 52.123 |
|  | 4 | -6.1 | 31.572 | 35.749 |
|  | 5 | -6.1 | 30.801 | 34.688 |
|  | 6 | -6 | 20.989 | 26.017 |
|  | 7 | -6 | 23.200 | 28.219 |
|  | 8 | -5.8 | 2.098 | 2.921 |
|  | 9 | -5.8 | 31.980 | 36.353 |
|  | 10 | -5.7 | 57.475 | 61.333 |
|  | 11 | -5.7 | 45.780 | 50.742 |
|  | 12 | -5.7 | 30.392 | 34.256 |
|  | 13 | -5.6 | 28.542 | 33.810 |
|  | 14 | -5.6 | 73.346 | 77.193 |
|  | 15 | -5.5 | 48.673 | 53.812 |
|  | 16 | -5.5 | 30.497 | 33.564 |
|  | 17 | -5.5 | 53.184 | 58.695 |
|  | 18 | -5.4 | 71.190 | 73.735 |
|  | 19 | -5.4 | 11.900 | 14.481 |
|  | 20 | -5.4 | 28.039 | 31.699 |
| NCAPG-PD-0325901 | 1 | -5.9 | 0.000 | 0.000 |
|  | 2 | -5.7 | 45.909 | 47.347 |
|  | 3 | -5.7 | 71.137 | 72.044 |
|  | 4 | -5.5 | 71.321 | 72.736 |
|  | 5 | -5.3 | 72.043 | 73.766 |
|  | 6 | -5.3 | 71.661 | 73.347 |
|  | 7 | -5.3 | 71.482 | 73.733 |
|  | 8 | -5.3 | 16.481 | 18.571 |
|  | 9 | -5.2 | 44.649 | 46.923 |
|  | 10 | -5.1 | 101.626 | 103.430 |
|  | 11 | -5.1 | 71.516 | 73.730 |
|  | 12 | -5 | 113.952 | 116.908 |
|  | 13 | -5 | 98.056 | 100.671 |
|  | 14 | -4.9 | 63.710 | 65.193 |
|  | 15 | -4.9 | 72.217 | 74.676 |
|  | 16 | -4.6 | 55.909 | 58.826 |
|  | 17 | -4.6 | 23.043 | 25.146 |
|  | 18 | -4.6 | 44.302 | 47.368 |
|  | 19 | -4.5 | 54.351 | 57.205 |
|  | 20 | -4.4 | 65.143 | 68.376 |
| BMP2-AT-7519 | 1 | -6 | 0.000 | 0.000 |
|  | 2 | -5.9 | 19.277 | 20.903 |
|  | 3 | -5.9 | 3.382 | 5.525 |
|  | 4 | -5.9 | 2.160 | 4.394 |
|  | 5 | -5.8 | 13.178 | 14.827 |
|  | 6 | -5.8 | 19.934 | 21.114 |
|  | 7 | -5.7 | 3.472 | 6.225 |
|  | 8 | -5.7 | 20.188 | 22.576 |
|  | 9 | -5.7 | 3.576 | 6.517 |
|  | 10 | -5.6 | 2.155 | 4.896 |
|  | 11 | -5.6 | 13.286 | 14.688 |
|  | 12 | -5.6 | 15.574 | 17.793 |
|  | 13 | -5.5 | 2.637 | 3.753 |
|  | 14 | -5.5 | 3.279 | 4.785 |
|  | 15 | -5.4 | 3.018 | 6.958 |
|  | 16 | -5.4 | 19.049 | 20.525 |
|  | 17 | -5.3 | 19.147 | 21.794 |
|  | 18 | -5.3 | 16.320 | 18.601 |
|  | 19 | -5.2 | 14.052 | 15.955 |
|  | 20 | -5.2 | 19.106 | 22.190 |
| BMP2-BIX02189 | 1 | -6.5 | 0.000 | 0.000 |
|  | 2 | -6.5 | 13.644 | 17.299 |
|  | 3 | -6.4 | 10.207 | 13.853 |
|  | 4 | -6.3 | 12.125 | 15.633 |
|  | 5 | -6.2 | 1.500 | 2.784 |
|  | 6 | -6.2 | 12.781 | 16.462 |
|  | 7 | -6.2 | 5.032 | 9.081 |
|  | 8 | -6.1 | 22.257 | 26.601 |
|  | 9 | -6.1 | 9.944 | 14.263 |
|  | 10 | -6.1 | 13.103 | 16.290 |
|  | 11 | -6 | 11.419 | 14.902 |
|  | 12 | -5.9 | 12.221 | 15.823 |
|  | 13 | -5.8 | 15.239 | 16.978 |
|  | 14 | -5.8 | 9.103 | 11.726 |
|  | 15 | -5.8 | 4.634 | 6.287 |
|  | 16 | -5.7 | 10.640 | 14.463 |
|  | 17 | -5.7 | 21.870 | 25.573 |
|  | 18 | -5.7 | 13.941 | 17.103 |
|  | 19 | -5.6 | 15.465 | 17.304 |
|  | 20 | -5.5 | 23.540 | 26.567 |
| BMP2-CUDC-101 | 1 | -6.6 | 0.000 | 0.000 |
|  | 2 | -6.4 | 3.890 | 6.181 |
|  | 3 | -6.2 | 17.739 | 21.399 |
|  | 4 | -6.2 | 3.713 | 7.043 |
|  | 5 | -6.2 | 3.878 | 7.372 |
|  | 6 | -6.1 | 3.267 | 6.185 |
|  | 7 | -6 | 16.881 | 20.598 |
|  | 8 | -6 | 12.534 | 14.638 |
|  | 9 | -6 | 12.945 | 15.575 |
|  | 10 | -5.9 | 3.367 | 4.095 |
|  | 11 | -5.9 | 15.506 | 18.932 |
|  | 12 | -5.9 | 2.286 | 5.485 |
|  | 13 | -5.8 | 17.430 | 21.407 |
|  | 14 | -5.8 | 15.981 | 19.176 |
|  | 15 | -5.7 | 14.472 | 17.510 |
|  | 16 | -5.7 | 14.138 | 17.584 |
|  | 17 | -5.7 | 16.193 | 19.767 |
|  | 18 | -5.6 | 5.381 | 8.373 |
|  | 19 | -5.5 | 2.215 | 5.420 |
|  | 20 | -5.5 | 3.778 | 5.047 |
| BMP2-PIK-93 | 1 | -6.4 | 0.000 | 0.000 |
|  | 2 | -6 | 33.851 | 36.646 |
|  | 3 | -6 | 4.250 | 7.354 |
|  | 4 | -5.9 | 18.840 | 21.373 |
|  | 5 | -5.8 | 19.018 | 21.365 |
|  | 6 | -5.8 | 4.577 | 7.386 |
|  | 7 | -5.8 | 19.295 | 21.942 |
|  | 8 | -5.8 | 2.970 | 7.284 |
|  | 9 | -5.7 | 2.721 | 3.404 |
|  | 10 | -5.7 | 2.789 | 7.552 |
|  | 11 | -5.7 | 31.592 | 33.822 |
|  | 12 | -5.6 | 19.385 | 21.199 |
|  | 13 | -5.6 | 3.070 | 4.261 |
|  | 14 | -5.6 | 3.040 | 7.734 |
|  | 15 | -5.5 | 19.263 | 20.785 |
|  | 16 | -5.5 | 19.163 | 21.320 |
|  | 17 | -5.5 | 17.666 | 19.458 |
|  | 18 | -5.4 | 4.478 | 7.604 |
|  | 19 | -5.2 | 17.556 | 20.010 |
|  | 20 | -5.1 | 19.761 | 22.333 |
| BMP2-THZ-2-102-1 | 1 | -7.5 | 0.000 | 0.000 |
|  | 2 | -7.1 | 2.296 | 4.077 |
|  | 3 | -7 | 13.914 | 16.991 |
|  | 4 | -7 | 4.064 | 6.317 |
|  | 5 | -7 | 2.889 | 6.543 |
|  | 6 | -6.8 | 13.659 | 16.206 |
|  | 7 | -6.7 | 3.700 | 6.767 |
|  | 8 | -6.7 | 18.921 | 22.661 |
|  | 9 | -6.6 | 16.871 | 22.365 |
|  | 10 | -6.6 | 15.307 | 17.246 |
|  | 11 | -6.5 | 2.055 | 3.899 |
|  | 12 | -6.5 | 14.473 | 16.943 |
|  | 13 | -6.5 | 14.833 | 17.425 |
|  | 14 | -6.5 | 17.336 | 20.008 |
|  | 15 | -6.5 | 18.257 | 21.237 |
|  | 16 | -6.4 | 14.208 | 17.029 |
|  | 17 | -6.4 | 19.869 | 24.124 |
|  | 18 | -6.3 | 18.482 | 21.634 |
|  | 19 | -6.3 | 2.568 | 4.253 |
|  | 20 | -6.2 | 13.700 | 16.117 |
| BMP2-Trametinib | 1 | -7 | 0.000 | 0.000 |
|  | 2 | -6.5 | 14.455 | 17.558 |
|  | 3 | -6.4 | 15.627 | 19.595 |
|  | 4 | -6.4 | 16.271 | 18.060 |
|  | 5 | -6.4 | 15.504 | 18.474 |
|  | 6 | -6.3 | 14.499 | 19.730 |
|  | 7 | -6.3 | 15.185 | 18.316 |
|  | 8 | -6.3 | 14.515 | 16.898 |
|  | 9 | -6.2 | 17.055 | 20.028 |
|  | 10 | -6.2 | 17.079 | 20.410 |
|  | 11 | -6.2 | 14.743 | 17.486 |
|  | 12 | -6.2 | 14.553 | 18.387 |
|  | 13 | -6.1 | 14.695 | 16.515 |
|  | 14 | -6.1 | 2.314 | 3.007 |
|  | 15 | -6.1 | 1.390 | 2.106 |
|  | 16 | -6 | 14.510 | 16.211 |
|  | 17 | -6 | 15.647 | 18.286 |
|  | 18 | -6 | 14.081 | 18.091 |
|  | 19 | -5.8 | 3.807 | 7.557 |
|  | 20 | -5.8 | 14.696 | 18.045 |
| BMP2-17-AAG | 1 | -5.8 | 0.000 | 0.000 |
|  | 2 | -5.7 | 13.008 | 18.414 |
|  | 3 | -5.5 | 13.064 | 17.708 |
|  | 4 | -5.5 | 2.584 | 8.473 |
|  | 5 | -5.4 | 12.157 | 17.018 |
|  | 6 | -5.3 | 14.555 | 18.634 |
|  | 7 | -5.3 | 11.070 | 15.558 |
|  | 8 | -5.3 | 18.811 | 23.703 |
|  | 9 | -5.3 | 18.114 | 21.536 |
|  | 10 | -5.3 | 27.452 | 32.415 |
|  | 11 | -5.2 | 17.655 | 21.316 |
|  | 12 | -5.2 | 4.833 | 9.920 |
|  | 13 | -5.2 | 17.761 | 21.629 |
|  | 14 | -5.2 | 12.630 | 18.085 |
|  | 15 | -5.1 | 2.938 | 5.156 |
|  | 16 | -5.1 | 11.861 | 17.121 |
|  | 17 | -5.1 | 14.407 | 19.368 |
|  | 18 | -5 | 8.129 | 14.040 |
|  | 19 | -5 | 20.745 | 24.211 |
|  | 20 | -4.9 | 14.453 | 18.508 |
| BMP2-PD-0325901 | 1 | -5.3 | 0.000 | 0.000 |
|  | 2 | -5.2 | 13.674 | 15.695 |
|  | 3 | -5.2 | 21.813 | 23.775 |
|  | 4 | -5.2 | 21.083 | 22.841 |
|  | 5 | -5.1 | 4.513 | 6.689 |
|  | 6 | -5.1 | 21.456 | 23.569 |
|  | 7 | -5.1 | 5.413 | 7.567 |
|  | 8 | -5.1 | 4.295 | 6.082 |
|  | 9 | -5 | 22.538 | 24.434 |
|  | 10 | -5 | 13.273 | 14.847 |
|  | 11 | -4.9 | 12.998 | 14.825 |
|  | 12 | -4.9 | 12.897 | 14.314 |
|  | 13 | -4.8 | 13.611 | 15.556 |
|  | 14 | -4.7 | 14.219 | 16.012 |
|  | 15 | -4.7 | 22.426 | 24.281 |
|  | 16 | -4.7 | 19.994 | 21.507 |
|  | 17 | -4.6 | 14.470 | 17.561 |
|  | 18 | -4.6 | 22.844 | 24.497 |
|  | 19 | -4.6 | 13.431 | 15.328 |
|  | 20 | -4.6 | 5.025 | 7.214 |
| PDE2A-AT-7519 | 1 | -8.8 | 0.000 | 0.000 |
|  | 2 | -8.7 | 3.038 | 3.973 |
|  | 3 | -8.7 | 2.996 | 4.279 |
|  | 4 | -8.5 | 2.775 | 5.029 |
|  | 5 | -8.3 | 3.107 | 4.407 |
|  | 6 | -8.3 | 2.043 | 4.819 |
|  | 7 | -8.2 | 4.442 | 6.319 |
|  | 8 | -8 | 2.968 | 4.189 |
|  | 9 | -7.7 | 3.211 | 7.316 |
|  | 10 | -7.6 | 3.919 | 6.991 |
|  | 11 | -7.3 | 4.490 | 6.540 |
|  | 12 | -7.2 | 4.298 | 6.712 |
|  | 13 | -7.1 | 5.065 | 7.487 |
|  | 14 | -6.8 | 4.744 | 7.003 |
|  | 15 | -6.6 | 30.767 | 32.829 |
|  | 16 | -6.6 | 29.214 | 32.462 |
|  | 17 | -6.6 | 5.428 | 7.259 |
|  | 18 | -6.4 | 32.488 | 33.707 |
|  | 19 | -6.4 | 13.108 | 14.926 |
|  | 20 | -6.4 | 12.809 | 15.071 |
| PDE2A-BIX02189 | 1 | -10 | 0.000 | 0.000 |
|  | 2 | -9.9 | 4.290 | 7.310 |
|  | 3 | -9.7 | 3.758 | 6.094 |
|  | 4 | -9.5 | 4.051 | 6.912 |
|  | 5 | -9.5 | 2.302 | 3.186 |
|  | 6 | -9.3 | 4.703 | 7.484 |
|  | 7 | -9.3 | 1.785 | 2.733 |
|  | 8 | -9.2 | 2.922 | 4.871 |
|  | 9 | -9.1 | 1.858 | 2.440 |
|  | 10 | -8.9 | 2.670 | 8.857 |
|  | 11 | -8.4 | 3.816 | 5.461 |
|  | 12 | -8.2 | 2.886 | 4.650 |
|  | 13 | -8 | 2.943 | 4.023 |
|  | 14 | -8 | 6.354 | 9.757 |
|  | 15 | -7.7 | 2.752 | 3.977 |
|  | 16 | -7.7 | 8.138 | 10.618 |
|  | 17 | -7.6 | 10.502 | 12.470 |
|  | 18 | -7.6 | 5.476 | 8.140 |
|  | 19 | -7.4 | 15.550 | 19.354 |
|  | 20 | -7.4 | 15.461 | 17.514 |
| PDE2A-CUDC-101 | 1 | -9 | 0.000 | 0.000 |
|  | 2 | -8.6 | 3.013 | 6.684 |
|  | 3 | -7.9 | 2.927 | 5.574 |
|  | 4 | -7.9 | 3.244 | 7.000 |
|  | 5 | -7.9 | 2.782 | 5.089 |
|  | 6 | -7.8 | 2.568 | 5.179 |
|  | 7 | -7.8 | 2.471 | 4.636 |
|  | 8 | -7.7 | 2.756 | 4.348 |
|  | 9 | -7.6 | 2.680 | 4.554 |
|  | 10 | -7.6 | 3.292 | 5.640 |
|  | 11 | -7.6 | 3.115 | 5.722 |
|  | 12 | -7.4 | 2.786 | 5.431 |
|  | 13 | -7.3 | 2.158 | 4.154 |
|  | 14 | -7.3 | 3.170 | 4.965 |
|  | 15 | -7.2 | 3.316 | 7.301 |
|  | 16 | -7.2 | 3.411 | 5.394 |
|  | 17 | -7.1 | 3.852 | 7.135 |
|  | 18 | -7 | 3.123 | 5.880 |
|  | 19 | -7 | 4.156 | 8.141 |
|  | 20 | -6.7 | 2.712 | 5.586 |
| PDE2A-PIK-93 | 1 | -8 | 0.000 | 0.000 |
|  | 2 | -7.7 | 4.959 | 7.922 |
|  | 3 | -7.7 | 2.132 | 2.487 |
|  | 4 | -7.7 | 3.677 | 5.116 |
|  | 5 | -7.5 | 5.320 | 7.689 |
|  | 6 | -7.4 | 5.380 | 6.736 |
|  | 7 | -7.1 | 4.866 | 7.897 |
|  | 8 | -7.1 | 4.287 | 8.501 |
|  | 9 | -6.7 | 8.169 | 11.945 |
|  | 10 | -6.7 | 4.475 | 5.880 |
|  | 11 | -6.4 | 4.250 | 9.701 |
|  | 12 | -6.4 | 3.806 | 8.566 |
|  | 13 | -6.4 | 4.593 | 7.838 |
|  | 14 | -6.3 | 4.241 | 6.254 |
|  | 15 | -6.3 | 33.149 | 34.623 |
|  | 16 | -6.2 | 4.233 | 8.723 |
|  | 17 | -6.2 | 3.947 | 4.918 |
|  | 18 | -6.1 | 4.731 | 7.496 |
|  | 19 | -6.1 | 7.014 | 8.400 |
|  | 20 | -6.1 | 5.520 | 7.093 |
| PDE2A-THZ-2-102-1 | 1 | -10.8 | 0.000 | 0.000 |
|  | 2 | -10 | 1.917 | 2.864 |
|  | 3 | -9.6 | 1.995 | 4.959 |
|  | 4 | -9.6 | 1.556 | 2.332 |
|  | 5 | -9.1 | 2.235 | 4.296 |
|  | 6 | -9 | 2.881 | 5.157 |
|  | 7 | -8.5 | 3.524 | 5.170 |
|  | 8 | -8.4 | 3.241 | 4.922 |
|  | 9 | -8.4 | 27.319 | 30.113 |
|  | 10 | -8.3 | 10.690 | 14.380 |
|  | 11 | -7.9 | 9.057 | 14.088 |
|  | 12 | -7.9 | 9.014 | 13.991 |
|  | 13 | -7.9 | 6.017 | 12.257 |
|  | 14 | -7.8 | 15.610 | 17.851 |
|  | 15 | -7.7 | 12.837 | 18.380 |
|  | 16 | -7.7 | 12.240 | 14.950 |
|  | 17 | -7.7 | 17.394 | 20.255 |
|  | 18 | -7.5 | 23.148 | 28.372 |
|  | 19 | -7.4 | 10.370 | 14.689 |
|  | 20 | -7.3 | 10.289 | 13.371 |
| PDE2A-Trametinib | 1 | -7.3 | 0.000 | 0.000 |
|  | 2 | -6.7 | 8.222 | 11.826 |
|  | 3 | -6.6 | 19.958 | 22.506 |
|  | 4 | -6.4 | 3.264 | 4.754 |
|  | 5 | -6.3 | 9.903 | 12.939 |
|  | 6 | -6.3 | 20.448 | 23.822 |
|  | 7 | -6.2 | 11.120 | 14.180 |
|  | 8 | -6.1 | 40.272 | 43.033 |
|  | 9 | -6.1 | 23.773 | 27.265 |
|  | 10 | -5.9 | 19.481 | 22.314 |
|  | 11 | -5.9 | 11.330 | 14.273 |
|  | 12 | -5.9 | 26.719 | 30.862 |
|  | 13 | -5.8 | 27.336 | 30.700 |
|  | 14 | -5.8 | 12.371 | 15.469 |
|  | 15 | -5.8 | 19.910 | 22.569 |
|  | 16 | -5.7 | 19.672 | 22.605 |
|  | 17 | -5.7 | 18.452 | 21.181 |
|  | 18 | -5.7 | 11.657 | 14.997 |
|  | 19 | -5.6 | 24.940 | 30.954 |
|  | 20 | -5.6 | 18.736 | 21.913 |
| PDE2A-17-AAG | 1 | -6.1 | 0.000 | 0.000 |
|  | 2 | -6.1 | 12.606 | 17.731 |
|  | 3 | -6 | 24.848 | 30.072 |
|  | 4 | -6 | 3.378 | 6.341 |
|  | 5 | -6 | 35.115 | 38.895 |
|  | 6 | -5.9 | 19.353 | 22.477 |
|  | 7 | -5.8 | 27.195 | 31.422 |
|  | 8 | -5.7 | 2.991 | 8.255 |
|  | 9 | -5.6 | 2.971 | 6.602 |
|  | 10 | -5.5 | 13.603 | 16.344 |
|  | 11 | -5.5 | 3.369 | 6.943 |
|  | 12 | -5.4 | 19.698 | 25.015 |
|  | 13 | -5.3 | 30.599 | 33.831 |
|  | 14 | -5.3 | 32.627 | 34.813 |
|  | 15 | -5.3 | 3.640 | 7.428 |
|  | 16 | -5.3 | 30.266 | 32.932 |
|  | 17 | -5.3 | 17.313 | 21.167 |
|  | 18 | -5.2 | 37.911 | 43.017 |
|  | 19 | -5.2 | 18.544 | 22.119 |
|  | 20 | -5.2 | 31.211 | 34.826 |
| PDE2A-PD-0325901 | 1 | -7.1 | 0.000 | 0.000 |
|  | 2 | -6.8 | 4.237 | 6.612 |
|  | 3 | -6.7 | 6.842 | 9.345 |
|  | 4 | -6.6 | 1.872 | 3.462 |
|  | 5 | -6.4 | 4.141 | 5.971 |
|  | 6 | -6.3 | 3.584 | 5.074 |
|  | 7 | -6.3 | 4.078 | 4.935 |
|  | 8 | -6.2 | 3.436 | 5.569 |
|  | 9 | -6 | 31.299 | 32.616 |
|  | 10 | -5.8 | 9.043 | 11.833 |
|  | 11 | -5.8 | 11.265 | 13.500 |
|  | 12 | -5.7 | 30.328 | 31.863 |
|  | 13 | -5.7 | 8.820 | 11.735 |
|  | 14 | -5.6 | 4.325 | 6.430 |
|  | 15 | -5.4 | 18.277 | 19.354 |
|  | 16 | -5.4 | 30.413 | 31.776 |
|  | 17 | -5.3 | 8.408 | 11.245 |
|  | 18 | -5.2 | 22.792 | 24.773 |
|  | 19 | -5.1 | 28.372 | 30.830 |
|  | 20 | -5 | 33.256 | 34.498 |
| IF130-AT-7519 | 1 | -6.6 | 0.000 | 0.000 |
|  | 2 | -6.2 | 24.961 | 27.646 |
|  | 3 | -6.2 | 24.307 | 26.027 |
|  | 4 | -6.2 | 24.212 | 26.194 |
|  | 5 | -6.1 | 24.953 | 27.392 |
|  | 6 | -6.1 | 12.953 | 15.836 |
|  | 7 | -5.9 | 25.050 | 26.327 |
|  | 8 | -5.8 | 20.600 | 22.411 |
|  | 9 | -5.8 | 25.028 | 28.702 |
|  | 10 | -5.7 | 20.141 | 21.681 |
|  | 11 | -5.7 | 19.973 | 21.395 |
|  | 12 | -5.6 | 19.216 | 23.141 |
|  | 13 | -5.6 | 26.060 | 27.573 |
|  | 14 | -5.6 | 3.315 | 4.681 |
|  | 15 | -5.6 | 19.678 | 20.835 |
|  | 16 | -5.5 | 25.959 | 27.875 |
|  | 17 | -5.5 | 3.162 | 4.580 |
|  | 18 | -5.4 | 20.099 | 21.238 |
|  | 19 | -5.4 | 17.225 | 20.334 |
|  | 20 | -5.3 | 24.559 | 25.833 |
| IF130-BIX02189 | 1 | -6.8 | 0.000 | 0.000 |
|  | 2 | -6.5 | 3.904 | 7.998 |
|  | 3 | -6.3 | 10.129 | 13.874 |
|  | 4 | -6.3 | 5.633 | 11.156 |
|  | 5 | -6.1 | 2.046 | 2.532 |
|  | 6 | -6.1 | 9.882 | 12.832 |
|  | 7 | -6.1 | 9.595 | 14.339 |
|  | 8 | -6.1 | 9.997 | 13.378 |
|  | 9 | -6 | 2.055 | 2.832 |
|  | 10 | -6 | 1.461 | 3.095 |
|  | 11 | -5.9 | 21.334 | 24.134 |
|  | 12 | -5.9 | 3.540 | 8.784 |
|  | 13 | -5.9 | 10.735 | 14.556 |
|  | 14 | -5.8 | 6.144 | 11.616 |
|  | 15 | -5.8 | 24.502 | 27.209 |
|  | 16 | -5.8 | 36.148 | 39.137 |
|  | 17 | -5.8 | 17.191 | 20.420 |
|  | 18 | -5.8 | 2.914 | 9.682 |
|  | 19 | -5.6 | 27.573 | 30.530 |
|  | 20 | -5.6 | 14.036 | 17.218 |
| IF130-CUDC-101 | 1 | -6.6 | 0.000 | 0.000 |
|  | 2 | -6.4 | 2.412 | 5.721 |
|  | 3 | -6.2 | 2.063 | 5.425 |
|  | 4 | -6.2 | 2.591 | 5.738 |
|  | 5 | -6 | 13.163 | 16.373 |
|  | 6 | -6 | 1.832 | 2.841 |
|  | 7 | -6 | 2.914 | 7.858 |
|  | 8 | -6 | 2.315 | 3.707 |
|  | 9 | -6 | 3.268 | 7.943 |
|  | 10 | -6 | 2.688 | 6.002 |
|  | 11 | -5.9 | 20.731 | 23.357 |
|  | 12 | -5.7 | 1.885 | 3.510 |
|  | 13 | -5.7 | 21.068 | 23.205 |
|  | 14 | -5.7 | 14.060 | 17.428 |
|  | 15 | -5.6 | 16.572 | 19.988 |
|  | 16 | -5.6 | 2.239 | 4.088 |
|  | 17 | -5.6 | 12.529 | 17.616 |
|  | 18 | -5.6 | 3.497 | 5.841 |
|  | 19 | -5.5 | 17.693 | 21.432 |
|  | 20 | -5.4 | 13.168 | 16.774 |
| IF130-PIK-93 | 1 | -6.4 | 0.000 | 0.000 |
|  | 2 | -6.1 | 3.636 | 8.139 |
|  | 3 | -6 | 1.700 | 2.070 |
|  | 4 | -5.8 | 4.168 | 8.244 |
|  | 5 | -5.6 | 32.117 | 33.018 |
|  | 6 | -5.5 | 5.051 | 7.071 |
|  | 7 | -5.5 | 3.984 | 8.154 |
|  | 8 | -5.5 | 20.296 | 21.829 |
|  | 9 | -5.4 | 26.731 | 30.839 |
|  | 10 | -5.3 | 15.447 | 17.224 |
|  | 11 | -5.2 | 1.962 | 2.406 |
|  | 12 | -5.2 | 19.036 | 22.855 |
|  | 13 | -5.1 | 4.906 | 7.515 |
|  | 14 | -5.1 | 15.072 | 19.040 |
|  | 15 | -5.1 | 18.912 | 22.068 |
|  | 16 | -5.1 | 18.912 | 20.357 |
|  | 17 | -5.1 | 22.990 | 24.559 |
|  | 18 | -5.1 | 35.534 | 37.681 |
|  | 19 | -5 | 27.955 | 30.073 |
|  | 20 | -5 | 27.569 | 30.026 |
| IF130-THZ-2-102-1 | 1 | -7.3 | 0.000 | 0.000 |
|  | 2 | -7.1 | 14.509 | 18.626 |
|  | 3 | -7 | 13.486 | 16.633 |
|  | 4 | -6.9 | 16.283 | 20.809 |
|  | 5 | -6.9 | 13.055 | 16.247 |
|  | 6 | -6.8 | 12.917 | 16.260 |
|  | 7 | -6.8 | 13.717 | 17.707 |
|  | 8 | -6.6 | 3.326 | 8.183 |
|  | 9 | -6.6 | 3.247 | 7.038 |
|  | 10 | -6.5 | 25.442 | 29.377 |
|  | 11 | -6.5 | 21.141 | 23.881 |
|  | 12 | -6.5 | 19.533 | 22.396 |
|  | 13 | -6.5 | 3.839 | 8.596 |
|  | 14 | -6.4 | 2.877 | 5.464 |
|  | 15 | -6.4 | 21.266 | 24.173 |
|  | 16 | -6.4 | 23.203 | 25.671 |
|  | 17 | -6.3 | 3.398 | 7.530 |
|  | 18 | -6.2 | 24.983 | 27.917 |
|  | 19 | -6.2 | 10.976 | 14.758 |
|  | 20 | -6 | 3.044 | 8.386 |
| IF130-Trametinib | 1 | -6.3 | 0.000 | 0.000 |
|  | 2 | -6.2 | 2.328 | 3.965 |
|  | 3 | -6.1 | 9.592 | 13.331 |
|  | 4 | -6 | 25.893 | 29.702 |
|  | 5 | -6 | 21.081 | 23.575 |
|  | 6 | -5.9 | 27.692 | 30.797 |
|  | 7 | -5.8 | 2.454 | 3.668 |
|  | 8 | -5.7 | 17.265 | 19.860 |
|  | 9 | -5.6 | 17.477 | 19.451 |
|  | 10 | -5.6 | 3.579 | 6.320 |
|  | 11 | -5.6 | 3.531 | 6.302 |
|  | 12 | -5.5 | 30.959 | 34.474 |
|  | 13 | -5.4 | 28.388 | 31.365 |
|  | 14 | -5.4 | 17.247 | 19.608 |
|  | 15 | -5.4 | 16.774 | 18.931 |
|  | 16 | -5.4 | 39.677 | 43.005 |
|  | 17 | -5.3 | 27.660 | 29.956 |
|  | 18 | -5.3 | 21.015 | 23.060 |
|  | 19 | -5.2 | 10.383 | 14.135 |
|  | 20 | -5.2 | 27.566 | 29.467 |
| IF130-17-AAG | 1 | -7.1 | 0.000 | 0.000 |
|  | 2 | -6.5 | 14.656 | 17.987 |
|  | 3 | -6.2 | 2.246 | 6.006 |
|  | 4 | -6.2 | 14.437 | 17.552 |
|  | 5 | -6.2 | 2.991 | 8.440 |
|  | 6 | -6.2 | 2.364 | 7.126 |
|  | 7 | -6.1 | 15.995 | 19.837 |
|  | 8 | -5.9 | 3.267 | 8.203 |
|  | 9 | -5.6 | 25.865 | 29.470 |
|  | 10 | -5.6 | 26.625 | 29.360 |
|  | 11 | -5.6 | 12.551 | 16.119 |
|  | 12 | -5.6 | 2.535 | 6.583 |
|  | 13 | -5.5 | 26.906 | 29.823 |
|  | 14 | -5.5 | 17.722 | 22.385 |
|  | 15 | -5.5 | 30.667 | 34.310 |
|  | 16 | -5.5 | 27.376 | 30.662 |
|  | 17 | -5.4 | 25.042 | 28.671 |
|  | 18 | -5.3 | 3.104 | 4.070 |
|  | 19 | -5.2 | 31.635 | 34.627 |
|  | 20 | -5.2 | 15.559 | 20.188 |
| IF130-PD-0325901 | 1 | -5.9 | 0.000 | 0.000 |
|  | 2 | -5.4 | 2.057 | 2.491 |
|  | 3 | -5.4 | 3.439 | 5.019 |
|  | 4 | -5.2 | 16.794 | 19.120 |
|  | 5 | -5.1 | 3.992 | 6.201 |
|  | 6 | -5.1 | 6.744 | 8.078 |
|  | 7 | -5 | 12.460 | 14.459 |
|  | 8 | -4.9 | 19.483 | 21.382 |
|  | 9 | -4.9 | 3.026 | 4.701 |
|  | 10 | -4.9 | 10.983 | 12.621 |
|  | 11 | -4.7 | 25.885 | 28.478 |
|  | 12 | -4.6 | 9.860 | 12.311 |
|  | 13 | -4.5 | 25.771 | 27.587 |
|  | 14 | -4.4 | 9.298 | 11.722 |
|  | 15 | -4.4 | 14.791 | 16.856 |
|  | 16 | -4.4 | 19.124 | 20.929 |
|  | 17 | -4.4 | 12.182 | 14.545 |
|  | 18 | -4.4 | 17.774 | 19.362 |
|  | 19 | -4.3 | 24.428 | 26.689 |
|  | 20 | -4.3 | 9.461 | 12.019 |
